# Mul1 restrains Parkin-mediated mitophagy in mature neurons by maintaining ER-mitochondrial contacts

**DOI:** 10.1038/s41467-019-11636-5

**Published:** 2019-08-13

**Authors:** Rajat Puri, Xiu-Tang Cheng, Mei-Yao Lin, Ning Huang, Zu-Hang Sheng

**Affiliations:** Synaptic Function Section, The Porter Neuroscience Research Center, National Institute of Neurological Disorders and Stroke, National Institutes of Health, Room 2B-215, 35 Convent Drive, Bethesda, MD 20892-3706 USA

**Keywords:** Mitochondria, Cellular neuroscience

## Abstract

Chronic mitochondrial stress associates with major neurodegenerative diseases. Recovering stressed mitochondria constitutes a critical step of mitochondrial quality control and thus energy maintenance in early stages of neurodegeneration. Here, we reveal Mul1-Mfn2 pathway that maintains neuronal mitochondrial integrity under stress conditions. Mul1 deficiency increases Mfn2 activity that triggers the first phasic mitochondrial hyperfusion and also acts as an ER-Mito tethering antagonist. Reduced ER-Mito coupling leads to increased cytoplasmic Ca^2+^ load that activates calcineurin and induces the second phasic Drp1-dependent mitochondrial fragmentation and mitophagy. Overexpressing Mfn2, but not Mfn1, mimics Mul1-deficient phenotypes, while expressing PTPIP51, an ER-Mito anchoring protein, suppresses Parkin-mediated mitophagy. Thus, by regulating mitochondrial morphology and ER-Mito contacts, Mul1-Mfn2 pathway plays an early checkpoint role in maintaining mitochondrial integrity. Our study provides new mechanistic insights into neuronal mitochondrial maintenance under stress conditions, which is relevant to several major neurodegenerative diseases associated with mitochondrial dysfunction and altered ER-Mito interplay.

## Abstract (web summary)

Little is known about the pathways that maintain mitochondrial structure and function under neuronal stress conditions. Here, authors demonstrate that the Mul1-Mfn2 pathway plays a checkpoint role in maintaining mitochondrial integrity and energy maintenance by ensuring ER-mitochondrial tethering and preventing mitophagy.

## Introduction

Mitochondria supply ATP required for neuronal growth, survival, and function. Mitochondrial dysfunction and energy deficits are linked to major neurodegenerative diseases^1–3^. Mitophagy, a key pathway to eliminate damaged mitochondria^4^, is mediated by Parkinson’s disease (PD) genes PTEN-induced kinase 1 (PINK1; PARK6) and cytosolic E3 ubiquitin ligase Parkin (PARK2). Following mitochondrial depolarization, PINK1 is stabilized on the outer mitochondrial membrane (OMM), which leads to recruitment of cytosolic Parkin, triggering the ubiquitination of OMM proteins and the engulfment and degradation of damaged mitochondria through the lysosomal system. As neurons are post-mitotic cells that survive for the lifetime of the organism, recovery rather than acute degradation of stressed mitochondria is advantageous for mitochondrial maintenance and energy homeostasis.

Our previous study in mature cortical neurons revealed that mitophagy is observed in a small proportion of neurons following mitochondrial depolarization and that Parkin translocation to depolarized mitochondria occurs much more slowly than in non-neuronal cells^5,6^. Consistently, mitophagy is observed in a small proportion of axonal mitochondria following acute depolarization^7^. Parkin or PINK1 knock-out mice showed subtle changes in mitochondrial morphology and neuronal degeneration^8–10^. In *parkin* and *pink* mutant flies, the density and integrity of axonal mitochondria in motor neurons is comparable to WT controls^11,12^. These findings argue for intrinsic neuronal mechanisms that can maintain or recover mitochondrial integrity, rather than rapid elimination of dysfunctional mitochondria through Parkin-mediated mitophagy. To support this assumption, several fundamental questions remain to be addressed: (1) Do distinct mechanisms are in place in neurons to act as a checkpoint for recovery vs. rapid degradation of chronically stressed mitochondria? (2) Is mitophagy the second resort for neuronal mitochondrial quality control after recovery mechanisms have failed? (3) If this is the case, does mitochondrial ubiquitin ligase 1 (Mul1) play an early role in maintaining neuronal mitochondrial integrity? Addressing these questions is relevant to major neurodegenerative diseases associated with chronic mitochondrial stress.

Mul1, also referred to as mitochondrial-anchored protein ligase (MAPL)^13^, mitochondrial ubiquitin ligase activator of NF-κB (MULAN)^14^, or growth inhibition and death E3 ligase (GIDE)^15^, is a multifunctional mitochondrial membrane protein. In non-neuronal cell lines, Mul1 acts as an E3 ubiquitin ligase that binds, ubiquitinates, and degrades Mfn2^16^ and as a small ubiquitin-like modifier (SUMO) E3 ligase that SUMOylates dynamin-related protein-1 (Drp1) to enhance its stability on mitochondria^17,18^. Mfn2 is known to control mitochondrial fusion and regulate the interplay between the endoplasmic reticulum and mitochondria (ER-Mito)^19–21^ in mouse embryonic fibroblasts (MEFs) and Hela cells. ER-Mito contacts are tethered by multiple linker proteins with diverse distribution and interactions between the two organelles^22^. Among these linker proteins is tyrosine phosphatase interacting protein 51 (PTPIP51), which enhances ER-Mito interaction^23,24^. ER-Mito contacts maintain lipid and energy metabolism, as well as Ca^2+^ transfer from the ER to mitochondria that is essential for mitochondrial bioenergetics and integrity^25,26^. The ER extends from the soma into dendrites and axons^27^, thus allowing for distal ER-Mito signaling^28^. Disrupted ER-Mito contacts have been implicated in several major neurodegenerative diseases^23,29,30^.

PINK1 or Parkin null mutants in *Drosophila* show striking mitochondrial phenotypes^31^; these phenotypes are strongly suppressed by overexpressing Mul1^16^. Loss of Mul1 leads to increased Mfn1 and Mfn2 levels in HeLa cells^16^. Given the fact that Parkin-mediated mitophagy is significantly delayed in mature primary neurons in response to chronic mitochondrial stress under physiological and pathological conditions^6^, we hypothesize that neurons have an intrinsic mechanism for recovering chronically stressed mitochondria through the mitochondria-resident Mul1-Mfn pathway before recruiting cytosolic Parkin to damaged mitochondria. If this checkpoint mechanism fails, Parkin-mediated mitophagy is then activated. Here, by testing this hypothesis we reveal that the Mul1–Mfn2 pathway maintains neuronal mitochondrial integrity through its dual-role in regulating mitochondrial morphology and ER-Mito contacts. This mechanism ensures that mitophagy degradation is restrained in neurons under early stress conditions. Identifying this pathway is particularly relevant because chronical mitochondrial dysfunction and altered ER-Mito contacts have been reported in Alzheimer’s disease (AD), PD, amyotrophic lateral sclerosis (ALS), and hereditary spastic paraplegia (HSP)^23,32,33^.

## Results

### Mul1 protects neuronal mitochondria from mitophagy

While the PINK1-Parkin pathway in eliminating dysfunctional mitochondria has been well characterized in many cell types^34^, primary neurons often show delayed Parkin-mediated mitophagy in response to the depolarization of mitochondrial membrane potential (Δψ_m_)^5,6,35^. These studies raise the question of whether an alternative pathway acts as the first-line of surveillance that maintains or recovers neuronal mitochondrial integrity before activating mitophagy. Our hypothesis is that mitochondrial E3 ubiquitin ligase Mul1 plays an early role in maintaining neuronal mitochondrial integrity following mild stress before cytosolic E3 ubiquitin ligase Parkin is recruited to mitochondria for mitophagy. To test our hypothesis, we established a Mul1-deficient cortical neuron system by knocking down Mul1 expression with Mul1-targeted short hairpin RNA (Mul1-shRNA) or a scrambled hairpin RNA as a control (scr-shRNA); both were characterized in the previous study^16^. Mul1, but not mitochondrial proteins Hsp60 and Tom20, was largely suppressed in cells expressing Mul1-shRNA (Supplementary Fig. 1a). Mul1 has two mitochondrial transmembrane segments (TM1, TM2) and a RING finger domain with E3 ubiquitin ligase activity at the C-terminus. To generate Mul1-deficient cortical neurons, we expressed RING-deleted Mul1 mutant (Mul1ΔRing) or a single residue mutation in RING (Mul1-H319A). As an off mitochondria-targeting control, we also expressed a truncated Mul1 by deleting both TM1 and TM2 (Mul1ΔTM1/2) (Supplementary Fig. 1b). WT and mutant Mul1 (Mul1ΔRing and Mul1-H319A) are targeted to neuronal mitochondria labeled with cytochrome c or DsRed-Mito; Mul1ΔTM1/2 displays diffused distribution in neurons at days in vitro 10–11 (DIV10-11) (Supplementary Fig. 1c, d).

We asked whether Mul1 deficiency facilitates recruitment of cytosolic Parkin to stressed mitochondria in neurons. Acute mitochondrial depolarization with a high dose (40 μM) of antimycin A (AA), a respiratory complex III inhibitor, rapidly induces Parkin-mediated mitophagy in ~50 min^7^. To study the mitochondrial quality control in response to mild stress, we instead used 100 nM of AA, a 400-time lower dose than widely used in the literature^7,36^. We previously tested the effect of different dose of AA on mitochondrial integrity by measuring mitochondrial oxygen consumption rate (OCR) in cortical neurons^6^. Nanomolar dose AA induces mild mitochondrial stress relevant to chronic mitochondrial dysfunction associated with neurodegenerative diseases; this treatment is able to maintain neuronal survival, thus allowing us to perform various live imaging and cell biology studies. We measured Δψ_m_ with the cell-permeant, red-orange fluorescent cationic dye tetramethylrhodamine ethylester (TMRE), which labels polarized mitochondria with an inner Δψ_m_, following a 3-h treatment with 100 nM AA or DMSO control. This mild AA treatment is sufficient to depolarize mitochondria (Supplementary Fig. 2), allowing our study of mitochondrial maintenance.

**Fig. 1:**
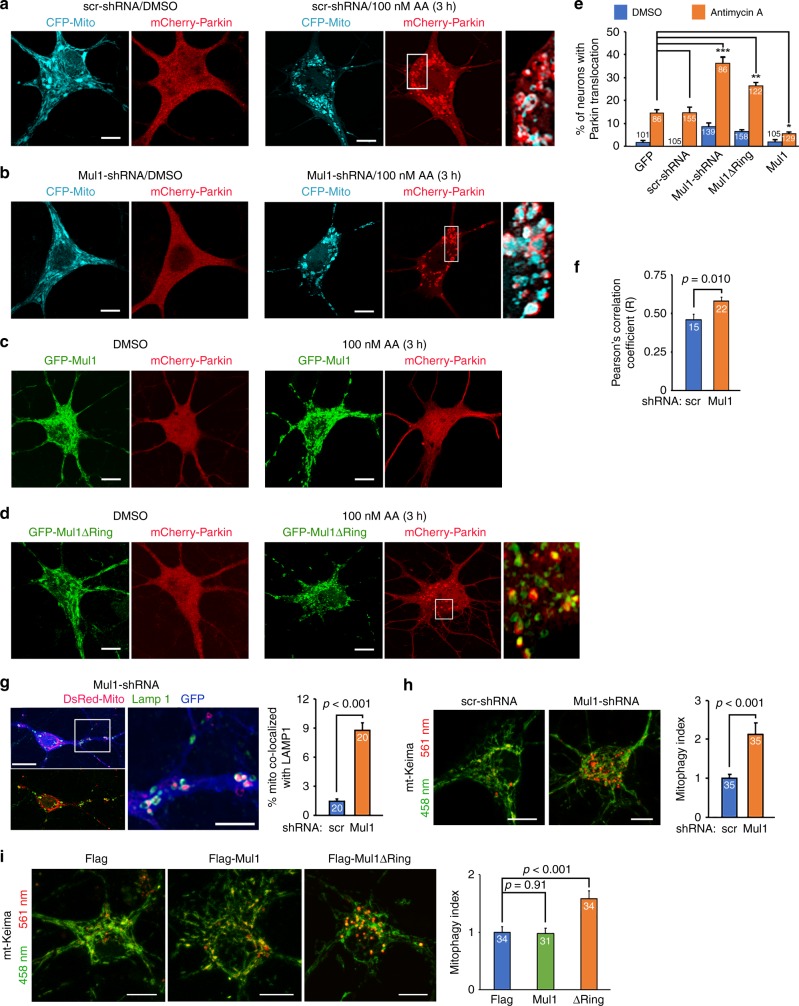
Mul1 restrains neuronal mitophagy under mild stress. **a**–**f** Enhanced Parkin recruitment to stressed mitochondria in Mul1-deficient neurons. Cortical neurons at DIV8 were co-transfected with mCherry-Parkin and CFP-Mito together with scr-shRNA, Mul1-shRNA, or co-transfected with mCherry-Parkin and GFP (as baseline), GFP-Mul1, or GFP-Mul1ΔRing, followed by incubation at DIV10 with 100 nM AA or DMSO for 3 h before fixation and imaging. Magnified inserts in boxes show colocalization of Parkin and mitochondria. Note that Mul1 deficiency facilitates more neurons to recruit cytosolic Parkin onto stressed mitochondria, resulting in a higher correlation of Parkin signal on mitochondria (**f**). To limit variations by unblinded selection, all transfected neurons in low-density culture (0.25 million per 10 cm^2^) were selected for analysis. **g** Endo-lysosomal targeting of fragmented mitochondria in Mul1-deficient neurons in the absence of AA treatment. Low-density cortical neurons were co-transfected using calcium phosphate method at DIV7 with Mul1-shRNA, DsRed-Mito, and GFP, followed by immunostaining with LAMP1 at DIV14. GFP signal (pseudo blue) was used to trace transfected neurons. 6–8 transfected neurons were chosen per dish per trial for each condition. **h**, **i** Enhanced dynamic mitophagy in Mul1-deficient neurons expressing Mul1-shRNA (**h**) or Mul1ΔRing (**i**). Cortical neurons at DIV7 were co-transfected with mt-Keima and various constructs as indicated, followed by live imaging at DIV14. mt-Keima is a ratiometric pH-sensitive fluorescent probe that targets to the mitochondrial matrix. A short wavelength (458 nm) is predominant for excitation in a neutral environment (mitochondrial signal green), whereas a long wavelength (561 nm) is predominant in an acidic environment (lysosomal signal red). “Mitophagy Index” was assessed by measuring the ratio of 561 nm/458 nm in the cell body and normalized to control conditions. Note that Mul1 deficiency enhances dynamic mitophagy. Data were analyzed from the total number of neurons indicated in the bars from three experiments and expressed as mean ± s.e.m. Unpaired Student’s *t*-test (**h**), Mann–Whitney test (**f**, **g**), or Ordinary one-way ANOVA with Dunnett multiple comparison test (**e**, **i**). (***p < 0.001; **p < 0.01; *p < 0.05). Scale bars: 5 µm (**a**–**d**) and 10 µm (**g**, **h**, **i**). (Also see Supplementary Figs. 1, 2, 3)

To examine whether Mul1 deficiency facilitates mitophagy, we measured the percentage of neurons displaying Parkin translocation under a mild depolarization condition^6^. Cortical neurons at DIV8 were co-transfected with mCherry-Parkin and CFP-Mito, together with GFP-Mul1, GFP-Mul1ΔRing, scr-shRNA, or Mul1-shRNA, followed by incubation at DIV10 with 100 nM AA for 3 h before fixation and imaging. Mild depolarization triggers Parkin translocation in a small portion of neurons expressing scr-shRNA or GFP-Mul1. In contrast, Mul1 deficiency induces more neurons to recruit Parkin into stressed mitochondria (Fig. 1a–e, Supplementary Fig. 2c). The average percentage of neurons displaying Parkin recruitment is 37.06 ± 2.47% (Mul1-shRNA, *p* < 0.001, Dunnett’s post hoc test) and 24.77 ± 2.21% (Mul1ΔRing, *p* < 0.01), respectively, relative to the baseline of neurons expressing GFP (14.84 ± 1.56%) following mild stress. Conversely, elevated Mul1 expression suppresses Parkin translocation (5.54 ± 0.67%, *p* < 0.05). Analysis of Pearson’s correlation coefficient indicates a correlation of Parkin signal on mitochondria in Mul1-shRNA neurons (Fig. 1f). Even in the absence of AA, Mul1 deficiency is sufficient to induce mitophagy in neurons, where fragmented mitochondria are readily observed; some of which are subjected to autophagic clearance by recruiting the autophagic markers p62 and LC3 (Supplementary Fig. 3) or sorted into lysosomes (Fig. 1g).

To further confirm enhanced mitophagy in Mul1-deficient neurons, we alternatively measured dynamic mitophagy using mt-Keima, a ratiometric pH-sensitive fluorescent probe that targets mitochondrial matrix^37^. Keima has an excitation spectrum that changes according to pH: a short wavelength (458 nm) is predominant for excitation in a neutral environment, whereas a long wavelength (561 nm) is predominant in an acidic lysosomal environment. When mitochondria are healthy, mt-Keima displays a low fluorescence ratio (561 nm/458 nm) (lysosomal signal red/mitochondrial signal green); however, when mitochondria are engulfed by acidic lysosomes, mt-Keima display a high fluorescence ratio (stronger red signal). Since mt-Keima is resistant to lysosomal proteases, it allows for measurement of dynamic and cumulative mitophagy processes. Analysis of “mitophagy index” by measuring relative ratio of 561 nm/458 nm in the cell body supports our notion that Mul1 deficiency enhances dynamic mitophagy in neurons with Mul1 depletion or overexpression of Mul1ΔRing (Fig. 1h, i). Altogether, these data suggest an essential role for Mul1 in suppressing neuronal mitophagy under mild stress conditions.

### Mul1 deficiency triggers biphasic mitochondrial dynamics

**Fig. 2:**
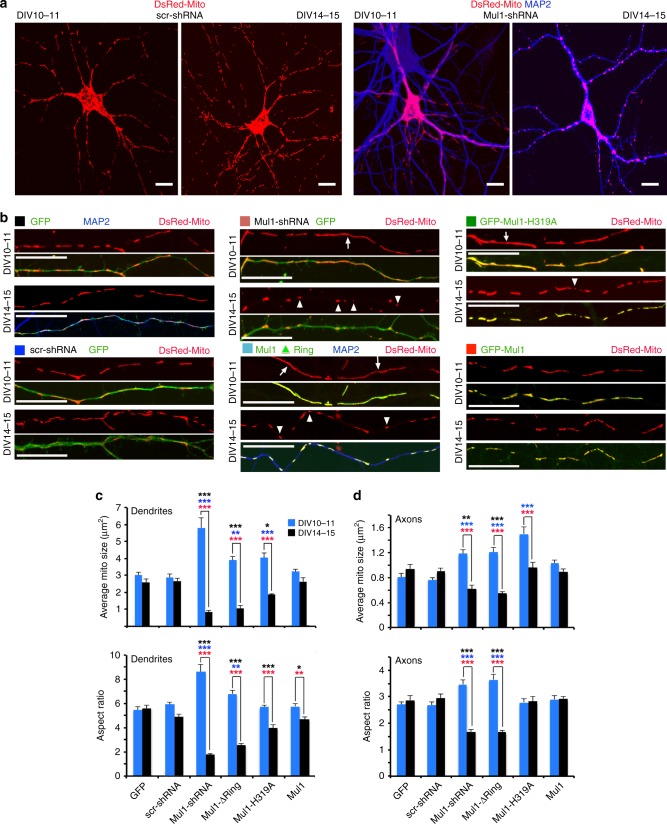
Mul1-deficient neurons display a biphasic change in mitochondrial morphology. **a** Transient mitochondrial hyperfusion followed by fragmentation in Mul1-depleted neurons. Cortical neurons were co-transfected at DIV7-8 with DsRed-Mito and control scr-shRNA or Mul1-shRNA, followed by immunostaining with MAP2 at DIV10-11 or DIV14-15. Note that neurons with Mul1 depletion display a transition from mitochondrial hyperfusion at DIV10-11 to fragmentation at DIV14-15. **b**–**d** Mitochondrial size and aspect ratio in dendrites and axons, across different conditions for phase I (DIV10-11) and phase II (DIV14-15). Cortical neurons at DIV7-8 were co-transfected with DsRed-Mito and GFP + scr-shRNA or GFP + Mul1-shRNA, or GFP-Mul1, Mul1ΔRing, or Mul1-H319A mutants, followed by imaging at DIV10-11 and DIV14-15. Note that the biphasic transition was consistently observed in Mul1-deficient neurons: hyperfused tubular mitochondria (arrows) at DIV10-11 and fragmented ones in dendrites (arrowheads) at DIV14-15 were observed in neurons transfected with Mul1-shRNA, Mul1ΔRing, or Mul1-H319A. Data were analyzed from more than three experiments and the total number of neurons for dendritic mitochondria groups (**c**): GFP (55), scr-shRNA (58), Mul1-shRNA (36), Mul1Δ Ring (58), Mul1-H319A (41), and Mul1 (62); and for axonal mitochondria groups (**d**): GFP (23), scr-shRNA (34), Mul1-shRNA (44), Mul1ΔRing (20), Mul1-H319A (20), and Mul1 (26). Data were expressed as mean ± s.e.m. Two-way ANOVA with Sidak’s multiple comparison test (**c**, **d**) was used for comparing two-time-phase groups (DIV10-11 vs DIV14-15) within each transfection condition (red ***p < 0.001; red **p < 0.01). Two-way ANOVA with Holm-Sidak’s multiple comparison test was used for comparing GFP control with other transfection conditions across the phase I group (DIV10-11: blue bars and blue ***p < 0.001; blue *p < 0.05), or across the phase II group (DIV14-15: black bars and black ***p < 0.001; black **p < 0.01). Scale bars: 10 µm (**a**), 5 µm (**b**). (Also see Supplementary Fig. 4, 5a–d)

Given that overexpression or depletion of Mul1 in non-neuronal cells perturbs mitochondrial dynamics^13,14^ and Mul1 promotes ubiquitin-dependent degradation of Mfn through its E3 ubiquitin ligase activity^16^, we asked whether Mul1 maintains neuronal mitochondrial morphology. By knocking down Mul1 in cortical neurons with Mul1-shRNA, we observed a striking mitochondrial phenotype: a biphasic transition from hyperfusion at DIV10-11 to fragmentation at DIV14-15. Such phenotypes are not observed in control neurons with scr-shRNA (Fig. 2a). We characterized the morphological transition by measuring the average mitochondrial size (area) and shape (aspect ratio of major/minor axis) at two-time points (DIV10-11 and DIV14-15) in both dendrites and axons. Elongated tubular mitochondria (arrows) at DIV10-11 and fragmented mitochondria (arrowheads) at DIV14-15 in both dendrites (Fig. 2b, c) and axons (Supplementary Fig. 4a) were readily observed in Mul1-deficient neurons transfected with Mul1-shRNA, Mul1ΔRing, or Mul1-H319A. Such phenotypes were rarely detected in control neurons expressing GFP or scr-shRNA. At DIV10-11, the average size of dendritic mitochondria in Mul1-deficient neurons varies from ≥4 µm^2^ (Mul1ΔRing, Mul1-H319A) to >5 µm^2^ (Mul1-shRNA) compared with ≤3 µm^2^ for control neurons (GFP or scr-shRNA). Increased size of dendritic mitochondria is attributed to mitochondrial elongation as indicated by an increase in the aspect ratio of >8 (Mul1-shRNA) or >6 for (Mul1ΔRing, Mul1-H319A) compared with <5 (GFP or sc-shRNA). Consistently, frequency distributions of mitochondrial size and aspect ratio show elongated mitochondria at DIV10-11 in both dendrites and axons in Mul1-deficient neurons (Supplementary Fig. 4b, c). However, the hyperfusion status (Phase I) is only maintained a few days; tubular mitochondria are quickly transitioned into fragmentation (Phase II) at DIV14-15. Mitochondrial fragmentation in Mul1-deficient neurons is mostly striking in dendrites, where mitochondria have an average size of ≤1 µm^2^ (Mul1-shRNA, Mul1ΔRing) to ≤2.5 µm^2^ (Mul1-H319A) and an aspect ratio ranging from <3 (Mul1 shRNA, Mul1ΔRing) to <4 (Mul1-H319A) (Fig. 2c, d). Knockdown of Mul1 showed more robust fragmentation than expressing mutant Mul1. Control neurons expressing GFP or scr-shRNA showed no detectable change in mitochondrial morphology, supporting our notion that Mul1 deficiency induces mitochondrial hyperfusion at phase I and fragmentation at phase II. While overexpressing Mul1 did not induce the biphasic change in mitochondrial morphology (Fig. 2b–d), fragmentation in Mul1-deficient neurons was partially reversed by re-introducing GFP-Mul1 (Supplementary Fig 4d).

To confirm the specificity of Mul1-shRNA, we generated an shRNA-resistant Mul1 mutant (Mul1*) by substituting eight third-code nucleotides in shRNA-targeting sequence without changing the amino acid. While Mul1-shRNA suppressed endogenous Mul1 and significantly depleted exogenous Flag-Mul1 (*p* < 0.001, Kruskal–Wallis-Test), it failed to suppress Mul1* (*p* = 0.68), thus indicating an shRNA-resistant Mul1 mutant (Supplementary Fig. 5a, b). Mitochondrial fragmentation and depolarization in Mul1-deficient neurons (Mul1-shRNA) can be effectively rescued by co-expressing Mul1* (Supplementary Fig. 5c–f). The rescue of two key Mul1-depleted phenotypes indicates the specificity of Mul1-shRNA, thus excluding an off-target effect.

### Fragmented mitochondria lose integrity and ATP production

**Fig. 3:**
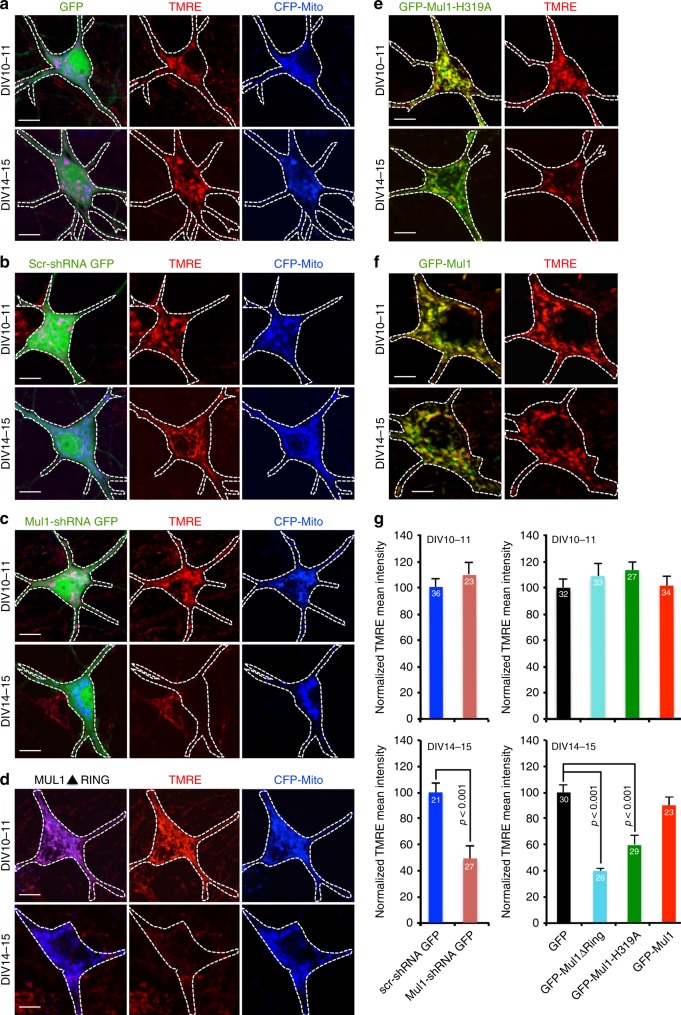
Mul1 deficiency reduces mitochondrial Δψ_m_ in mature neurons. Cortical neurons were co-transfected at DIV7-8 with GFP and CFP-Mito (**a**), GFP, CFP-Mito and scr-shRNA (**b**), GFP, CFP-Mito and Mul1-shRNA (**c**), CFP-Mito and Flag-Mul1ΔRing (**d**), GFP-Mul1-H319A (**e**), or GFP-Mul1 (**f**). Mitochondria were labeled by CFP-Mito (**a**–**d**: blue channel) or by mitochondria-targeted GFP-Mul1-H319A (**e**) or GFP-Mul1 (**f**) (green channel). Live imaging was performed at both DIV10-11 and DIV14-15 following loading of the Δψ_m_-dependent fluorescent dye TMRE (50 nm) for 20 min. Note that while neurons expressing GFP (**a**), scr-shRNA (**b**), or GFP-Mul1 (**f**) display TMRE-labeled polarized mitochondria at both DIV10-11 and DIV14-15, Mul1-deficient neurons (**c**–**e**) results in loss of TMRE staining of mitochondria at DIV14-15 (**g**), indicating mitochondrial depolarization. The edges of transfected cell bodies and proximal processes are marked with white dashed lines. TMRE mean intensity was measured and normalized to control neurons transfected with GFP at both time points. The data were analyzed from the total number of neurons shown in the bars from three experiments and expressed as mean ± s.e.m. Unpaired Student’s *t*-test was used for comparing two groups and Ordinary one-way ANOVA with Dunnett multiple comparison test for multiple comparisons. Scale Bars: 10 μm. (Also see Supplementary Figs. 5e, f, and 6)

We next asked whether the biphasic change in mitochondrial morphology is associated with altered mitochondrial integrity. We examined Δψ_m_ at DIV10-11 and DIV14-15, two-time points associated with hyperfusion and fragmentation, respectively. Mitochondria were labeled by CFP-Mito (Fig. 3a–d), GFP-Mul1-H319A (Fig. 3e), or GFP-Mul1 (Fig. 3f). Live neurons at both DIV10-11 and DIV14-15 were loaded with TMRE. TMRE intensity was measured in somato-dendritic mitochondria. Control neurons expressing GFP, scr-shRNA + GFP, or GFP-Mul1 display polarized mitochondria at DIV10-11 and DIV14-15. While TMRE intensity is maintained at DIV10-11 in Mul1-deficient neurons, it is significantly reduced at DIV14-15 (Fig. 3g), suggesting that hyperfused mitochondria are able to maintain their Δψ_m_, whereas fragmented ones largely lose their Δψ_m_.

Mitochondria ATP production depends upon the maintenance of Δψ_m_. To assess ATP production capacity in Mul1-deficient neurons, we measured cellular ATP levels using a genetically encoded ratiometric FRET-based ATP sensor (GoAteam2)^38^, as we previously reported^39^. Neurons were co-transfected with GoAteam2 and Mul1 or Mul1ΔRing at DIV7-8. The relative ATP levels were measured by analysis of the emission ratio (560/510) when excited with a 458-nm laser line at both DIV10-11 and DIV14-15. A significant reduction in cellular ATP levels was detected in neurons expressing Mul1ΔRing in the soma-dendritic region, dendrites, and axons (Supplementary Fig. 6a–d) at DIV14-15 when compared to DIV10-11. No significant change in ATP levels was detected in neurons expressing Flag vector or WT Mul1 between these two-time points. These data indicate that Mul1 deficiency impairs mitochondrial integrity and thus ATP production at DIV14-15, a time point associated with mitochondrial fragmentation. To further confirm mitochondrial dysfunction in bioenergetics, we used the Seahorse XF analyzer to examine the oxygen consumption rate (OCR). Basal and real-time OCR were significantly reduced in Mul1-deficient neurons compared to control (Supplementary Fig. 6e, f).

Our previous study indicated that cortical neurons from *parkin* KO mice showed no significant changes in mitochondrial membrane potential and neuronal morphology as compared with wild-type neurons^16^. However, Mul1 knockdown in *parkin* KO neurons resulted in a loss of Δψ_m_ in the early stage (DIV10-11) and dendritic and axonal neurodegeneration. To assess the effect of reduced bioenergetic capacity on neuronal physiology, we examined dendritic arborizations in Mul1-deficient neurons by performing Sholl analysis. A significant decrease in dendritic arborization pattern was observed in Mul1-deficient neurons at DIV14-15 compared to control neurons (*p* < 0.001, two-way ANOVA test) (Supplementary Fig. 7), indicating that Mul1 deficiency affects neuronal cell connectivity and survival.

### Increased Mfn2 activity mediates Mul1-deficient phenotypes

**Fig. 4:**
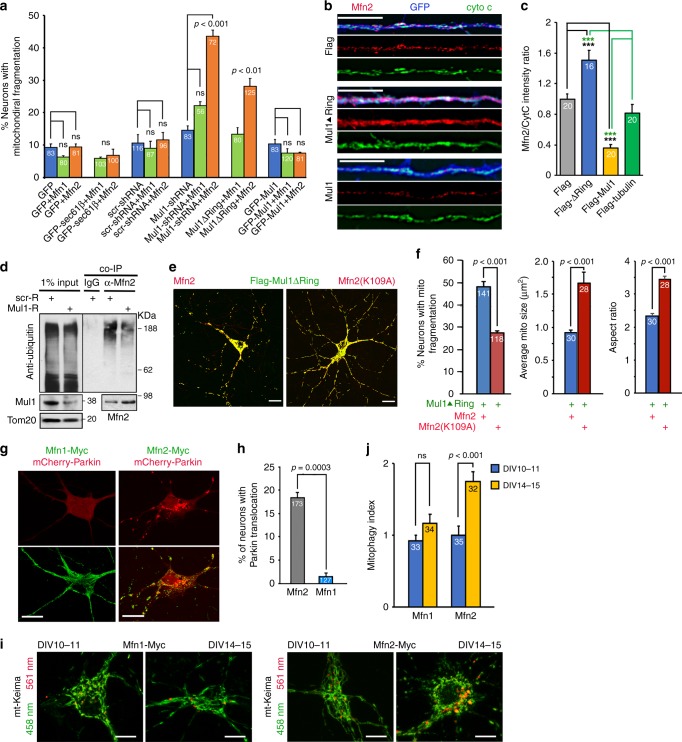
Elevated Mfn2 activity mediates Mul1-deficient phenotypes. **a** Accelerated mitochondrial fragmentation at DIV10 following elevated Mfn2 expression in Mul1-deficient neurons. Cortical neurons at DIV7 were co-transfected with various combinations of expression vectors as indicated, followed by imaging mitochondria at DIV10. **b**, **c** Significant changes in Mfn2 levels in dendrites of Mul1-deficient neurons. Cortical neurons at DIV7 were co-transfected with various combinations of expression vectors as indicated, followed by co-immunostaining of Mfn2 and cytochrome c (cyto c) at DIV10. GFP signals (pseudo blue) were used to trace transfected neurons. Relative Mfn2 levels were determined by measuring the intensity ratio of Mfn2/cyto c and normalized to control neurons expressing Flag. Note that expressing Flag-Mul1ΔRing increases normalized Mfn2/cyto c intensity ratio while expressing Mul1 reduces the ratio relative to control neurons expressing Flag alone (*p* < 0.001, black ***) or Flag-tubulin (*p* < 0.001, green ***). **d** Reduced Mfn2 ubiquitination and increased Mfn2 levels in Mul1-depleted cortical neurons. Neurons were transduced with Mul1-shRNA or scr-shRNA, followed by sequential blotting on the same blots with antibodies as indicated. **e**, **f** Reduced mitochondrial fragmentation by expressing Mfn2(K109A). Cortical neurons were co-transfected at DIV7-8 with Flag-Mul1ΔRing and Mfn2-Myc or Mfn2(K109A)-Myc, followed by co-immunostaining at DIV10-11. The percentage of neurons with mitochondrial fragmentation was determined from the total number of neurons indicated in the bars. Average mitochondrial size and aspect ratio were analyzed from a total of 8423 and 8354 mitochondria, respectively. **g**, **h** Enhanced Parkin translocation to mitochondria upon overexpression of Mfn2, but not Mfn1, in the absence of AA. Cortical neurons were co-transfected at DIV7-8 with mCherry-Parkin and Myc-tagged Mfn2 or Mfn1, followed by immunostaining Myc-tag at DIV14-15. **i**, **j** Enhanced mitophagy process upon overexpression of Mfn2, but not Mfn1, at DIV14-15. Data were analyzed from the total number of neurons indicated in the bars and expressed as mean ± s.e.m. Unpaired Student’s *t-*test was used for comparing two groups (**f**, **h**, **j**) and Ordinary one-way ANOVA with Dunnett’s post hoc test (**a**) or Tukey’s multiple comparison test (**c**) for multiple comparisons. Scale bars: 10 μm. (Also see Supplementary Fig. 8)

Deleting Mul1 in *Drosophila* and suppressing Mul1 in HeLa cells lead to increased Mfn levels^16^. We sought to determine which isoform Mfn1 or Mfn2 plays a prominent role in mediating Mul1-deficient phenotypes. We hypothesize that elevated Mfn1 or Mfn2 in Mul1-deficient neurons accelerates the bi-phasic transition. Expressing either Mfn1 or Mfn2 has a subtle impact on mitochondrial fragmentation in control neurons. However, elevated Mfn2 expression significantly accelerates the transition of mitochondria into fragmentation at DIV10 in Mul1-deficient neurons with Mul1-shRNA (*p* < 0.001, Dunnett’s post hoc test) or Mul1ΔRing (*p* < 0.01) compared to Mfn1 expression (Fig. 4a). There was no significant change in percentage of neurons with mitochondrial fragmentation between groups of (1) Mfn1 vs Mfn2; (2) Sec61β/Mfn1 vs Sec61β/Mfn2; (3) scr-shRNA/Mfn1 vs scr-shRNA/Mfn2; and (4) Mul1/Mfn1 vs Mul1/Mfn2. Therefore, these data support that Mfn2, but not Mfn1, accelerates mitochondrial fragmentation in Mul1-deficient neurons even at an early stage (DIV10). Given that both Mfn1 and Mfn2 play a similar role in mitochondrial fusion, Mfn2 mediates Mul1-deficient phenotypes likely through its non-fusion action.

After validating the specificity of the anti-Mfn2 antibody in cortical neurons by knocking down Mfn2 expression using Mfn2-shRNA-1 or Mfn2-shRNA-2 (Supplementary Fig. 8a-c), we determined whether mitochondria-targeted Mfn2 is increased in Mul1-deficient neurons by measuring the Mfn2/cyto c intensity ratio in dendritic processes. Expressing Mul1ΔRing significantly increases the Mfn2/cyto c intensity ratio (*p* < 0.001, Tukey’s post hoc test) while overexpressing Mul1 reduces the ratio (*p* < 0.001) relative to control neurons expressing Flag alone or a non-specific Flag-tubulin (Fig. 4b, c; Supplementary Fig. 8d, e). Second, we sought to establish the causal role of Mfn2 in mediating the Mul1-deficient phenotypes by knocking down Mfn2. Cortical neurons were transfected with Mul1ΔRing at DIV7-8, followed by a second transfection at DIV10-11 with Mfn2-shRNA-1, Mfn2-shRNA-2, or control scr-shRNA. Depleting Mfn2 in Mul1-deficient neurons rescued mitochondrial fragmentation and size (Supplementary Fig. 8f–h). Third, we performed biochemical analysis showing reduced Mfn2 ubiquitination and increased Mfn2 levels in Mul1-depleted neurons when compared to control neurons (Fig. 4d). In addition, we compared the effect of Mfn2 and a catalytically inactive mutant Mfn2 (K109A) on mitochondrial fragmentation in Mul1-deficient neurons^40^. While expressing Mfn2 triggers an early transition of mitochondria into fragmentation at DIV10, expressing Mfn2 (K109A) reduces mitochondrial fragmentation at DIV14-15 (Fig. 4e, f). To confirm whether elevated Mfn2 mediates Mul1-mutant phenotypes, we expressed Mfn2 alone in control neurons in the absence of stress. Expressing Mfn2, but not Mfn1, triggers mitochondrial fragmentation and Parkin recruitment (Fig. 4g, h). We further measured the dynamic mitophagy using mt-Keima. Neurons with Mfn2 overexpression displayed enhanced lysosomal targeting of mt-Keima over a time course from DIV10-11 to DIV 14-15 (*p* < 0.001, unpaired Student’s *t*-test) (Fig. 4i, j). In contrast, expressing Mfn1 has no effect in inducing mitophagy during the same time course. Altogether, these results suggest that elevated Mfn2 activity mediates Mul1-deficient phenotypes.

### Elevated Mfn2 impairs ER-Mito contacts

**Fig. 5:**
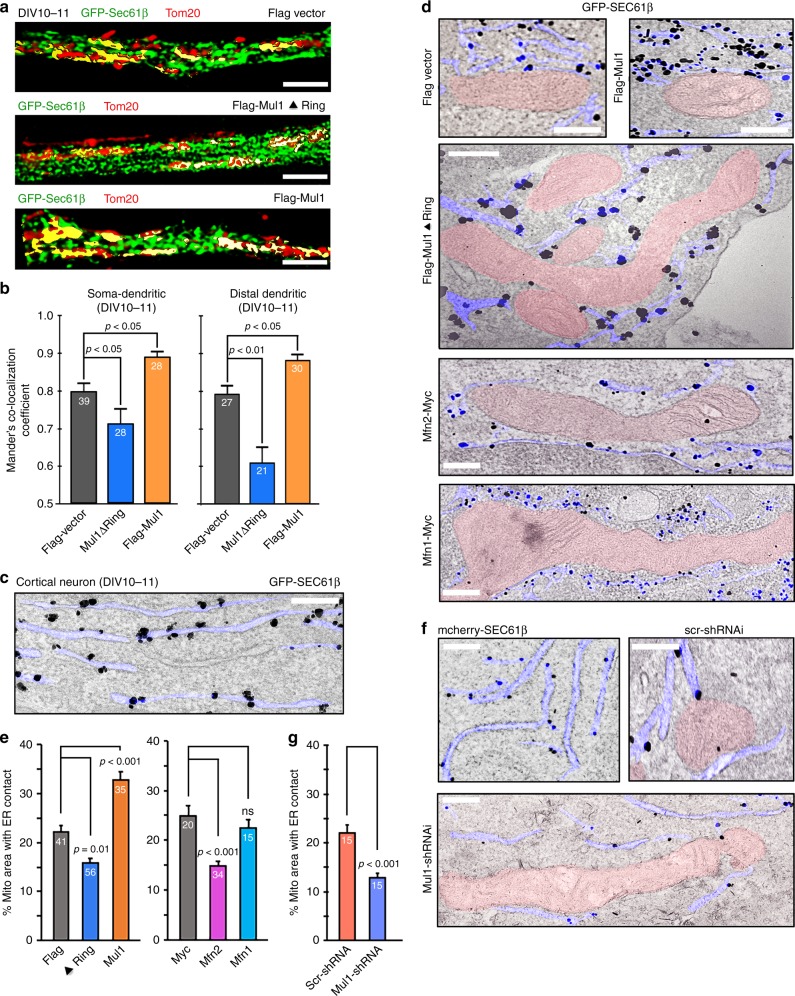
Mul1 deficiency reduces ER-Mito contacts. **a**, **b** Representative STED images and quantitative analysis showing reduced ER-Mito contacts in dendrites of Mul1-deficient neurons. Cortical neurons were co-transfected at DIV7 with the ER marker GFP-Sec61β and Flag, Flag-Mul1ΔRing, or Flag-Mul1. Neurons were fixed at DIV11 and immunostained for Tom20. Super-resolution images were captured using dual-color 3D-STED nanoscopy. The relative colocalization between the ER and mitochondria was measured in both somato-dendritic and distal dendritic regions with a plug-in from Volocity Software and expressed as Mander’s colocalization coefficient. The yellow color represents overlap between the ER and mitochondria. Note that expressing Mul1ΔRing significantly reduces ER-Mito contacts while expressing Mul1 enhances the contacts relative to Flag baseline control. **c**–**g** Representative iTEM graphs and quantitative analysis showing reduced ER-Mito contacts in Mul1-deficient neurons. Neurons at DIV7-8 were co-transfected with GFP-Sec61β and Flag, Flag-Mul1, Flag-Mul1ΔRing, Myc, Mfn1-Myc or Mfn2-Myc, followed by immunostaining with an anti-GFP antibody (**c**, **d**); or co-transfected with mcherry-Sec61βb and scr-shRNA or Mul1-shRNA followed by immunostaining with an anti-mCherry antibody (**f**) and stained with a 10-nm gold-labeled secondary antibody at DIV10-11. A representative iTEM image shows specific gold-labeling of ER membranes with an antibody against GFP (**c**) or mCherry (**f**). The ER tubular structures were colored with light blue while mitochondria were colored with light pink. The percentage of mitochondrial surface area contacting with ER were quantified by measuring the distance within a range of <12 nm between ER and mitochondrial outer membrane. Note that expressing Flag-Mul1ΔRing, Myc-Mfn2, or Mul1-shRNA significantly reduces ER-Mito contacts relative to control. All data were analyzed from the total number of neurons (**b**) or mitochondria (**e**, **f**) shown in the bars in three experiments and expressed as mean ± s.e.m. Ordinary one-way ANOVA with Dunnett’s post hoc test (**b**, **e**) and Mann–Whitney *U*-test (**g**). Scale Bars: 1 μm (**a**) or 100 nm (**c**, **d**, **f**)

ER-Mito contacts are important to maintain energy metabolism and Ca^2+^ transfer from the ER to mitochondria^25,26,33,41^. While Mfn1 mainly resides on mitochondria, Mfn2 is sorted to both the mitochondrial OMM and ER, where Mfn2 has been reported to regulate ER-Mito contacts in non-neuronal cells^19–22^. We sought to examine whether Mul1 deficiency alters ER-Mito contacts before mitochondrial fragmentation occurs. First, we measured ER-Mito contact sites in neurons by applying 3D diffraction-unlimited stimulated emission depletion (3D-STED) nanoscopy that can reach a lateral resolution of 30–70 nm. ER structures were labeled by ER membrane marker GFP-Sec61β and mitochondria were stained with an antibody against Tom20. ER-Mito contact sites were measured at DIV10-11 by calculating Mander’s colocalization coefficient. Cortical neurons expressing Mul1ΔRing display reduced ER-Mito contacts (*p* < 0.05 or *p* < 0.01, one-way ANOVA) in soma-dendritic or distal dendritic regions, respectively, while expressing Mul1 enhances ER-Mito contacts relative to control (*p* < 0.05) (Fig. 5a, b, Supplementary Videos 1–3). Since expressing Mul1 does not result in a dramatic change in mitochondrial morphology (Fig. 2b–d), Mul1 likely has a primary role in regulating ER-Mito contact under normal physiology.

We next confirmed the super-resolution imaging with immuno-transmission electron microscopy (iTEM) analysis. First, the ER targeting of GFP-Sec61β was examined by immunostaining cortical neurons with an anti-GFP antibody and a 10-nm gold-labeled secondary antibody. Gold-labeled GFP-Sec61β is selectively scattered along the surface of ER membranes (Fig. 5c). The ER-Mito contact area were measured if the distance between the two organelles was <12 nm, as previously reported^19–21^. While overexpressing Mul1 enhances ER-Mito contact (*p* < 0.001, Dunnett’s post hoc test) relative to control neurons, expressing Mul1ΔRing or knocking down Mul1 in neurons significantly reduces ER-Mito contact (*p* = 0.01, *p* < 0.001, respectively, Mann–Whitney *U*-test) (Fig. 5d–g). In addition, overexpressing Mfn2, but not Mfn1, also inhibits ER-Mito contact (*p* < 0.001, Dunnett’s post hoc test). Altogether, our super-resolution imaging and ultrastructural analysis consistently indicate that Mul1 protects neuronal mitochondria integrity by maintaining ER-Mito contacts; impaired ER-Mito contacts in Mul1-deficient neurons is mediated through elevated Mfn2 expression.

**Fig. 6:**
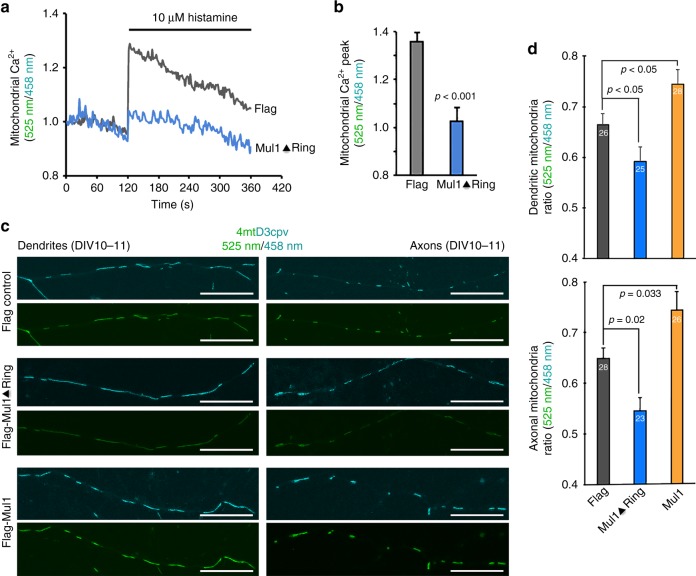
Mul1 deficiency reduces mitochondrial Ca^2+^ uptake from the ER. **a**, **b** Representative traces and quantitative analysis of the average maximal Ca^2+^ peak showing impaired mitochondrial Ca^2+^ uptake from the ER in Mul1-deficient neurons. Cortical neurons were co-transfected at DIV7-8 with pcDNA-4mtD3cpv and Flag or Flag-Mul1ΔRing, followed by live imaging at DIV10-11. The emission spectra of the ratiometric probe (525 nm/458 nm) were obtained by exciting 405 nm following stimulation with 10 μM histamine. **c**, **d** Reduced Ca^2+^ load in both axonal and dendritic mitochondria in Mul1-deficient neurons. The pseudo colors cyan (458 nm) and green (525 nm) represent the emission spectra of the ratiometric probe when excited with 405 nm. The images were captured from dendritic and axonal compartments as indicated. The mitochondrial Ca^2+^ load was expressed as a ratio (525 nm/458 nm). Note that expressing Mul1ΔRing significantly reduces the ratio while expressing Mul1 enhances the ratio. Data were analyzed from 2 to 3 live neurons per field with 4 fields per experiment (**b**), or total number of fixed neurons indicated in the bars (**d**) in three experiments and expressed as mean ± s.e.m. Unpaired Student’s *t*-test was used for comparing two groups and Ordinary one-way ANOVA with Dunnett multiple comparison test for multiple comparisons. Scale Bars: 10 μm

ER-Mito contacts play an important role in Ca^2+^ transfer from ER to mitochondria that is essential for bioenergetics^25,26,33,41^. Thus, temporal measurement of Ca^2+^ uptake by mitochondria following physiological Ca^2+^ release from ER is an important functional readout of ER-Mito coupling^42,43^. We applied a genetically encoded FRET-based ratiometric Ca^2+^ probe (4mtD3cpv) that specifically targets the mitochondrial matrix^44,45^. The emission spectra (525 nm/458 nm) were obtained by exciting 405 nm before and upon stimulation with 10 μM histamine. Mul1-deficient neurons display a reduced mitochondrial Ca^2+^ peak (*p* < 0.001, unpaired Student’s *t*-test) following histamine-induced release from the ER (Fig. 6a, b). Alternatively, we constructed a non-tagged Mul1-shRNA for calcium imaging experiment. Depleting Mul1 in neurons reduces mitochondrial Ca^2+^ uptake from the ER (Supplementary Fig. 9). We further examined the mitochondrial Ca^2+^ load in both dendritic and axonal compartments at DIV10-11. The pseudo colors cyan (458 nm) and green (525 nm) were imaged when excited with 405 nm. The mitochondrial Ca^2+^ load was expressed as a ratio (525 nm/458 nm). Expressing Mul1ΔRing significantly reduces the ratio in both dendritic and axonal regions (*p* < 0.01, Dunnett’s post hoc test) while expressing Mul1 enhances the ratio (*p*< 0.05), indicating an altered mitochondrial Ca^2+^ load (Fig. 6c, d). Mul1-deficient neurons, where hyperfused mitochondria are readily detected at DIV10-11, display reduced mitochondrial Ca^2+^ uptake from the ER, reflecting an impaired ER-Mito interplay.

### Impaired ER-Mito contact induces mitochondrial fragmentation

**Fig. 7:**
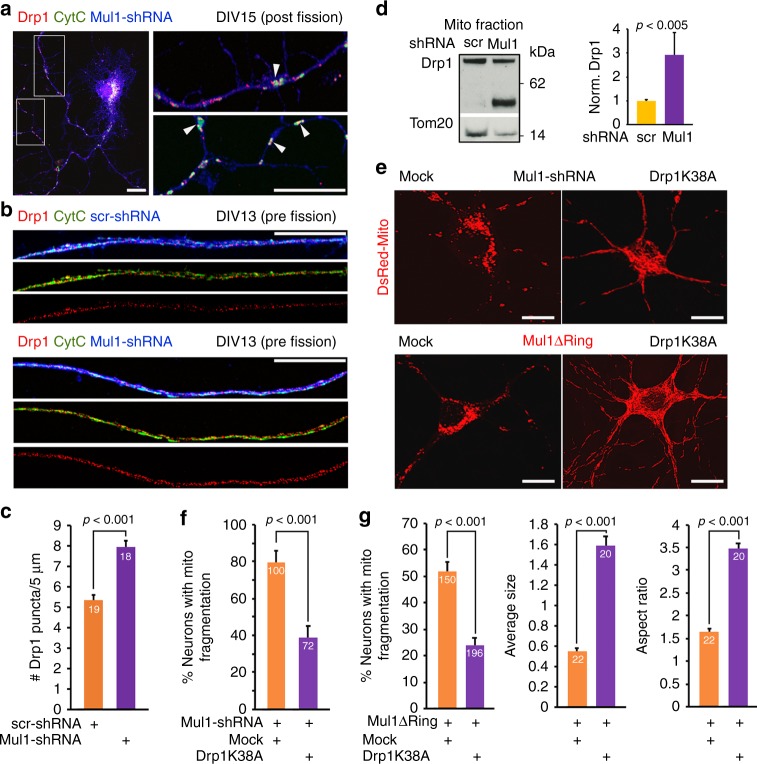
Drp1 mediates second phasic mitochondrial fragmentation. **a**–**c** Representative images showing the targeting of Drp1 to fragmented mitochondria in Mul1-deficient neurons. Cortical neurons at DIV7-8 were co-transfected with GFP and Mul1-shRNA or scr-shRNA, followed by co-immunostaining of Drp1 (red) and cyto c (green) at DIV14-15 (**a**) or DIV13 (**b**) (pre-fission status). GFP (pseudo blue) was used to trace transfected neurons. Arrows point to Drp1 puncta on fragmented mitochondria (**a**). Average number of Drp1 puncta per 5 µm mitochondrial length was measured from the total number of mitochondria (scr-shRNA, *n* = 518; Mul1-shRNA, *n* = 423) in the total number of neurons indicated in the bars. **d** A representative immunoblot showing an increased Drp1 translocation to mitochondria in Mul1 depleted neurons. Equal amounts (15 µg) of mitochondrial fractions were sequentially immunoblotted on the same membrane with each antibody after stripping. Note that a shorter Drp1 isoform (~58 kDa) was recruited into mitochondrial fraction in Mul1-deficient neurons. **e**–**g** Drp1-dependent mitochondrial fragmentation in Mul1-deficient neurons. Cortical neurons at DIV7-8 were co-transfected with Mul1-shRNA, DsRed-Mito and Myc-tagged plasmid (Mock) or Drp1K38A, or co-transfected with Flag-tagged Mul1ΔRing and Mock or Drp1K38A, followed by fixation and imaging at DIV14-15. Note that expressing Drp1K38A locks mitochondrial in hyperfusion status in the late stage of Mul1-deficient neurons. All data were analyzed from the total number of neurons indicated in the bars in three experiments and are expressed as mean ± s.e.m. Unpaired Student’s *t*-test. Scale Bars: 10 μm

**Fig. 8:**
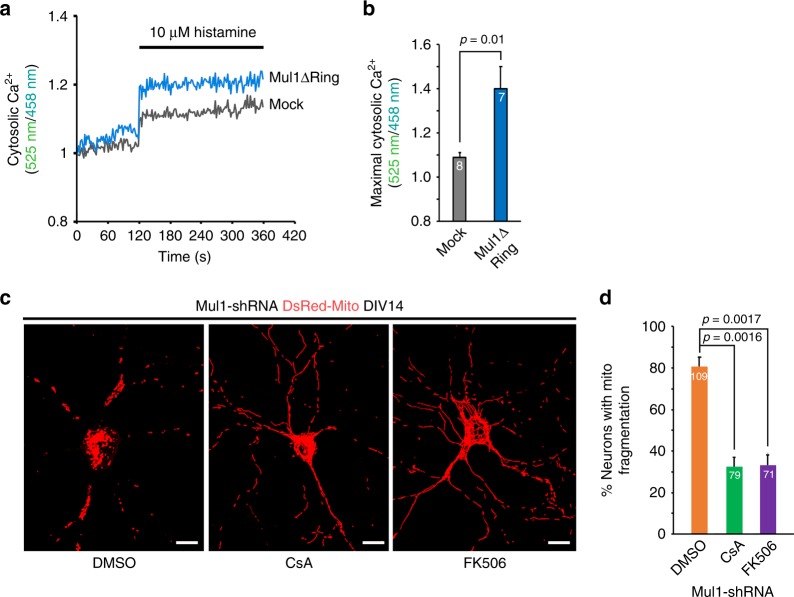
Increased cytoplasmic Ca^2+^ load induces mitochondrial fragmentation. **a**, **b** Representative traces and quantitative analysis of the average cytoplasmic Ca^2+^ peak showing enhanced cytoplasmic Ca^2+^ load in Mul1-deficient neurons. Cortical neurons were co-transfected at DIV7-8 with pcDNA-D3cpv and Flag or Flag-MUL1ΔRing, followed by live imaging at DIV12-13 just before mitochondrial fragmentation. The emission spectra of the ratiometric probe (525 nm/458 nm) were obtained by exciting 405 nm following stimulation with 10 μM histamine. Note that Mul1-deficient neurons display an increased cytoplasmic Ca^2+^ peak (*p* = 0.01) following histamine-induced Ca^2+^ release from the ER. Data were analyzed from 2 to 3 live neurons per field with 3 to 4 fields per experiment and the number of trials is indicated in the bars. **c**, **d** The role of calcineurin activity in mitochondrial fragmentation in Mul1-depleted neurons. Cortical neurons, co-transfected with DsRed-Mito and Mul1shRNA at DIV7-8, were treated with CsA (0.5 μM) or FK506 (0.6 μM) for 48 h starting at DIV12. Note that blocking calcineurin activity by CsA or FK506 abolishes mitochondrial fragmentation in Mul1-depleted neurons at DIV14. All data were analyzed from the total number of neurons indicated in the bars in three experiments and are expressed as mean ± s.e.m. Unpaired Student’s *t*-test (**b**) or Ordinary one-way ANOVA with Dunnett’s post hoc test (**d**). Scale Bars: 10 μm

By immunostaining cortical neurons with an anti-Drp1 antibody at DIV14-15, we determined whether Drp1 is recruited to OMM after ER-Mito contacts are impaired. Drp1 puncta mainly co-localize with fragmented mitochondria, suggesting Drp1 recruitment for fission. Depleting Mul1 significantly enhances the density of Drp1 puncta along dendritic mitochondria (Fig. 7a–c). To confirm the role of Drp1 in the second phasic mitochondrial fragmentation, we performed mitochondrial fractionation of WT and Mul1-depleted neurons at DIV12-13. An increased recruitment of total Drp1 (*p* < 0.005, unpaired Student’s *t-*test) into the mitochondrial fraction was detected in Mul1-depleted neurons when compared to WT neurons (Fig. 7d). The *drp1* gene contains several alternative exons and produces multiple isoforms through RNA splicing^46^, in which the shorter isoform named isoform 12 encoding 587 residues contains all functional domains. This shorter isoform (~58 kDa) is robustly increased in the mitochondrial fraction in Mul1-deficient neurons. Interestingly, recruitment of this isoform into mitochondria is largely suppressed in control neurons (Fig. 7d). In addition to the long Drp1 isoform, it is likely that this shorter Drp1 isoform plays a role in the second phasic mitochondrial fragmentation. Expressing Drp1 loss-of-function mutant Drp1K38A in Mul1-deficient neurons locks mitochondria in the hyperfusion status at DIV14-15, while expressing Mul1-shRNA or Mul1ΔRing alone display robust mitochondrial fragmentation at the same stages (Fig. 7e–g). Thus, our study supports that the second phasic fragmentation is a Drp1-dependent process. Next, we asked how Drp1 is activated to trigger mitochondrial fragmentation in Mul1-deficient neurons. Drp1 recruitment and fission activity is primarily regulated by post-translational modifications. Increased cytoplasmic Ca^2+^ load activates calcineurin phosphatase which dephosphorylates Drp1 at serine 637 and thus activates Drp1^47^. Our immunoblot analysis confirmed a suppressed phosphorylation status of Drp1 in Mul1-depleted neurons (Supplementary Fig. 8i). These findings suggest that defective ER-Mito coupling builds cytoplasmic Ca^2+^ load that subsequently activates Drp1. To test this, we expressed a cytoplasm-targeted FRET-based ratiometric Ca^2+^ probe (D3cpv)^44,45^ in cortical neurons at DIV7-8, followed by live imaging at DIV12-13 just before mitochondrial fragmentation occurs. Mul1-deficient neurons display an increased cytoplasmic Ca^2+^ peak (*p* = 0.01, unpaired Student’s *t-*test) following histamine-induced release from the ER (Fig. 8a, b).

We further examined whether calcineurin activity is associated with mitochondrial fragmentation by applying two inhibitors of calcineurin, cyclosporine A (CsA)^48^ and FK506^49^. Cortical neurons, co-transfected with DsRed-Mito and Mul1shRNA or scr-shRNA at DIV7-8, were treated with CsA (0.5 μM) or FK506 (0.6 μM) for 48 h starting at DIV12. Strikingly, fragmentation was largely abolished in Mul1-depleted neurons following either treatment of CsA or FK506 (Fig. 8c, d). Blocking calcineurin activity locks mitochondria in the hyperfusion status at DIV14. Thus, our study provides new mechanistic insights as to how impaired ER-Mito interplay triggers mitochondrial fragmentation through elevated cytoplasmic Ca^2+^ load and calcineurin activity.

### Enhancing ER-Mito coupling suppresses neuronal mitophagy

**Fig. 9:**
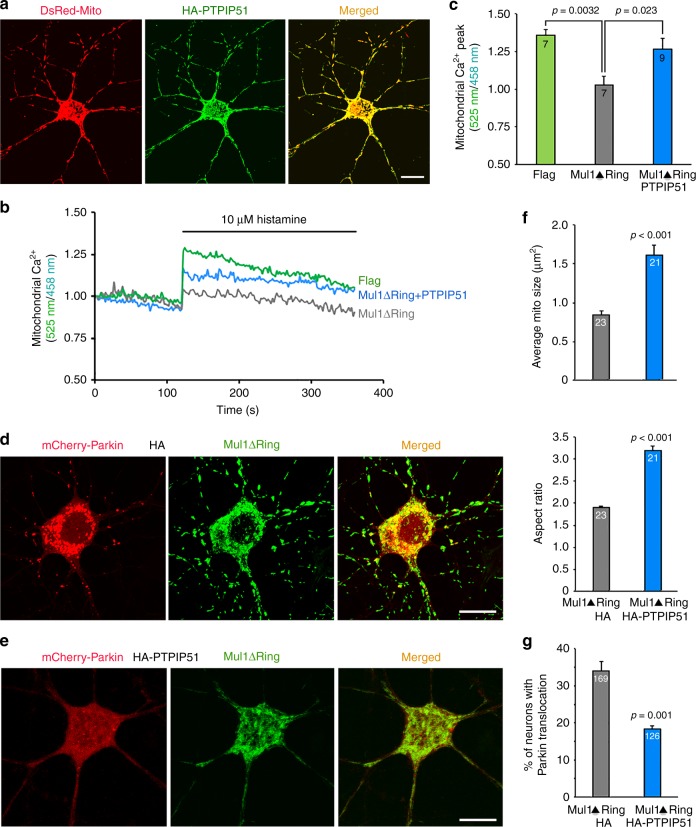
Expressing PTPIP51 suppresses mitochondrial fragmentation and Parkin translocation in Mul1-deficient neurons. **a** Representative images showing the mitochondria-targeting of PTPIP51 in cortical neurons. Neurons were co-transfected with HA-tagged PTPIP51 and DsRed-Mito at DIV7-8 and imaged at DIV10-11 by immunostaining HA-tag. **b**, **c** Representative traces and quantitative analysis showing a partial rescue of IP3-induced ER-Mito Ca^2+^ exchange by expressing PTPIP51 in Mul1-deficient neurons. Cortical neurons at DIV7-8 were co-transfected with pcDNA-4mtD3cpv and Flag, or Flag-Mul1ΔRing, or Flag-Mul1ΔRing + HA-PTPIP51. pcDNA-4mtD3cpv is a FRET-based ratiometric calcium probe that targets the mitochondrial matrix. Live neurons were imaged at DIV10-11. The traces represent the average fluorescence ratio signal (525 nm/458 nm) when excited with a 405-nm laser following stimulation with 10 μM histamine. Mitochondrial Ca^2+^ levels were measured from the total number of trials indicated in the bars. Each trial denotes 2–3 neurons per field. **d**–**g** PTPIP51 expression suppresses mitochondrial fragmentation and reduces Parkin translocation in Mul1-deficient neurons. Cortical neurons at DIV7-8 were co-transfected with mCherry-Parkin, Flag-Mul1ΔRing, and HA-Vector (**d**) or HA-PTPIP51 (**e**), followed by immunostaining with Flag-tag (green) at DIV14-15. The mitochondrial morphology was measured as average mitochondria size and aspect ratio from the total number of neurons indicated in the bars (**f**); the percentage of neurons with Parkin recruitment to fragmented mitochondria was measured from the total number of neurons indicated in the bars (**g**). All data were from three experiments using the unpaired Student’s *t*-test (**f, g**) and Ordinary one-way ANOVA with Dunnett’s post hoc test (**c**) and are expressed as mean ± s.e.m. Scale Bars: 10 μm

ER-Mito contacts are tethered by proteins between the two organelles, including mitochondrial protein PTPIP51 and ER protein VAPB^23^. To support our conclusion, we sought to enhance the ER-Mito coupling in Mul1-deficient cortical neurons by co-expressing HA-tagged PTPIP51. First, we confirmed mitochondria-targeting of PTPIP51 in neurons (Fig. 9a). Second, by tracing the average fluorescence ratio (Fig. 9b) and measuring mitochondrial Ca^2+^ peak ratio (525 nm/458 nm) (Fig. 9c) following stimulation with 10 μM histamine, we found a partial rescue of mitochondrial Ca^2+^ load by PTPIP51 expression in Mul1-deficient neurons. Third, expressing PTPIP51 rescues mitochondrial fragmentation (Fig. 9d–e) and significantly suppresses Parkin translocation to mitochondria (Fig. 9g). Thus, our study consistently supports that enhancing ER-Mito contacts rescues Mul1-deficient mitochondrial phenotypes and thus suppresses Parkin-mediated mitophagy in neurons.

## Discussion

**Fig. 10:**
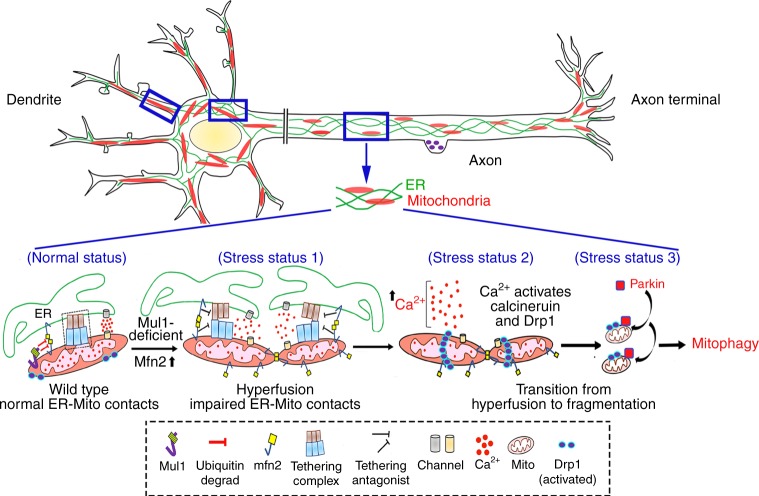
Mul1–Mfn2 pathway in the maintenance of mitochondrial morphology and ER-Mito contacts. Mul1 acts as a mitochondrial E3 ubiquitin ligase that binds and ubiquitinates Mfn2, leading to Mfn2 degradation, thus maintaining mitochondrial morphology (normal status). Mul1 deficiency results in the first phasic mitochondrial hyperfusion by increasing Mfn2 activity (stress status 1). Increased Mfn2 activity parallelly acts as an ER-Mito tethering antagonist, thus impairing ER-Mito coupling, disturbing mitochondrial bioenergetics and Ca^2+^ homeostasis, and reducing mitochondrial Ca^2+^ uptake from the ER. An increased cytoplasmic Ca^2+^ load activates calcineurin phosphatase, which activates and recruits Drp1 onto stressed mitochondria (stress status 2), thus triggering second phasic mitochondrial fragmentation. Those fragmented mitochondria loss their membrane potential and ATP production capacity, thus recruiting Parkin for mitophagy (stress status 3). By parallel maintenance of mitochondrial morphology and ER-Mito contacts, Mul1–Mfn2 pathway plays a unique role in maintaining and/or recovering stressed mitochondria in neurons. If this protection pathway fails, mitophagy is activated to eliminate damaged mitochondria

In the current study, we reveal Mul1 as an early checkpoint to protect neuronal mitochondria from rapid degradation under mild stress conditions, thus maintaining energy supply and ensuring cell survival. Mul1 deficiency leads to increased Mfn2 activity that plays two parallel roles: (1) inducing the first phasic mitochondrial hyperfusion; and (2) impairing ER-Mito interplay, the latter disturbs mitochondrial bioenergetics and Ca^2+^ homeostasis. Elevated cytoplasmic Ca^2+^ activates Drp1 through calcineurin, triggering the second phasic mitochondrial fragmentation and Parkin-mediated mitophagy. Consistently, stabilizing ER-Mito contacts by expressing anchoring protein PTPIP51 partially rescues Mul1-deficient phenotypes. Thus, impaired ER-Mito contacts and disturbed Ca^2+^ homeostasis link to mitochondrial fragmentation and mitophagy (Fig. 10). Our study provides new mechanistic insights into (1) why mitophagy in mature neurons is observed only in a small proportion of mitochondria following depolarization^5,7^ and occurs much more slowly than in non-neuronal cells^5,6^; and (2) why Parkin or PINK1 knock-out mice showed only subtle changes in mitochondrial morphology and neuronal degeneration^8,9,50,51^. This Mul1–Mfn2 pathway is particularly relevant to neurodegenerative diseases associated with chronic mitochondrial dysfunction and altered ER-Mito interplay^1,3,23,52^.

Maintaining a healthy pool of mitochondria in distal axons and dendrites is more protective than rapidly eliminating them through mitophagy. Our previous studies using mature cortical neurons demonstrated delayed Parkin-mediated mitophagy^5,6^, arguing for unique mechanisms that maintain and/or recover mitochondrial integrity before mitophagy activation. It was reported that Mul1 and Parkin mediate two parallel pathways of mitochondrial quality control in HeLa cells following acute depolarization using high-dose AA (40–80 μg/ml or 73–146 μM)^16^. Our study aimed to address whether Parkin-mediated mitophagy is the second resort for mitochondrial quality control after early protection mechanisms have failed. To address this issue, we instead applied mild stress conditions (100 nM AA), a ~400–800× lower dose of AA than widely used in the literature, in mature cortical neurons. We reasoned that this mild stress is more relevant to chronic mitochondrial dysfunction associated with aging-linked neurodegenerative diseases^6^. We established a Mul1-deficient neuronal system, where Parkin recruitment to fragmented mitochondria is robustly increased in response to mild mitochondrial stress. In contrast, overexpressing Mul1 suppresses Parkin translocation. Mul1 loss of function triggers a biphasic transition from mitochondrial hyperfusion at DIV10-11 to fragmentation at DIV14-15. Those fragmented mitochondria are associated with loss of membrane potential and ATP production and are subjected to mitophagic clearance. Since overexpressed Mul1ΔRing targets to mitochondria, we speculate that Mul1ΔRing competes with relatively low levels of endogenous Mul1 for its interaction or ubiquitination of Mfn2, thus interfering with Mito-ER contact and inducing mitochondrial fragmentation leading to mitophagy. Given that Parkin-mediated mitophagy is rapidly activated in HeLa cells upon mitochondrial depolarization, our study highlights a unique checkpoint for neuronal mitochondrial quality control. After this pathway has failed, Parkin is recruited to fragmentated mitochondria to initiate mitophagy. This is consistent as the loss of both Mul1 and *parkin* aggravates mitochondrial damage and induces neuronal degeneration^16^.

As a mitochondrial E3 ligase, Mul1 regulates Mfn levels in flies^16^ and both Mfn1 and Mfn2 levels in Hela cells^53^. However, it is not known which Mfn isoform is involved in Mul1 mutant phenotypes in neurons. Our current study provides multiple lines of analysis showing that Mfn2 but not Mfn1 mediates Mul1-deficient phenotypes. First, expressing Mfn2, but not Mfn1, in Mul1-deficient neurons accelerated mitochondrial fragmentation at the early stage (DIV10). Second, neurons expressing Mfn2 but not Mfn1 displayed a significant increase in Parkin translocation to stressed mitochondria at late stage (DIV14-15). Third, neurons expressing Mfn2 but not Mfn1 displayed enhanced mitophagy over a time course from DIV10-11 to DIV14-15. Fourth, mitochondria-targeted Mfn2 was increased in Mul1-deficient neurons. Fifth, Mul1-depleted cortical neurons displayed reduced Mfn2 ubiquitination and increased Mfn2 levels. In addition, expressing Mfn2 but not catalytically inactive mutant Mfn2 (K109A) induced an early transition of mitochondria into fragmentation at DIV10. Altogether, these results consistently support our notion that elevated Mfn2 activity is sufficient to mediate Mul1-deficient phenotypes in neurons.

Our study also reveals unique mitochondrial phenotypes in Mul1 mutant neurons: transient hyperfusion as an early response to maintain bioenergetic capacity. Prolonged stress impairs ER-Mito interplay and disturbs mitochondrial functions and Ca^2+^ homeostasis. Elevated cytoplasmic Ca^2+^ load activates Drp1 through calcineurin, thus triggering mitochondrial fragmentation. We provided six lines of evidence to support this mechanistic model: (1) mitochondrial association of Drp1 is increased at DIV13, a time point just before fragmentation occurs; (2) mitochondria are locked at the hyperfusion state when expressing catalytically inactive mutant Drp1K38A; (3) cytoplasmic Ca^2+^ load is elevated in Mul1-deficient neurons; (4) Drp1 is activated through the Ca^2+^/calcineurin when ER-Mito contacts are impaired; (5) expressing ER-Mito anchoring protein PTPIP51 rescues mitochondrial fragmentation. We further confirmed this model by applying two blockers of calcineurin activity, which arrest mitochondria in the hyperfusion state in Mul1-depleted neurons. Thus, our study reveals a new mechanistic pathway that links impaired ER-Mito contacts to Drp1-dependent mitochondrial fragmentation, a process necessary for Parkin-mediated mitophagy.

Transient mitochondrial hyperfusion was proposed as an adaptive response to maintain bioenergetic status under various stress conditions^54,55^, and was also observed in cells exposed to starvation^56^, hypoxia^57^, cold stress^58^, and toxic insults^59^. It will be interesting to investigate into the role of Mul1–Mfn2 pathway in the maintenance and recovering of neuronal mitochondrial integrity in response to these conditions. Post-translational modifications, including phosphorylation and SUMOylation, regulate Drp1 activity under various stress conditions. In non-neuronal cells, Mul1 SUMOylates Drp1 in response to severe apoptosis induction and thus stabilizes Drp1 on mitochondria^17,18^. However, in Mul1-deficient neurons without exogenous insult, we did not observe any significant change in mitochondrial SUMOylation levels.

Mfn2 is involved in multiple pathways in addition to mitochondrial fusion^60^. Overexpressing Mfn2 induces mitochondrial dysfunction and cell death^61,62^. Specifically, Mfn2 is involved in the regulation of ER-Mito contacts in MEFs and HeLa cells^63^. Mfn2 ablation in MEFs increases the distance between the ER and mitochondria and reduces mitochondrial Ca^2+^ uptake^64^. It was also reported that Mfn2 is required for mitochondrial Ca^2+^ uptake in skeletal muscle^65^ and cardiomyocytes^66^. This model was recently challenged by several studies. Using quantitative EM analysis, one study reported an increase, not a decrease, in the close contacts between ER and mitochondria in *Mfn2*^*−/−*^ cells^19^. In a second study, cells with deleted or reduced Mfn2 display increased ER-Mito tethering, enhanced Ca^2+^ transfer from ER to mitochondria, and increased sensitivity to apoptotic stimuli by mitochondrial Ca^2+^ overload^20^. These ultrastructural and functional observations suggest an alternative model in which Mfn2 negatively modulates ER-Mito contacts, probably through its role as a tethering antagonist^20,67,68^. Such a role is likely mediated by lateral interference of increased Mfn2 with other linker components in the same membrane^22^; this tethering antagonist may be more robust in neuronal ER-Mito contacts. Our current study in mature cortical neurons shows a negative role of Mfn2 in maintaining ER-Mito contacts. Using STED nanoscopy and iTEM ultrastructural analysis combined with mitochondrial Ca^2+^ uptake assay, our study consistently supports a role of Mul1 in sustaining ER-Mito contacts by reducing Mfn2 levels. Enhancing ER-Mito contacts by expressing an ER-Mito anchoring protein in Mul1-deficient neurons rescues mitochondrial fragmentation and suppresses Parkin-mediated mitophagy. Thus, maintaining ER-Mito contacts in mature neurons is critical to suppressing mitochondrial fragmentation and mitophagy. This notion is consistent with a recent report showing that impairing ER-Mito contacts increases Parkin-mediated mitophagy upon acute mitochondrial depolarization^30^. Parkin-mediated mitophagy is significantly delayed in mature primary neurons in response to mild mitochondrial stress^6^, a condition more relevant to pathological stress in aging-associated neurodegenerative diseases. Therefore, mitochondria-targeted Mul1 ideally acts as an early checkpoint via restraining Mfn2 levels and thus sustaining ER-Mito contacts. After this Mul1–Mfn2 pathway has failed, Parkin is recruited to mitochondria as the second resort for mitochondrial quality control. We further demonstrate that elevated Mfn2 activity impairs this protection mechanism, thus leading to rapid mitochondrial dysfunction and fragmentation. Our findings are supported by a recent study showing that altered Mfn2 activity has deleterious effects on neuronal mitochondrial function and is associated with neuropathy in Charcot-Marie-Tooth disease type 2A (CMT2A)^69^.

Several critical roles of mitochondria, including lipid and energy metabolism and Ca^2+^ homeostasis, rely on their interplay with the ER. Altered ER-Mito coupling has been reported in the pathogenesis of several major neurodegenerative diseases^23,70^. Although affected neurons in AD, PD, ALS/FTD, and HSP display various pathological changes, they all share common features: chronic mitochondrial dysfunction, altered ER-Mito interactions with impaired Ca^2+^ homeostasis and energy metabolism, and mitochondrial fragmentation^23^. Our study in mature cortical neurons reveals that sustaining ER-Mito contacts is critical to maintaining mitochondrial integrity, suppressing mitochondrial fragmentation and mitophagy, thus providing a mechanistic explanation for the seemingly disparate features of these neurodegenerative diseases.

## Methods

### Antibodies and DNA constructs

Sources of antibodies or reagents are as follows: antibodies against MAP2 (rabbit; AB5622; Millipore), p62/SQSTM1 (rabbit; PM045; MBL), Mul1 (rabbit; HPA017681; Sigma-Aldrich), Hsp60 (rabbit; 4870S; Cell Signaling), Lamp1 (rat; 1D4B; DSHB), HA (mouse; mms-101p; Covance), Mfn2 (rabbit; M6319; Sigma-Aldrich), Drp1 (rabbit; 8570; D6C7; Cell Signaling), Ser637-Drp1 (rabbit; 4867; Cell Signaling), mCherry (mouse; ab125096; abcam), Flag (mouse; F1804; Sigma-Aldrich, and rabbit; F7425; Sigma-Aldrich), GFP (rabbit; ab6556; abcam), TOM20 (rabbit; 11415; Santa Cruz), Ubiquitin (mouse, sc 8017; Santa Cruz), Myc (chicken; ab172; abcam), cytochrome c (mouse; 556432; BD Biosciences), Alexa Fluor 488- or 546- or 594- or 633-conjugated secondary antibodies (Invitrogen), Mouse IgG- or Rabbit IgG-HRP (GE HealthCare), and Nanogold (2004; Nanoprobe). The constructs GFP-Mul1, Flag-Mul1, GFP-Mul1-H319A, and Mul1 shRNA were previously described^16^. Flag-tagged [Mul1∆Ring, Mul1∆(TM1/TM2)] constructs were provided by C.A. Joazeiro (Scripps Inst., USA). pAc-GFPC1-Sec61beta, pCDNA3.1-Drp1(K38A), pcDNA-4mtD3cpv, pCDA-D3cpv, and Myc-tagged Mfn2(K109A) were purchased from Addgene Inc (Cambridge, MA). Construct for the ratiometric ATP sensor GO-ATeam2 was kindly provided by Hiromi Imamura (Kyoto University, Japan); Myc-tagged (Mfn1, Mfn2) constructs were provided by D. Chan (Caltech Inst., USA); Mito-DsRed and m-Cherry Parkin constructs were provided by R. Youle (NINDS, NIH, USA). Mito-CFP was provided by D. C. Rubinstein (Cambridge University, UK). HA-PTPIP51 was provided by Christopher C.J. Miller (King’s College, UK). Mt-Keima was purchased from MBL (#AM-V0251HM) and verified by DNA sequencing. Mouse Mfn2 shRNA constructs were purchased from OriGene. The Mfn2 shRNA sequences are 5′-TCACCGTCAAGAAGGATAAGCGACACATG-3′ and 5′-AGCAGAGCCAAACTGCTCAGGAATAAAGC-3′.

### Cortical neuron culture and transfection

Cortices were dissected from E18-19 mouse embryos using the papain method. Cortical neurons were plated at a density of 0.5 million per 10 cm^2^ onto a 5-day-old glial feeder layer sitting on polyornithine (P4957; Sigma Aldrich) and matrigel (356234; BD Biosciences) coated coverslips. Neurons were grown overnight in plating medium with 5% FBS, insulin (30 µg/ml), GlutaMAX (35050-61, Thermo Fisher), and B27 (17504-044; Thermo Fisher) in Neurobasal medium (21103-049; Thermo Fisher). Starting at DIV2, cultures were maintained in conditioned medium with half-feed changes of neuronal feeding media every 3 days. Unless otherwise noted, neurons were transfected at DIV7-8 using the modified Ca^2+^ phosphate method and imaged at DIV10-11 or DIV14-15. Animal care and use were carried out in accordance with all relevant ethical regulations and NIH guidelines for animal testing and research. The study received ethical approval by the NIH, NINDS/NIDCD Animal Care and Use Committee.

### Lentivirus production and infection

HEK293T cells were transfected with the vector, psPAX2, and pMD2G at a 4:3:1 ratio to produce the lentivirus expressing scramble or Mul1 shRNA. After a 24-hr transfection, the medium was replaced with 7 ml of DMEM + 10% FBS. Virus-containing media were collected at 48 and 72 h post transfection and then centrifuged at 1600 rpm for 5 min to remove cell debris. The pre-cleaned supernatant was collected and ultra-centrifuged at 90,000 × *g* for 1.5 h. The supernatant was carefully removed, and PBS was added to resuspend the viral pellets. The concentrated virus was aliquoted and stored at −80 °C until use. 1 × 10^7^ of freshly dissociated cortical neurons were infected with a concentrated lentivirus.

### Immunocytochemistry

Neurons were fixed at DIV10-11 or DIV14-15 with 4% paraformaldehyde (15710, Electron Microscopy System) and 4% sucrose in 1× phosphate-buffered saline (PBS) at 4 °C for 30 min or with 100% ice-cold methanol at −20 °C for 10 min, followed by a 3-time wash with PBS for 5 min each, and incubated in PBS-based buffer containing 0.2% saponin (S4521; Sigma-Aldrich), 5% goat serum, and 2% BSA for 1 h. Fixed cultures were incubated with primary antibodies in PBS-based buffer containing 1% BSA and 0.05% saponin at 4 °C overnight. Samples were washed four times with PBS at room temperature (RT) for 5 min each, incubated with secondary fluorescent antibodies for 1 h at RT, re-washed with PBS, and then mounted with Fluoro-Gel anti-fade mounting medium (17985-10; Electron Microscopy System) for imaging.

### Measurement of mitochondria morphology

Mitochondria were imaged after co-expressing Mito-DsRed with Mul1 or Mul1 mutants using an Olympus confocal microscope with an oil-immersion ×63 objective/(NA-1.45). Unless otherwise noted, images were sequentially acquired below saturation from top to bottom with Z-step (0.37 µm) at a resolution of 1024 × 1024 pixels, 12 bit, and ×2 optical zoom. For mitochondrial size quantification, images were imported into ImageJ (NIH), converted to 8 bit, stacked using Z-projection (maximum intensity), and the background was removed using the ImageJ built-in plug-in “remove background”. The images were thresholded, despeckled, and analyzed using the ImageJ (NIH) built-in plug-in “shape descriptor”. The data were collected and transferred to Excel for analysis.

### Mitochondria depolarization assay

Cortical neurons were treated for 3 h with DMSO or 100 nM antimycin-A (AA) (A8674; Sigma-Aldrich) in combination with lysosomal inhibitors [LI; 10 µM E64D (330005; EMD Millipore) and 10 µM pepstatin A (El10, EMD Millipore)].

### Inhibition of calcineurin activity

Cortical neurons were treated for 48 h with DMSO or 0.5 μM cyclosporine A (CsA) (1101; Tocris Bioscience) or 0.6 μM FK506 (F4679; Sigma-Aldrich).

### Seahorse analysis

Oxygen consumption rate (OCR) was measured using a Seahorse XFe96 Flux Analyzer (Agilent Technologies). Briefly, cortical neurons were seeded in pentaplicate at 8 × 10^6^ cells/well in an XF96 culture microplate and infected with scr-shRNA or Mul1-shRNA. Assays were initiated by removing the growth medium form each well and replacing it with 180 μl of assay medium (5 M NaCl, 2 M KCl, 2 M CaCl_2_, 1 M KH_2_PO_4_, 2 M MgCl_2_, 1 M HEPES, 2.5 m glucose, pH 7.4) prewarmed to 37 °C. The cells were incubated at 37 °C for 45 min to allow for media temperature and pH to reach equilibrium before the first-rate measurement. OCR measurements of mitochondrial function was obtained by sequential injections of 1 μM oligomycin, 1 μM FCCP, and 0.5 μM rotenone/AA. All OCR measurements were normalized to non-mitochondrial respiration and the final values normalized to total cell number plated in each well as live cell numbers were not different between each group.

### Measurement of mitochondria ∆ψ_m_

Neurons in conditioned growth media were incubated at 37 °C with TMRE (50 nM) (T669; Thermo Fisher) for 20 min in a CO_2_ incubator (Thermo Scientific). Post-treatment, neurons were transferred to imaging media containing (in mM) 20 HEPES, 2 C_3_H_3_NaO_3_, 2 GlutaMAX, with 2% B27 and 0.6% glucose in HBSS with Ca^2+^ and Mg^2+^ (14025-092; Thermo Fisher), washed 3 times with imaging media, and mounted for live-cell imaging. Confocal images were acquired within 30 min using an Olympus confocal oil-immersion ×63 (1.45 NA) objective with image acquisition settings set below saturation. Five to six optical sections for each image were taken from top-to-bottom of the specimen. Measurement of TMRE integrated intensity was done using Image J and expressed as corrected total cell fluorescence (CTCF) using the formula (CTCF = Integrated mean intensity - area mean background values). The mean fluorescent intensity of TMRE in the soma for each transfected group was normalized as a percentile ratio relative to that in neurons expressing the GFP construct.

### ATP measurement

A FRET-based fluorescent ATP probe (GO-ATeam2) was used to measure intracellular cytosolic ATP levels^38^. Briefly, the probe encodes FRET pair fluorescent proteins, green fluorescent protein (GFP), and orange fluorescent protein (OFP). Transfected neurons were visualized with an Olympus Fluoview (F1000) inverted microscope (Olympus Corp., USA) using the Plan Fluor ×63 (1.45 NA) oil-immersion objective lens (Olympus). The filters used for dual-emission ratio imaging of GO-ATeam were purchased from Semrock (Rochester, NY) and included an FF01-482/18 excitation filter, an FF495-DiO2 dichroic mirror, and two emission filters (FF01-520/35 for GFP and FF01-562/40 for OFP). The images were imported to ImageJ (NIH); CTCF values for each channel (GFP and OFP) were quantified and the ratio (OFP/GFP) was calculated using Microsoft Excel software.

### Immuno-electron microscopy

Wild-type or Mul1-deficient neurons expressing a GFP-tagged ER marker (Sec61ß) were fixed at DIV10-11 in a PBS-based mix of 4% paraformaldehyde and 0.1% glutaraldehyde (16020; Electron Microscopy System) for 30 min at RT. The samples were washed with PBS four times, blocked and permeabilized with a PBS-based mix of 5% normal goat serum and 0.1% saponin for 1 h at RT. Samples were then incubated with a primary antibody [anti-GFP (1:500)] in a PBS-based buffer containing 5% normal goat serum and 0.05% saponin for 1 hr at RT, washed with PBS four times, and incubated with nanogold Fab’ conjugates (1:200 dilution) (Nanoprobe) made in PBS containing 5% normal goat serum and 0.05% saponin for another hour at RT. Samples were then washed with PBS, fixed with 2% glutaraldehyde in PBS for 30 min, silver enhanced with 0.2% OsO_4_ in 0.1 M phosphate buffer for 30 min, and en bloc mordanted with uranyl acetate. The samples were then dehydrated in a series of graded ethanol washes and embedded in epoxy resins. Thin sections were stained with uranyl acetate and lead citrate (EM Facility, National Institute of Neurological Disorders and Stroke, National Institutes of Health). Sections were visualized under a JEOL (Akshima, Japan) 1200 EX transmission electron microscope (TEM) and digital images were captured with a CCD camera system (XR-100; Advanced Microscopy Techniques, Danver, MA). The TEM images were analyzed using ImageJ.

### Super-resolution 3D-STED imaging

Dual-color 3D-STED imaging was performed by using Leica TCS SP8 STED 3X, Germany. For fixed-cell imaging, the fluorophores were excited with a tunable white light laser (70% of maximum power). The images were captured using two HyD detectors and an oil objective (HC PLAPO 100X/1.40 OIL). For imaging GFP, cells were excited at 488 nm (25%) with the STED depletion laser at 592 nm (15%); its emission fluorescence at 495–584 nm was collected using time-gated HyD detectors (1.25–6.5 ns). Alexa 594 were excited at 590 nm (1.25%) with the STED depletion laser at 660 nm (10%); its emission fluorescence between 594–660 nm was collected using time-gated HyD detectors (1.25–6.5 ns). For dual-color imaging of Alexa Fluor 594 and GFP 488, Alexa Fluor 594 was first imaged to avoid bleaching by the 592 nm STED depletion laser in the frame-scanning mode. Image acquisition and the microscope platform were controlled by built-in Leica Application Suite (LASX) software. For image acquisition, settings were kept below saturation at 1028 × 1028 pixels, 8 bit, with a (×5) optical zoom for capturing the soma-dendritic region and a (×6) optical zoom for capturing dendrites. Images were acquired sequentially from top to bottom using system-optimized Z-step setting and then were deconvolved using a Leica built-in Huygens STED deconvolution software.

### Preparation of samples for STED

A modified fixation protocol was used to improve an ER fixation procedure for STED. Mouse cortical neurons were fixed post-transfection at DIV10-11 by briefly rinsing in a 2% sucrose solution in PBS, followed by dipping in tetrahydrofuran and being kept on dry ice overnight. The next day, the coverslips were placed for fixation in a solution of 10 ml methanol and 1 ml 16% paraformaldehyde and kept at −20 °C for 3 h. Coverslips were transferred post-fixation to RT for 1 h. Samples were rehydrated in graded concentrations of ethanol in PBS, starting with 100%–95%–70%–50%, for 10 min each. Post-fixation, cells were immunostained for mitochondria using an antibody against Tom20 (1:100) overnight at 4 °C, followed by treatment with a 594-conjugated secondary antibody (1:50) for 1 h at RT. High-resolution images for each transfected group were captured using Leica TCS SP8 STED 3X. Meander’s colocalization analysis between mitochondria and ER was done using a built-in colocalization plug-in in Velocity Software. The data were transferred to Excel for analysis.

### Ubiquitination assay

Cortical neurons infected with scramble or Mu1 shRNA were treated with 10 uM MG132 (474790; Calbiochem) for 4 h. Cells were then lysed in RIPA lysis buffer (50 mM Tris, pH 7.5, 150 mM NaCl, 1% Triton X-100, 0.1% SDS, and 1% sodium deoxycholate) supplemented with protease inhibitor. The lysate was then incubated with control rabbit IgG (2729S; Cell Signaling) or anti-Mfn2 antibody (9842; Cell Signaling) at 4 °C overnight, and then incubated with protein A-Sepharose (17-0780-01; GE-Healthcare). The recovered immunocomplex was extensively washed with lysis buffer four times and eluted with reducing LDS sample buffer. Samples were resolved with 4–12% NuPAGE followed by immunoblotting with mouse anti-Ub (sc-8017; Santa Cruz), and rabbit anti-Mfn2 (11925; Cell Signaling).

### Isolation of mitochondrial fraction

Cortical neurons infected with scramble or Mul1 shRNA were washed 2 times with PBS and scrapped over ice with mitochondrial isolation buffer (220 mM mannitol, 80 mM KCl, 0.5 mM EGTA, 20 mM HEPES, 68 mM sucrose, 2 mM MgCl_2_, 20 mM N-ethyl maleimide and protease inhibitors). Cell suspension were broken by passing through a 26-gauge needle. Samples were then centrifuged at 800 × *g* at 4 °C for 10 min to pellet the nuclear fraction. The supernatants were collected and centrifuged at 800 × *g* at 4 °C for 5 min to completely remove the nuclear fraction. Post-nuclear supernatants were then centrifuged at 9000 × *g* at 4 °C for 20 min to pellet the mitochondrial fraction. The mitochondrial pellet was then dissolved in RIPA buffer. The protein content was normalized to the mitochondrial fraction and processed for PAGE and western blotting.

### Immunoblotting

Human embryonic kidney cells (HEK293T) were cultured in 35-mm plates at a density of ~1 × 10^6^ and constructs were transfected using Lipofectamine 2000 (11668019, Thermo Fisher). For immunoblotting, samples were harvested and suspended in ice-cold RIPA lysis Buffer [150 mM NaCl, 0.1% Triton X-100, 0.5% sodium deoxycholate, 0.1% SDS, 50 mM Tris-HCl pH 8.0, protease inhibitor (P8430; Sigma Aldrich) and phosphatase inhibitor cocktail (4906845001; Sigma Aldrich). The lysates were centrifuged at 13,000 × *g* for 15 min at 4 °C; pellets were discarded; and the supernatant was assayed for protein concentration. The protein (5 μg) was resolved on 4–12% Bis-Tris gels (12313623; Invitrogen) followed by Western blots on the same membranes after stripping between each application of the antibody.

### Ca^2+^ measurements

Two FRET-based ratiometric fluorescent Ca^2+^ probes were used to measure Ca^2+^: pCDNA-D3cpv that targets the cytoplasm and pcDNA-4mtD3cpv that targets the mitochondrial matrix^44^. Briefly, the probe encodes FRET pair fluorescent proteins, an enhanced cyan fluorescent protein (CFP), and circularly permuted Venus 173. The probe was transfected in primary neuron cells by the Ca^2+^ phosphate method at DIV7-8 and imaged at DIV10-11. Samples were treated with 10 μM Histamine (H7125; Sigma-Aldrich) for releasing Ca^2+^ from the ER. Samples were visualized with an Olympus Fluoview (F1000) inverted microscope (Olympus Corp., USA) using the Plan Fluor 63x/(1.45 NA), oil-immersion objective lens (Olympus). Filters used for dual-emission ratio imaging of pCDNA-D3cpv and pcDNA-4mtD3cpv were purchased from Semrock (Rochester, NY). Images were imported to ImageJ.

### Measurement of mitophagy with mt-Keima

For cortical neurons co-transfected with mt-Keima and other different constructs, live-cell imaging was performed in Low Fluorescence Hibernate A media (BrainBits) with 0.5 mM Glutamax and B27, and maintained in an environmental chamber where the temperature was kept at 37 °C. Neurons were visualized with a Zeiss LSM 880 oil immersion 63x objective with two sequential excitation lasers at 458 nm (green, healthy mitochondria at neutral pH) and 561 (red, damaged mitochondria engulfed by lysosomes under acidic pH) and an emission spectrum from 590–680 nm. Images were sequentially acquired from top to bottom with Z-step (1 μm) at a resolution of 1024 × 1024 pixels, 8 bit. Images were imported into ImageJ (NIH), stacked using Z-projection (maximum intensity), and the background was removed using the built-in plugin “remove background”. With post-processed images, mitophagy index was defined as the ratio of the area of lysosomal signal (red)/mitochondrial signal (green) in the cell body, and were normalized to control conditions.

### Quantification and statistical analysis

All quantifications were performed unblinded. Statistical parameters including the definitions and exact value of n (e.g., total number of experiments, replications, axons, organelles, or neurons), deviations, p values, and the types of the statistical tests are reported in the figures and corresponding figure legends. Statistical analysis was carried out using Prism 7 (GraphPad Software). Statistical analysis was conducted on data from three or more biologically independent experimental replicates. Comparisons between groups were planned before statistical testing and target effect sizes were not predetermined. Error bars displayed on graphs represent the mean ± SEM of at least three independent experiments. Statistical significance was analyzed using unpaired Student’s *t*-test for two groups or Ordinary one-way ANOVA with Dunnett multiple comparison test for multiple groups. *p < 0.05, **p < 0.01, and ***p < 0.001 were considered significant.

### Reporting summary

Further information on research design is available in the Nature Research Reporting Summary linked to this article.

## Supplementary information

Supplementary Information

Peer Review File

Reporting Summary

Description of Additional Supplementary Files

Supplementary Movie 1

Supplementary Movie 2

Supplementary Movie 3

## Data Availability

The data sets generated and/or analyzed during the current study are available from the corresponding author on request.
