# Supplementary material for: Mul1 restrains Parkin-mediated mitophagy in mature neurons by maintaining ER-mitochondrial contacts
Source: Nat Commun. 2019 Aug 13;10:3645. doi: 10.1038/s41467-019-11636-5 (PMC6692330; doi:10.1038/s41467-019-11636-5)
Supplement: Supplementary file 1 — Supplementary Information [file 41467_2019_11636_MOESM1_ESM.pdf]

Mul1 restrains parkin-mediated mitophagy in mature neurons by maintaining ER-mitochondrial contacts

Rajat Puri, Xiu-Tang Cheng, Mei-Yao Lin, Ning Huang, and Zu-Hang Sheng*

Supplemental Information

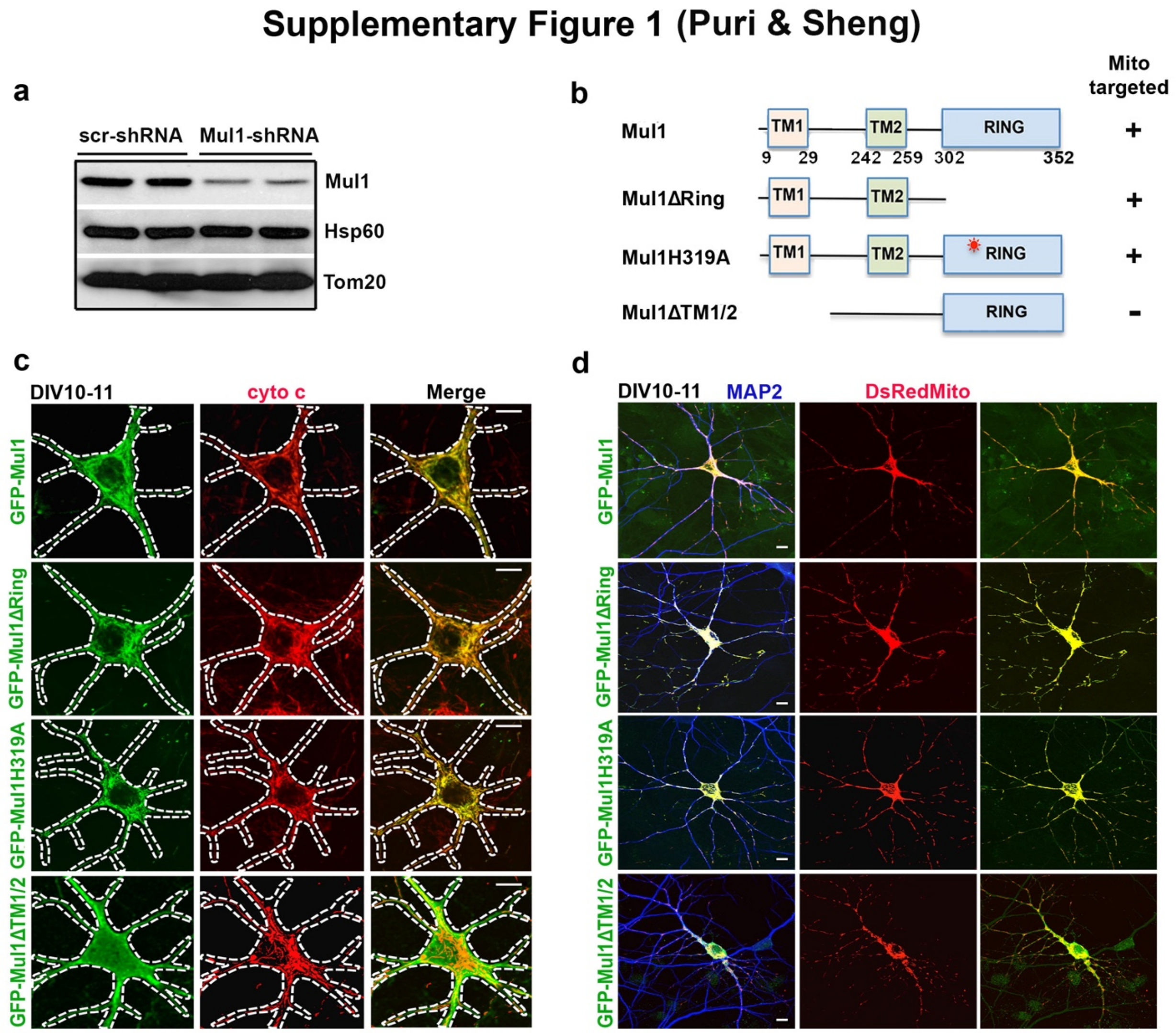

Supplementary Fig. 1 Mitochondrial targeting of wild-type and mutant Mul1 in neurons. **a** Representative immunoblots showing the selective reduction of Mul1, but not mitochondrial proteins Hsp60 and Tom20, by Mul1-shRNA. **b** Schematic diagram showing Mul1 structural organization: two mitochondrial trans-membrane segments (TM1, TM2); a RING finger domain with E3 ubiquitin ligase activity at the C-terminus; Mul1 loss-of-function mutant with the RING domain deletion (Mul1ΔRing); a single residue mutation in the RING domain (Mul1-H319A); and a truncated Mul1 mutant with both TM1/2 deletion (Mul1ΔTM1/2) as an off mitochondrial-targeting control. **c, d** Representative images showing mitochondria-targeting of GFP-tagged Mul1, Mul1ΔRing, and Mul1-H319A, and a diffused pattern of Mul1ΔTM1/2 in cortical neurons. Somato-dendritic regions of transfected neurons were traced by dashed lines. Scale bars: 10 μm.

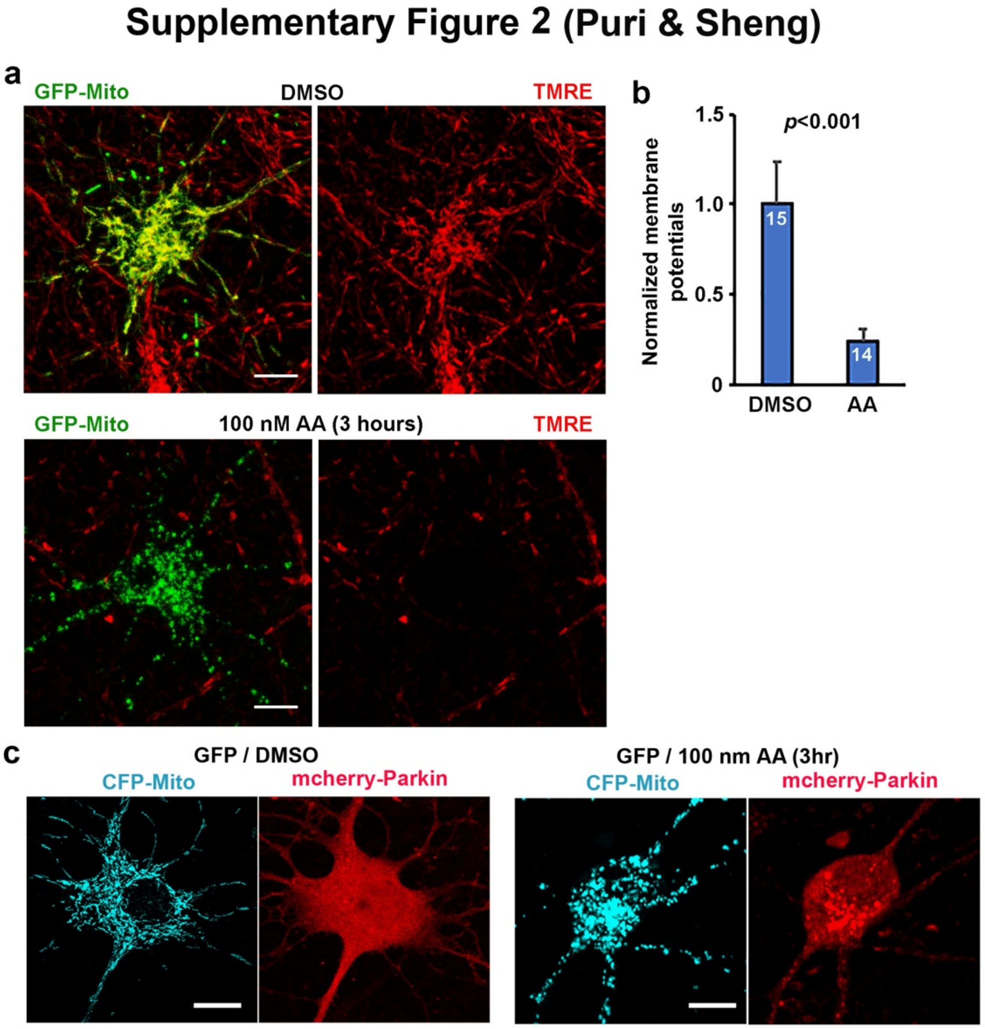

Supplementary Fig. 2 Mild AA treatment is sufficient to depolarize mitochondria and induce Parkin translocation. **a, b** Representative images and quantitative analysis showing mitochondrial depolarization in neurons following a 3-hour treatment with 100 nM AA. Mouse cortical neurons were transfected with Mito-GFP at DIV7, followed by treatment of 100 nM AA for 3 hours and TMRE (25 nM) staining for 20 mins before live-cell imaging at DIV10-11. Images were acquired by the same setting. Note a significant reduction in mitochondrial membrane potential in the AA-treated neurons relative to DMSO control. **c** Representative images showing Parkin translocation in control neurons expressing GFP under mild stress. Cortical neurons at DIV8 were co-transfected with mCherry-Parkin and CFP-Mito together with GFP, followed by incubation at DIV10 with 100 nM AA or DMSO for 3 hours before fixation and imaging. Note that a small portion (14.84 ± 1.56%) of control neurons display Parkin translocation in response to mild stress (see **Fig. 1e**). Data were analyzed from the total number of neurons indicated in the bars and expressed as mean ± s.e.m. Mann Whitney test. Scale bars: 10 μm.

Supplementary Figure 3 (Puri & Sheng)

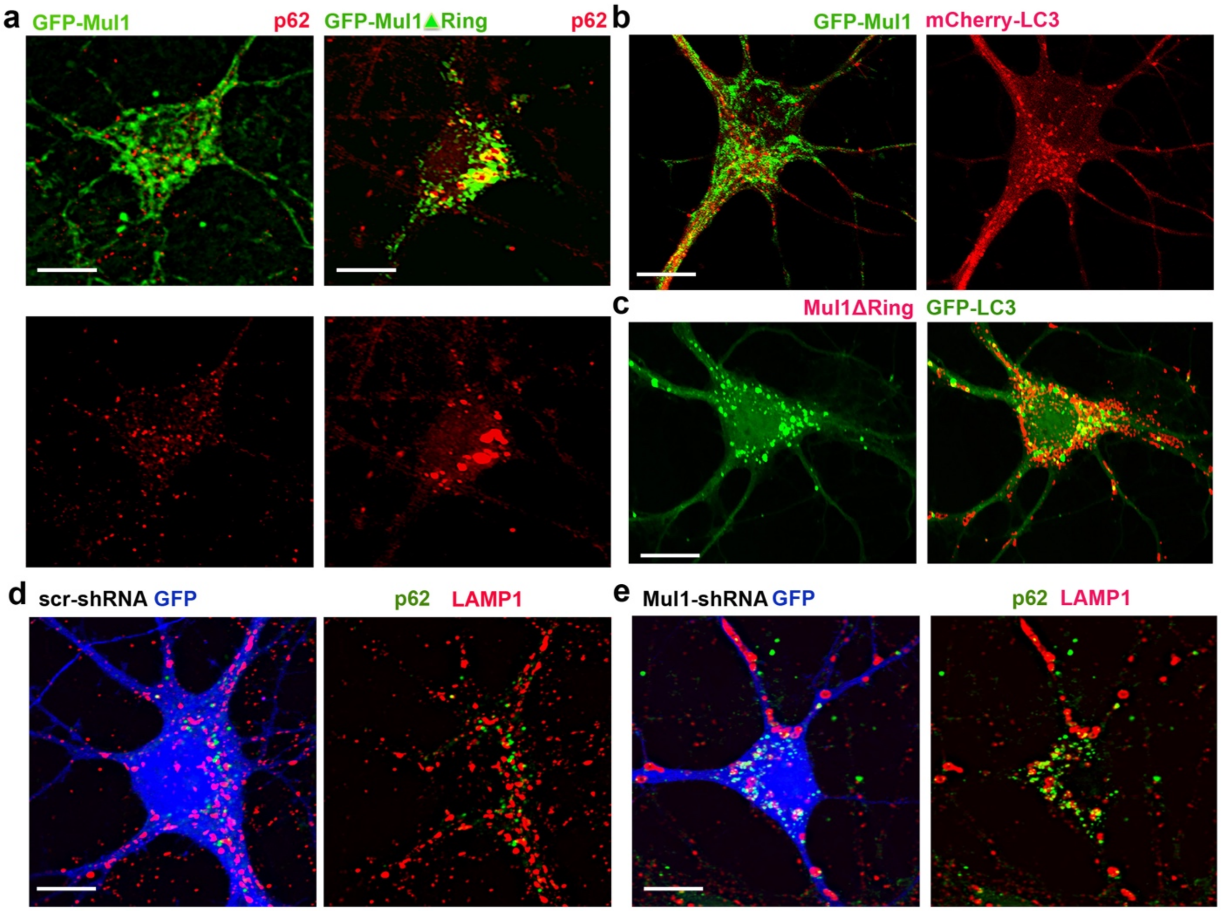

Supplementary Fig. 3 Mul1 deficiency triggers mitophagy in neurons. **a-c** Representative images showing clustering of p62 (**a**) or LC3-II (**b, c**) around fragmented mitochondria in Mul1-deficient neurons at DIV14-15. Cortical neurons were co-transfected at DIV7-8 with GFP-Mul1 or GFP-Mul1ΔRing or together with mCherry-LC3 or GFP-LC3, followed by immunostaining with p62 and imaging at DIV14-15. Mitochondria were labeled by Mul1 or its mutants. **d, e** Representative images showing clustering of endo-lysosomes around p62-labeled autophagic organelles in neurons with Mul1 depletion. Cortical neurons were co-transfected at DIV7-8 with scr-shRNA + GFP (**d**) or Mul1-shRNA + GFP (**e**), followed by co-immunostaining with p62 and LAMP1 at DIV14-15. GFP signal (pseudo blue) was used to trace transfected neurons. Scale Bars: 10 μm.

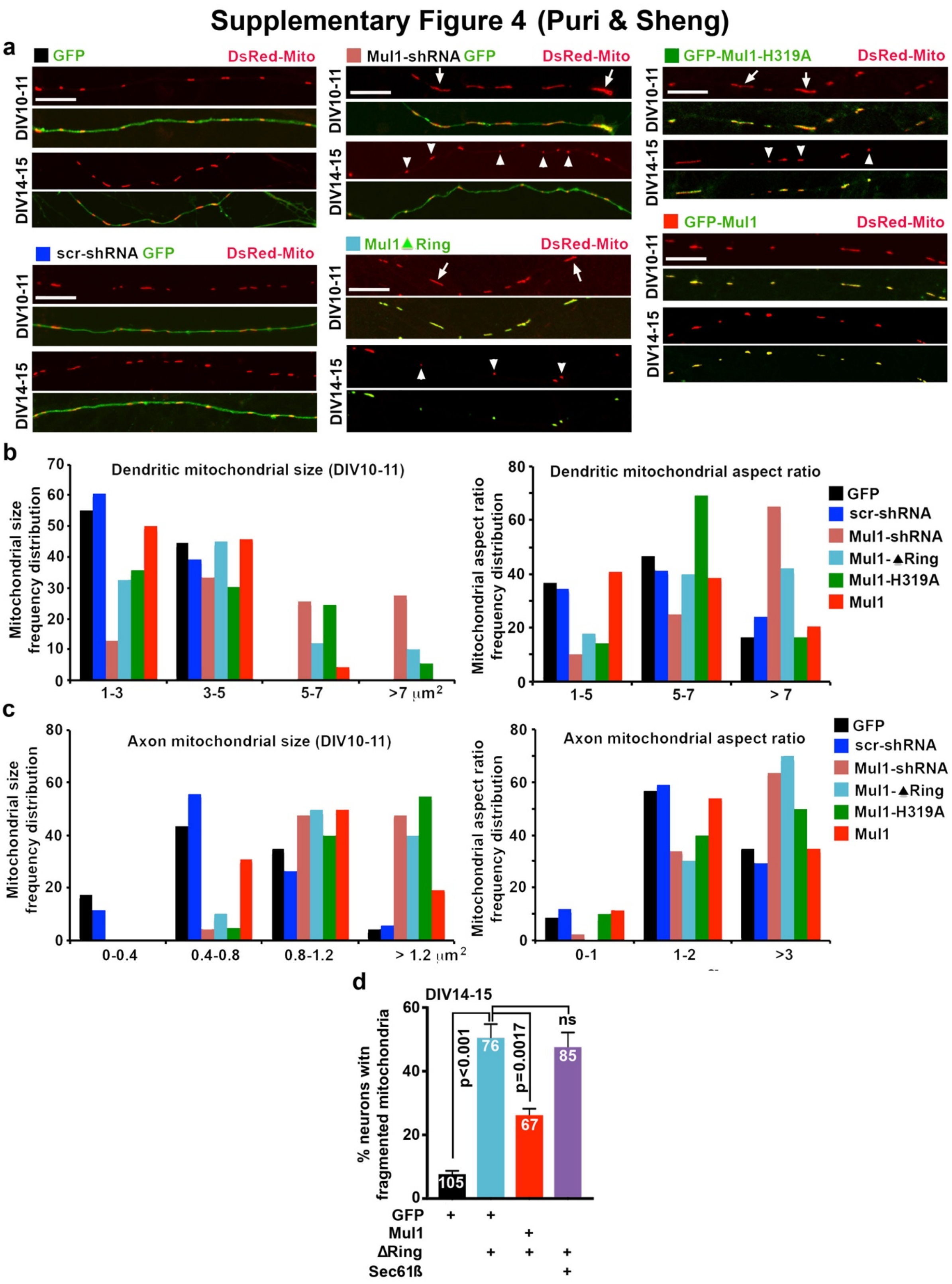

Supplementary Fig. 4. Mul1-deficient neurons display transient mitochondrial hyperfusion to

fragmentation. **a** Representative axon images showing a biphasic transition of mitochondria from hyperfusion to fragmentation in Mul1-deficient cortical neurons. Neurons at DIV7-8 were co-transfected with DsRed-Mito and GFP; or together with scr-shRNA, Mul1-shRNA, Flag-Mul1ΔRing, GFP-Mul1-H319A, or GFP-Mul1. Neurons were immunostained with MAP2 to label dendrites and DsRed-Mito was imaged along MAP2-negative axons at both DIV10-11 and DIV14-15. Note that hyperfused tubular mitochondria in axons (arrows) at DIV10-11 and fragmented ones (arrow heads) at DIV14-15 were readily observed in distal axons of neurons transfected with Mul1-shRNA, Mul1ΔRing, or Mul1-H319A. **b, c** Frequency distributions of average mitochondrial size and aspect ratio in dendrites (**b**) and axons (**c**). Note that long tubular mitochondria were more frequently observed in cortical neurons transfected with Mul1-shRNA, Mul1ΔRing, or Mul1-H319A compared to neurons expressing GFP, scr-shRNA, or Mul1. **d** Overexpressing Mul1 rescues mitochondrial fragmentation in Mul1-deficient neurons. Cortical neurons at DIV7-8 were co-transfected with DsRed-Mito and Flag-Mul1ΔRing or co-transfected with both Mul1ΔRing and Mul1 or GFP-Sec61-β; mitochondria were imaged at DIV14-15. Data were analyzed from the total number of neurons indicated in the bars in three experiments (**d**). The total number of neurons for dendritic mitochondria groups (**b**): GFP (55), scr-shRNA (58), Mul1-shRNA (36), Mul1Δ Ring (58), Mul1-H319A (41), and Mul1 (62); and for axonal mitochondria groups (**c**): GFP (23), scr-shRNA (34), Mul1-shRNA (44), Mul1ΔRing (20), Mul1-H319A (20), and Mul1 (26). Data are expressed as mean ± s.e.m. Ordinary one-way ANOVA with Dunnett multiple comparison test was used for comparing multiple groups. Scale bars: 5 μm. (**Also see Fig. 2**).

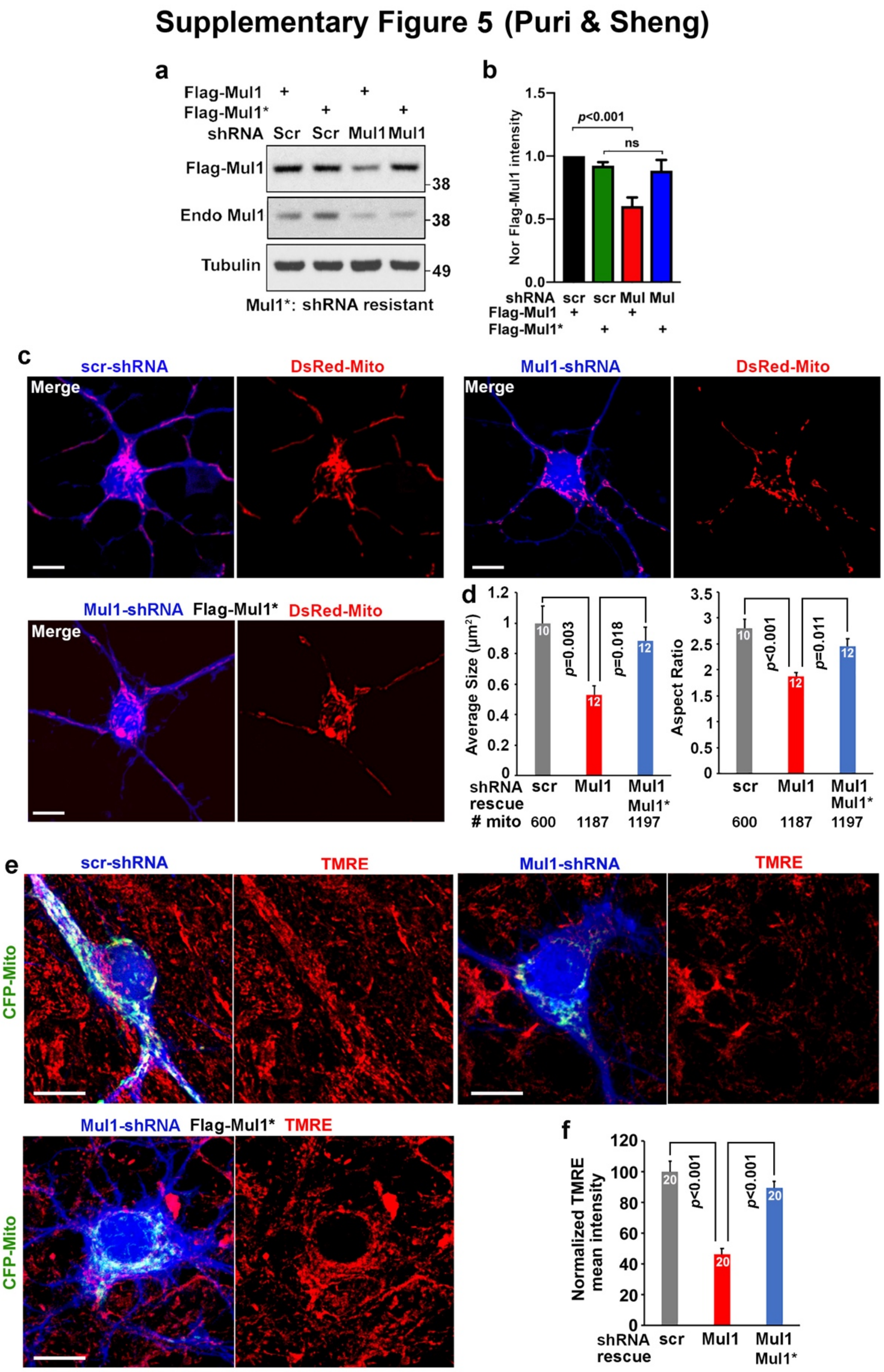

Supplementary Fig. 5 Expressing shRNA-resistant Mul1* rescues mitochondrial fragmentation and depolarization. **a, b** Characterization of shRNA-resistant Mul1*. Mul1* was generated by

substituting eight third-code nucleotides in Mul1-shRNA-targeting sequence (A210T, T213A, G216A, G219A, T222A, T225A, A228G, and A231G) without changing the amino acid. HEK293T cells were co-transfected with Flag-Mul1 or Flag-Mul1* and scr-shRNA or Mul1-shRNA as indicated, followed by immunoblotting 3 days after transfection using antibodies against Mul1 and α-tubulin. Equal amounts (20 μg) of cell lysates were loaded. The intensities of Flag-Mul1 were calibrated with α-tubulin levels and normalized to the group co-expressing Flag-Mul1 and scr-shRNA. While Mul1-shRNA suppresses endogenous Mul1 and significantly deplete exogenous Flag-Mul1 ($p<0.001$), it fails to suppress Mul1* ($p=0.68$). Flag-Mul1 and endogenous Mul1 are blotted separately to avoid signal saturation of overexpressed Flag-Mul1. Data were from three independent experiments, unpaired *Student's* t test. **c-f** Representative images and quantitative analysis showing rescued mitochondrial fragmentation (**c, d**) and depolarization (**e, f**) in Mul1-depleting neurons by co-expressing Mul1*. Cortical neurons were infected at DIV7-8 with DsRed-Mito or CFP-Mito and scr-shRNA (control) or Mul1-shRNA, or Mul1shRNA together with Mul1*, followed by imaging at DIV14-15. Note that expressing Mul1* rescues mitochondrial morphology (size, $p=0.018$; aspect ratio, $p=0.011$) and membrane potential ($p<0.001$), compared to un-rescued neurons. Data were analyzed from the total number of neurons indicated in the bars in three independent experiments. Ordinary one-way ANOVA with Kruskal-Wallis-Test (**d**) or Dunnett multiple comparison test (**f**). Scale bars: 10 μm.

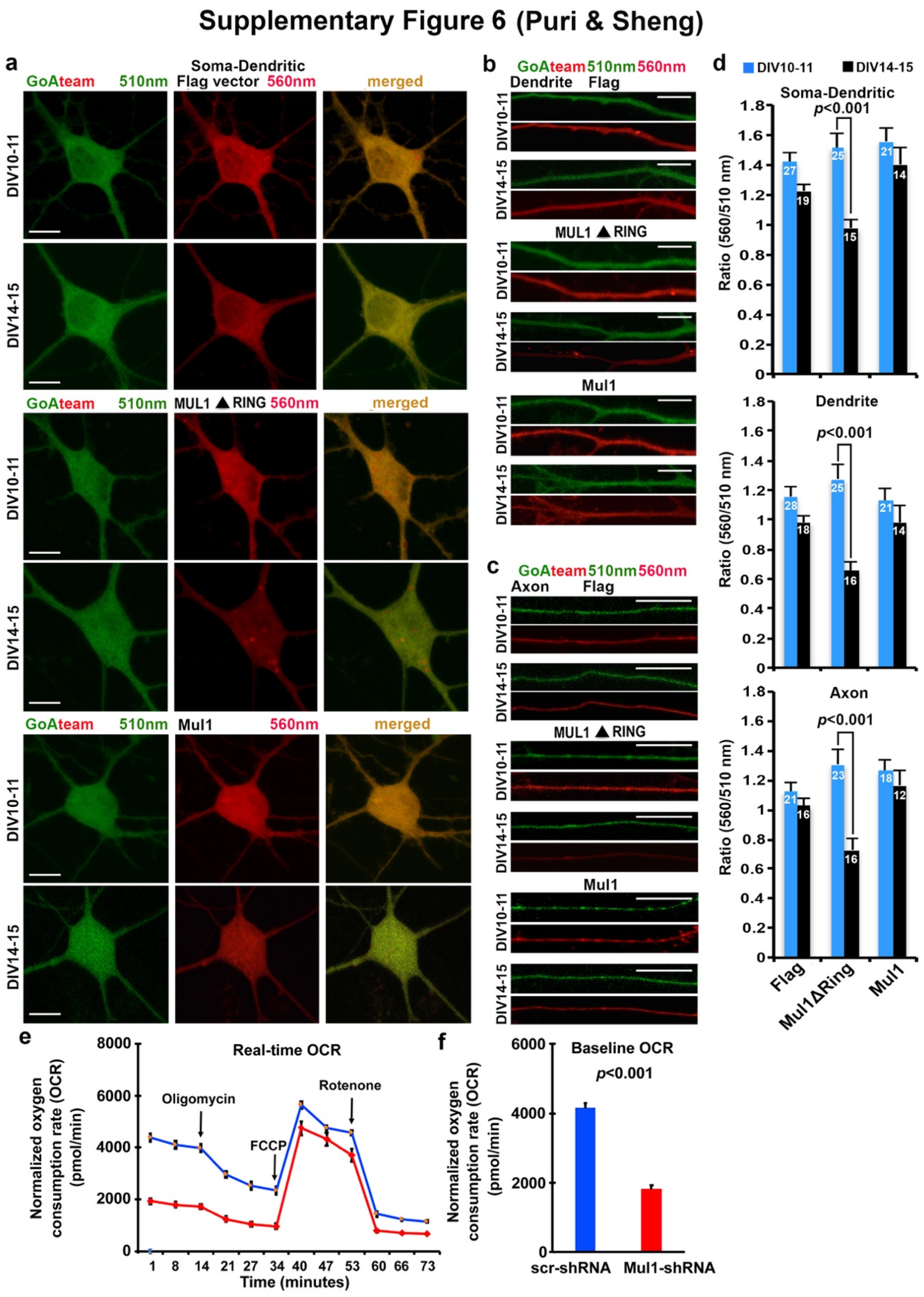

Supplementary Fig. 6 Mul1-deficient neurons display reduced mitochondrial bioenergetics. **a-d** Representative images and quantitative analysis showing reduced ATP levels in the soma-

dendritic (**a**), dendritic (**b**), and distal axonal compartments (**c**) in mature neurons (DIV14-15) expressing Mul1ΔRing. Cortical neurons were co-transfected at DIV7-8 with GoAteam2 and Flag, Flag-Mul1ΔRing, or Flag-Mul1. Live images were captured at DIV10-11 and DIV14-15. The relative ATP levels were measured by quantitative analysis of emission ratio (560/510) when excited with a 458nm laser line (**d**). Note that neurons expressing Mul1ΔRing display reduced cellular ATP levels selectively at DIV14-15 but not at DIV10-11. Data were analyzed from the total number of neurons shown in the bars in three experiments and are expressed as mean ± s.e.m. Unpaired Student'*s* t test. Scale Bars: 10 μm (**a**) and 5 μm (**b, c**). **e, f** Mitochondrial bioenergetics were assessed with Seahorse XF analyzer. Cortical neurons were infected at DIV7-8 with scr-shRNA or Mul1-shRNA. Real-time oxygen consumption rate (OCR) was measured at DIV14 before and after sequential injection of ATP synthase inhibitor oligomycin, mitochondrial uncoupler FCCP, and mitochondrial complex I inhibitor Rotenone into each cell well ($8x10^6$ cells) at the indicated time points (**e**) and the baseline OCR was analyzed before injection (**f**). Note a significant reduction in baseline OCR in Mul1-deficient neurons compared to control (p<0.001). A representative of three independent experiments is shown. Each point, n > 20 replicates.

Supplementary Figure 7 (Puri & Sheng)

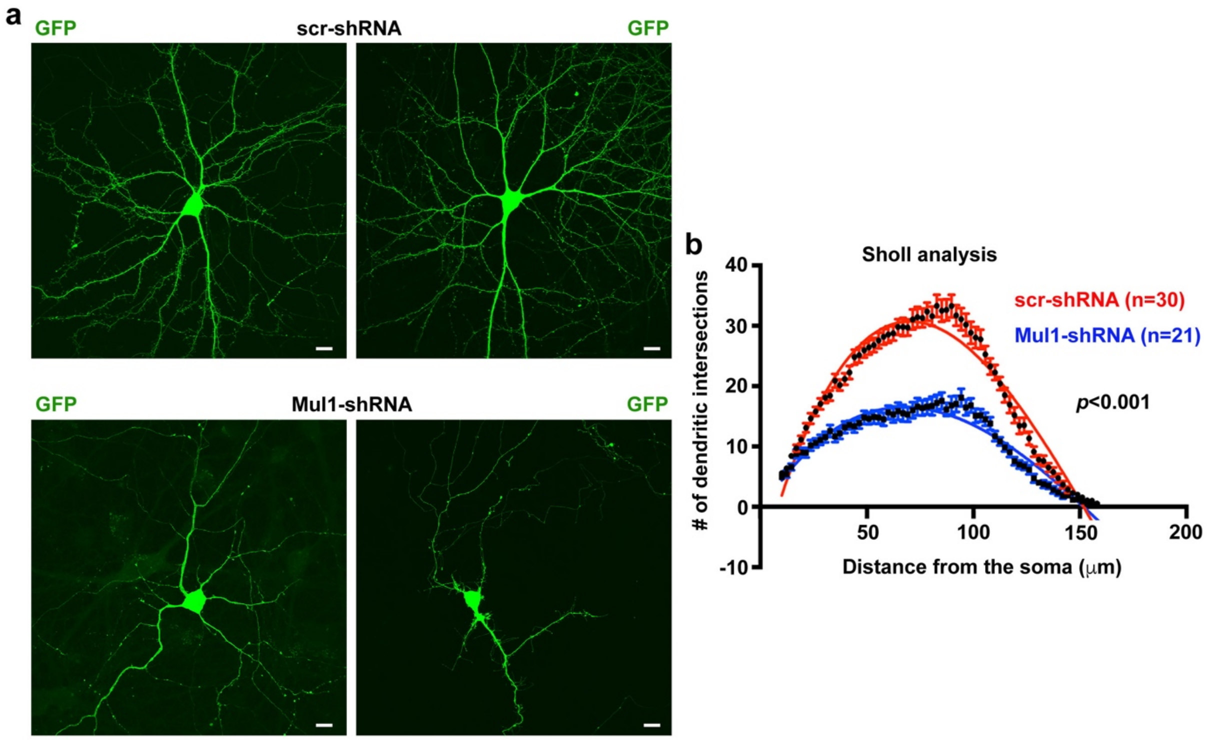

Supplementary Fig. 7 Mul1 deficiency affects dendritic arborization. Representative images (**a**) and Sholl analysis (**b**) showing reduced dendritic arborization in neurons with Mul1 depletion. Cortical neurons at DIV7 were co-transfected with GFP + shRNA, followed by live imaging of GFP at DIV14-15 to trace transfected neurons. The number of dendritic intersections was measured in various distances from the soma with Sholl analysis using ImageJ. Data were analyzed from the total number of neurons shown above the curves from three experiments and expressed as mean ± s.e.m. Two-way ANOVA test was used with centered third order polynomial curve fitting. Scale Bars: 10 μm.

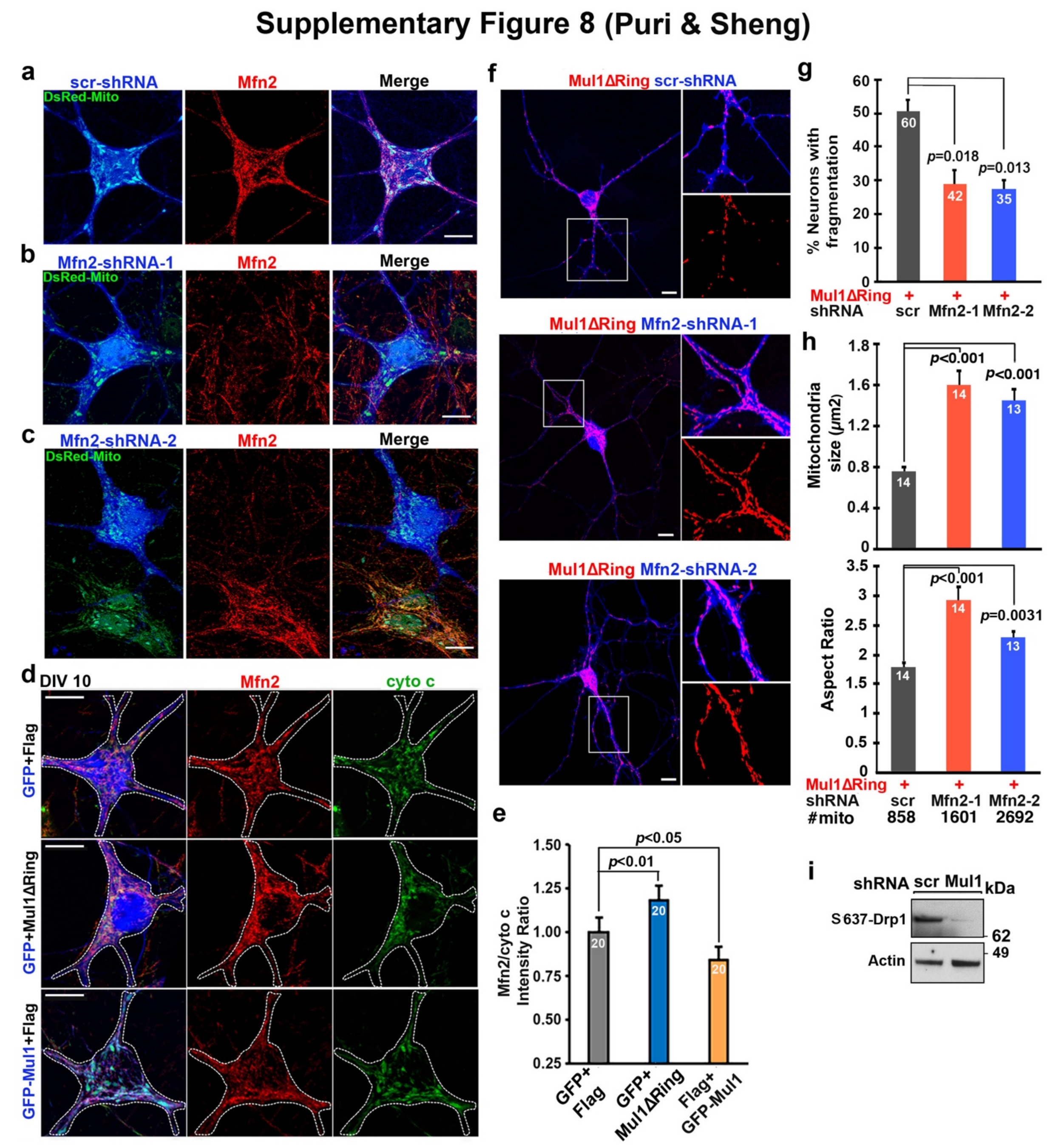

Supplementary Fig. 8. Mul1 deficiency enhances Mfn2 and activates Drp1. **a-c** Validation of the specificity of the anti-Mfn2 antibody. Representative images showing a robust reduction of Mfn2 fluorescent signals in neurons expressing Mfn2-shRNA-1 or Mfn2-shRNA-2 when compared to un-transfected cells within the same images. Cortical neurons were co-transfected at DIV7 with DsRed-Mito and shRNA as indicated, followed by immunostaining with an anti-Mfn2 antibody at DIV10. GFP-tagged shRNA was pseudo blue to trace transfected neurons and DsRed-Mito was

pseudo green to label mitochondria. **d, e** Representative images and quantitative analysis showing Mfn2 levels in soma-dendritic region of neurons expressing Mul1ΔRing or Mul1. Cortical neurons at DIV7 were co-transfected with GFP + Flag (upper), GFP + Flag-Mul1ΔRing (middle), or GFP-Mul1 + Flag (lower), followed by co-immunostaining of Mfn2 and cytochrome c at DIV10. GFP signals (pseudo blue) were used to trace transfected neurons. Relative Mfn2 levels on individual mitochondria were determined by measuring the intensity ratio of Mfn2/cyto c from the total number of neurons indicated in the bars and normalized to Flag control neurons. Note that Mul1 deficiency significantly increases the Mfn2/cyto c ratio while expressing Mul1 reduces the ratio, indicating a critical role of Mul1 in regulating mitochondria-targeted Mfn2 levels. **f-h** Representative images (**f**) and quantitative analysis of mitochondrial fragmentation (**g**) and morphology (**h**) showing the role of Mfn2 in mediating Mul1-deficient effect. Cortical neurons were transfected with Mul1ΔRing at DIV7-8, followed by the second transfection at DIV10-11 with Mfn2-shRNA-1, Mfn2-shRNA-2, or scr-shRNA. Mul1-targeted mitochondria were imaged at DIV14. GFP was pseudo blue. Note that depleting Mfn2 in Mul1-deficient neurons effectively rescues mitochondrial fragmentation and size. **i** Representative immunoblot showing a suppressed phosphorylation status of Drp1 at serine 637 in Mul1-depleted neurons. Data were analyzed from the total number of neurons shown in the bars in three experiments and are expressed as mean ± s.e.m. Ordinary one-way ANOVA with Dunnett multiple comparison test was used for comparing multiple groups. Scale Bars: 10 μm.

Supplementary Figure 9 (Puri & Sheng)

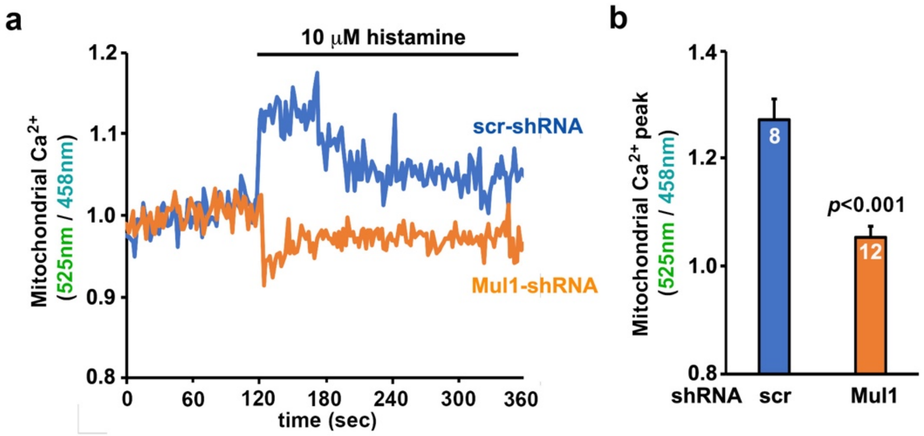

Supplementary Fig. 9 Mul1 depletion reduces mitochondrial Ca^{2+} uptake from the ER. Representative traces (**a**) and quantitative analysis of the average maximal Ca^{2+} peak (**b**) showing impaired mitochondrial Ca^{2+} uptake from the ER in Mul1-deficient neurons. Cortical neurons were co-transfected at DIV7-8 with pcDNA-4mtD3cpv and non-tagged scr or Mul1-shRNA, followed by live imaging at DIV10-11. The emission spectra of the ratiometric probe (525nm/458nm) were obtained by exciting 405nm following stimulation with 10 μM histamine. Note that Mul1-deficient neurons display a significant reduction in mitochondrial Ca^{2+} peak following Ca^{2+} release from the ER. Data were analyzed from 2-3 live neurons per field per trial and total number of trials are indicated in the bars and expressed as mean ± s.e.m. Unpaired Student's *t* test was used for comparing two groups.
