# Supplementary material for: Mul1 restrains Parkin-mediated mitophagy in mature neurons by maintaining ER-mitochondrial contacts
Source: Nat Commun. 2019 Aug 13;10:3645. doi: 10.1038/s41467-019-11636-5 (PMC6692330; doi:10.1038/s41467-019-11636-5)
Supplement: Supplementary file 3 — Reporting Summary [file 41467_2019_11636_MOESM3_ESM.pdf]

nature research

Corresponding author(s): Zu-Hang Sheng

Reporting Summary

Nature Research wishes to improve the reproducibility of the work that we publish. This form provides structure for consistency and transparency in reporting. For further information on Nature Research policies, see Authors & Referees and the Editorial Policy Checklist.

Statistical parameters

When statistical analyses are reported, confirm that the following items are present in the relevant location (e.g. figure legend, table legend, main text, or Methods section).

n/a	Confirmed	
☐	☒	The exact sample size (*n*) for each experimental group/condition, given as a discrete number and unit of measurement
☐	☒	An indication of whether measurements were taken from distinct samples or whether the same sample was measured repeatedly
☐	☒	The statistical test(s) used AND whether they are one- or two-sided<br>*Only common tests should be described solely by name; describe more complex techniques in the Methods section.*
☒	☐	A description of all covariates tested
☐	☒	A description of any assumptions or corrections, such as tests of normality and adjustment for multiple comparisons
☐	☒	A full description of the statistics including central tendency (e.g. means) or other basic estimates (e.g. regression coefficient) AND variation (e.g. standard deviation) or associated estimates of uncertainty (e.g. confidence intervals)
☐	☒	For null hypothesis testing, the test statistic (e.g. *F*, *t*, *r*) with confidence intervals, effect sizes, degrees of freedom and *P* value noted<br>*Give P values as exact values whenever suitable.*
☒	☐	For Bayesian analysis, information on the choice of priors and Markov chain Monte Carlo settings
☒	☐	For hierarchical and complex designs, identification of the appropriate level for tests and full reporting of outcomes
☒	☐	Estimates of effect sizes (e.g. Cohen's *d*, Pearson's *r*), indicating how they were calculated
☐	☒	Clearly defined error bars<br>*State explicitly what error bars represent (e.g. SD, SE, CI)*

Our web collection on statistics for biologists may be useful.

Software and code

Policy information about availability of computer code

Data collection	Confocal images were acquired using Olympus Software FV10-ASW (Photonics Media). STED image acquisition was done by using built-in Leica Application Suite (LASX) software. Sea horse data analysis was done using Agilent proprietary software. Acquired STED Images were deconvolved using a Leica built-in Huygens STED deconvolution software (Huygens).
Data analysis	Statistical analysis was done using GraphPad Prism 7 (GraphPad Software). Image quantification was using ImageJ (NIH). Co-localization analysis for STED imaging was done by using co-localization plug-in in Volocity 3D Image Analysis Software (PerkinElmer).

For manuscripts utilizing custom algorithms or software that are central to the research but not yet described in published literature, software must be made available to editors/reviewers upon request. We strongly encourage code deposition in a community repository (e.g. GitHub). See the Nature Research guidelines for submitting code & software for further information.

April 2018

Data

Policy information about availability of data

All manuscripts must include a data availability statement. This statement should provide the following information, where applicable:

- Accession codes, unique identifiers, or web links for publicly available datasets
- A list of figures that have associated raw data
- A description of any restrictions on data availability

The raw data sets generated during and/or analyzed during the current study are available from the corresponding author on reasonable request

Field-specific reporting

Please select the best fit for your research. If you are not sure, read the appropriate sections before making your selection.

[x] Life sciences [ ] Behavioural & social sciences [ ] Ecological, evolutionary & environmental sciences

For a reference copy of the document with all sections, see nature.com/authors/policies/ReportingSummary-flat.pdf

Life sciences study design

All studies must disclose on these points even when the disclosure is negative.

Sample size	Sample size choice was based on previous studies (ref.- 6,7,19,39), not predetermined by a statistical method.
Data exclusions	No data was excluded.
Replication	Statistical analysis was carried out using Prism 7 (GraphPad Software). Statistical analysis was conducted on data from three or more biologically independent experimental replicates. Data distribution was assumed to be normal, but this was not formally tested. Comparisons between groups were planned before statistical testing and target effect sizes were not predetermined. Error bars displayed on graphs represent the mean ± SEM of at least three independent experiments. Statistical significance was analyzed using unpaired student's t test between two different groups or Mann-Whitney test when samples sizes were small. For analysis between more than two groups, statistical significance was analyzed using One-Way ANOVA with Dunnet's or Tukey's post hoc multiple comparison test. All tests were two sided. *p<0.05, **p<0.01, and ***p<0.001 were considered significant.
Randomization	No randomization method was used.
Blinding	Data collection and Analysis was not performed blind.

Reporting for specific materials, systems and methods

Materials & experimental systems

n/a	Involved in the study
[x]	[ ] Unique biological materials
[ ]	[x] Antibodies
[ ]	[x] Eukaryotic cell lines
[x]	[ ] Palaeontology
[ ]	[x] Animals and other organisms
[x]	[ ] Human research participants

Methods

n/a	Involved in the study
[x]	[ ] ChIP-seq
[x]	[ ] Flow cytometry
[x]	[ ] MRI-based neuroimaging

Antibodies

Antibodies used	Rabbit polyclonal anti-MAP2 (Millipore; 1:1,000 for staining) (AB5622); Rabbit polyclonal anti-Mul1 (Sigma-Aldrich) (HPA017681; 1:500 for blotting); Rabbit polyclonal anti-p62/SQSTM1 (MBL International) (PM045, 1:500 for staining); Rabbit polyclonal anti-Hsp60 (Cell Signaling) (4870S; 1:1000 for blotting); Rat Monoclonal anti-Lamp1 (DSHB) (1D4B; 1:500 for staining); Mouse monoclonal anti-HA (Covance) (mms-101p; 1:500 for staining); Rabbit polyclonal anti-Mfn2 (Sigma-Aldrich) (M6319; 1:1,000 for blotting; 1:250 for staining); Mouse Monoclonal anti-Flag (Sigma-Aldrich) (F1804; 1:500 for staining); Ser637-Drp1 (rabbit; 4867; Cell Signaling), mCherry (mouse; ab125096; abcam), Rabbit polyclonal anti-Flag (Sigma-Aldrich) (F7425; 1:500 for staining); Rabbit Polyclonal anti-Tom20 (Santa Cruz) (sc-11415; 1:4,000 for blotting; 1:100 for staining for nano-microscopy); Mouse monoclonal anti-Ubiquitin (Santa Cruz) (sc-8017; 1:1,000 for blotting); Chicken polyclonal anti-Myc (Abcam) (ab172; 1:250 for staining); Mouse Monoclonal anti-Cytochrome c (BD Biosciences) (556432; 1:500 for staining); Rabbit polyclonal anti-Drp1 (Cell Signaling) (8570; 1:50 for staining; 1:500 for blotting), Mouse; Rabbit; Chicken Alexa Fluor 488- or 546- or 594- or 633-conjugated secondary antibodies (Invitrogen; 1:400 for staining), Mouse IgG- or Rabbit IgG-HRP (GE HealthCare; 1:5000 for blotting), and Nanogold (Nanoprobe) (2004; 1:200 for staining for electron microscopy)
Validation	Data provided in the manuscript.

Eukaryotic cell lines

Policy information about cell lines

Cell line source(s)	HEK293T from ATCC, Cat. # (CRL-3216)
Authentication	HEK293T cell line was not authenticated
Mycoplasma contamination	All cell lines tested were negative for mycoplasma contamination
Commonly misidentified lines (See ICLAC register)	No misidentified cell lines used in the study

Animals and other organisms

Policy information about studies involving animals; ARRIVE guidelines recommended for reporting animal research

Laboratory animals	Used for the experiments were embryonic day 18 (E18) embryos from female mice and rat. Mouse strain used in the study was C57BL/6J (Charles River) (Strain Code: 027) and the rat strain SAS SD (Charles River) (Strain Code: 400).
Wild animals	Pregnant E18 mice or rats were narcotized with carbon dioxide (in-house) and then decapitated using a guillotine as a second means of euthanasia. Embryos were collected and then decapitated in dissection buffer. The hippocampi or cortex of each brain was then dissected out, trysinized, triturated, and plated at proper density for various experiments.
Field-collected samples	Animal care and use were carried out in accordance with NIH guidelines, NIH Manual 3040-2, Guide for the Care and Use of Laboratory Animals (National Research Council), Institutional Animal Care and Use Committee Guidebook (ARENA and OLAW) and approved by the NIH, NINDS/NIDCD Animal Care and Use Committee on 3/10/2015 (ASP# 1250-15)
