# Supplementary material for: Mul1 restrains Parkin-mediated mitophagy in mature neurons by maintaining ER-mitochondrial contacts
Source: Nat Commun. 2019 Aug 13;10:3645. doi: 10.1038/s41467-019-11636-5 (PMC6692330; doi:10.1038/s41467-019-11636-5)
Supplement: Supplementary file 4 — Description of Additional Supplementary Files [file 41467_2019_11636_MOESM4_ESM.docx]

**Description of Additional Supplementary Files**

File Name: Supplementary Movie 1

Description: (Related to Fig. 5a). 3D STED surface reconstitution showing ER-Mito contacts in a neuron expressing Flag vector. Cortical neurons were co-transfected at DIV7 with the ER marker GFP-Sec61b (green) and Flag control. Neurons were fixed at DIV11 and immunostained for Tom20 (red). Super-resolution images were captured using dual-color 3D-STED nanoscopy. The co-localization between ER and mitochondria was measured in distal dendritic regions. The yellow color represents overlap between ER and mitochondria.

File Name Supplementary Movie 2

Description: (Related to Fig. 5a). 3D STED surface reconstitution showing reduced ER-Mito contacts in a neuron expressing Flag-Mul1ΔRing. Cortical neurons were co-transfected at DIV7 with the ER marker GFP-Sec61b (green) and Flag-Mul1ΔRing. Neurons were fixed at DIV11 and immunostained for Tom20 (red). Super-resolution images were captured using dual-color 3D-STED nanoscopy. The co-localization between ER and mitochondria was measured in distal dendritic regions. The yellow color represents overlap between ER and mitochondria.

File Name Supplementary Movie 3

Description: (Related to Fig. 5a). 3D STED surface reconstitution showing increased ER-Mito contacts in a neuron expressing Flag-Mul1. Cortical neurons were co-transfected at DIV7 with the ER marker GFP-Sec61b (green) and Flag-Mul1. Neurons were fixed at DIV11 and immunostained for Tom20 (red). Super-resolution images were captured using dual-color 3D-STED nanoscopy. The co-localization between ER and mitochondria was measured in distal dendritic regions. The yellow color represents overlap between ER and mitochondria.
