# Supplementary material for: Mul1 restrains Parkin-mediated mitophagy in mature neurons by maintaining ER-mitochondrial contacts
Source: Nat Commun. 2019 Aug 13;10:3645. doi: 10.1038/s41467-019-11636-5 (PMC6692330; doi:10.1038/s41467-019-11636-5)
Supplement: Supplementary file 2 — Peer Review File [file 41467_2019_11636_MOESM2_ESM.pdf]

Reviewers' comments:

Reviewer #1 (Remarks to the Author):

In this manuscript, Puri and Sheng suggest a role for Mul1 in maintaining mitochondrial homeostasis under mild-stress conditions by modulating mitochondrial morphology and the integrity of the ER-mito contacts. In Mul1-depleted neurons, impaired ER-mito coupling activates Drp1 to boost mitochondrial fragmentation. Protecting the ER-mito sites by expressing PTPIP1 reduces engagement of Parkin suggesting the signalling between Mul1 and mitochondrial fusion/fission machinery is upstream of Parkin-mediated mitophagy. Though the data in the manuscript is extensive, the experiments lack proper controls and statistics as outlined below.

1. In the experiments where multiple comparisons are made, t-tests were used to compute statistics rather than ANOVA tests with correction for multiple comparisons.
2. The increased localization of Parkin to mitochondria in Figure 1b is not convincing. In the zoomed-in insets, there are multiple CFP-Mito pixels that are not mCherry-Parkin positive. The same comment for Figure 1d - adjacent pixels are visible but not a major overlap.
3. Much of the translocation of Parkin data rely on overexpression of Parkin. The authors should stain the neuronal cultures for endogenous Parkin and mitochondrial markers to show endogenous Parkin translocation is affected by Mul1 oe and lof.
4. The authors state the quantifications were performed unblinded, which can be a problem where small changes (e.g. in % of neurons displaying a certain phenotype) are reported. For example, in Figure 1, % of neurons displaying translocation of Parkin seems subjective since there can be a large variation of translocation of Parkin between neurons. The authors should quantify the intensity of Parkin signal on mitochondria rather than quantifying the percent of neurons displaying Parkin translocation.
5. The specificity of the Mul1-shRNA in the Parkin translocation assays is not studied. The authors should knockdown Mul1 and rescue the increased Parkin translocation phenotype with RNAi-resistant wt and mutant forms of Mul1.
6. Throughout the manuscript, additional non-specific controls are not properly included. For example, in Figure 1, the specificity controls are not shown for translocation of Parkin i.e. mCherry attached to a non-specific protein as well as mutant forms of Parkin that are deficient in translocation. Another example is the experiment in Figure 4a, which lacks controls (non-silencing shRNA, non-specific protein expression, wt mul1). Similarly, mock transfection is not a proper control for overexpression experiments. Please express a non-specific protein as a control.
7. The authors did not show quantification of mitophagy in Figure 1f. In addition, a more dynamic measure of mitophagy (e.g. mt-Keima) should be used rather than static images of mitochondrial markers localizing to LAMP1/LC3.
8. The biphasic change in mitochondrial morphology in Figure 2a could be explained by longer duration of expression of the shRNAs. The authors should rescue the effects of Mul1-shRNA using RNAi-resistant cDNAs (similar to the comment 5). Same comment for figure 3.
9. I suggest measuring mitochondrial function using the Seahorse technology in cultures infected with mul1 kd and oe viruses.
10. The biphasic effect of changing mul1 levels on mito morphology is not properly quantified. The authors show only the significance btwn div 10-11 and div 14-15 within each measure. Please show significance btw blue bars across conditions (e.g scrambled vs mul1-shrna) to test the significance of phase I. Same comment for phase II for the black bars. In addition, ANOVA rather than t-tests should be used.
11. Please show a loading control for the mfn2 levels shown in Figure 5d-knockdown experiment.
12. The work in Figure 5 and 6 relies on overexpression which may lead to non-specific effects. Please study ER-mito contact site morphology and function in Mul1-shRNA-infected/transfected neurons. Same comment for Figure 8a,b.

13. Does pDrp1 levels change in neurons where Mul1 is knocked down?

Minor points:

1. In Supplementary Figure 1, mitochondrial localization of the dTM1/2 mutant is still evident.
2. How is the dominant-negative effect of Mul1-dRING explained in Figure 1e?
3. A delayed mitophagic response in neurons with mitochondrial depolarization could be due to the intrinsic reliance of neurons on mitochondria to generate ATP to fuel cellular processes, including Parkin translocation and mitophagy.
4. Does dTM1/2 affect localization of Parkin to mito in AA-treated neurons?
5. Figure S3d is hard to interpret without knowing the relative expression levels of GFP-Mul1 and the dRING mutant. The reduced effect of dRING can be explained by a potential reduction in the expression of this construct upon introduction of wt mul1. Thus, the authors should introduce a non-specific protein as a control.

Reviewer #2 (Remarks to the Author):

This study by Puri et al discovers a novel mechanism underlying mitochondrial maintenance during chronic stress. The mitochondrial E3 ligase Mul1 suppresses Mfn2 to maintain ER-mito integrity, which is crucial to prevent mitophagy. Overall this is an interesting study and the experiments were well designed and performed. However, at least two key experiments are missing. Because mitophagy is a very dynamic process and cannot be simply demonstrated by co-localization of overexpressed mcherry-Parkin and CFP-mito, the authors need to show whether mitophagy kinetics is faster in Mul1 knockdown/Mfn2-overexpressing cells compared to controls. Also, does knocking down Mfn2 in Mul1 knockdown neurons rescue the mitochondrial phenotype?

In addition, there are a few important technical concerns that need to be addressed:

In Fig. 1, the chronic stress was triggered by a low dose of AA for a few hours. However, AA should be combined with oligomycin for such treatment because membrane potential reverses without oligomycin (PMID: 26266977). The authors will need to measure the membrane potential during the treatment as well.

The images in Fig. 1a-d are not consistent with the quantification in e. From looking at the images alone, Fig1 a, b, d look very similar (different from c)–all show dramatic change of Parkin from being cytosolic before treatment to punctate after treatment. The quantification in e is the % of total neurons, while a-d show only one cell. Larger images that contain multiple neurons are needed.

Fig. 1f needs a wild-type control and quantification.

Fig. 4d needs loading control and quantification.

Flag-Mul1ΔRing was overexpressed in neurons with endogenous Mul1, so it is not “loss of function” as stated throughout the paper.

Fig. 5: Because Flag-Mul1ΔRing was overexpressed, an important control here is full-length Flag-Mul1 overexpression. Also, the ER-mito contacts should be examined in Mul1 knockdown cells.
Page 11: "Altogether, our super-resolution imaging and ultrastructural analysis consistently indicate impaired ER-Mito contacts in Mul1 loss-of-function neurons, likely through elevated Mfn2 activity". This speculation should be tested by knocking down Mfn2.

Fig. 4b, 7b: The antibodies for ICC need to be validated, and the images need to be quantified.

Does Mfn2 overexpression cause more ER-mito contacts and faster mitophagy kinetics?

Reviewer #3 (Remarks to the Author):

In this manuscript, the authors reveal a Mul1-Mfn2 signaling pathway that maintains neuronal mitochondrial integrity under mild stress conditions. They show that loss of Mul1 increases the level of Mfn2, which acts as an ER-Mito tethering antagonist, impairing mitochondrial bioenergetics and Ca2+ homeostasis. They further show that reduced ER-Mito contact leads to increased cytoplasmic Ca2+, activating calcineurin and inducing Drp1-dependent mitochondrial division and Parkin-mediated mitophagy. Interestingly, expressing ER-Mito tethering protein PTPIP51 suppresses Parkin-mediated mitophagy. The authors propose that the Mul1-Mfn2 pathway plays a checkpoint role in maintaining mitochondrial integrity and/or recovering stressed mitochondria before Parkin-mediated mitophagy is activated. Overall, the study is well performed, and the conclusion, if further strengthened, will add significantly to our understanding of mitochondrial protection under stress.

Major Comments:

1. The causal role of Mfn2 in mediating mul loss of function (lof) effect on mito-ER contact is not established. Effect of mfn2 RNAi in rescuing mul lof effect should be tested.

2. Although mito-ER contact is affect and mito-Ca2+ homeostasis is altered, whether mito-ER Ca2+ is causally involved in mul lof effect will require further study. Does genetic manipulation of mito-ER Ca2+ transfer by IP3R/VDAC/MCU have any effect?

3. The role of Mfn in Mito-ER contact is controversial. Recent studies suggest that Mfn may be differentially involved in regulating different types of mito-ER contacts: the "narrow" vs. "wide" contacts. The authors are recommended to carefully characterize their EM pictures to assess the effect of mul lof on these different types of contacts.

4. The analysis of effects of mul on neuronal function is limited to mitochondrial morphology, membrane potential, and ATP production. More analysis on neuronal communication or survival may strengthen the physiological significance of this study.

5. In other settings, increased mito-ER contacts and increased Ca or lipid transfer from ER to mitochondria has been implicated in Pink1/parkin lof. This should be specifically discussed, especially considering that Mul and Pink1/Parkin has been proposed by the authors to act in parallel pathways to preserve mitochondrial function through the same target Mfn.

Minor comments:

1. Specificity of ShRNA studies should be demonstrated by rescue with shRNA-resistant cDNA.

2. Mechanism and specificity of Mul regulation of Mfn2. Is Mfn1 not regulated the same way as Mfn2?

Reviewer #1 (Remarks to the Author):

In this manuscript, Puri and Sheng suggest a role for Mul1 in maintaining mitochondrial homeostasis under mild-stress conditions by modulating mitochondrial morphology and the integrity of the ER-mito contacts. In Mul1-depleted neurons, impaired ER-mito coupling activates Drp1 to boost mitochondrial fragmentation. Protecting the ER-mito sites by expressing PTPIP1 reduces engagement of Parkin suggesting the signaling between Mul1 and mitochondrial fusion/fission machinery is upstream of Parkin-mediated mitophagy. Though the data in the manuscript is extensive, the experiments lack proper controls and statistics as outlined below.

1. In the experiments where multiple comparisons are made, t-tests were used to compute statistics rather than ANOVA tests with correction for multiple comparisons.

At suggested by the reviewer, we have applied Ordinary one-way ANOVA with Dunnett multiple comparison test for multiple comparisons in **Fig. 1e, 1i, 2e, 3g, 4c, 5b, 6d, 8d, 9c, S4d** of the revision.

2. The increased localization of Parkin to mitochondria in Figure 1b is not convincing. In the zoomed-in insets, there are multiple CFP-Mito pixels that are not mCherry-Parkin positive. The same comment for Figure 1d - adjacent pixels are visible but not a major overlap.

We replaced the images to better represent Parkin translocation to stressed mitochondria (**Fig. 1a-d**). Our previous studies in mature cortical neurons demonstrated that Parkin-mediated mitophagy is only observed in a small portion of neurons and that not all mitochondria recruit Parkin following mitochondrial depolarization (Cai et al., *Current Biology* 2012; Lin et al., *Neuron* 2017). Consistently, the Schwarz group reported that mitophagy is observed in a small portion of axonal mitochondria following acute depolarization (Ashrafi et al., *JCB* 2014). These observations indicate that post-mitotic cells like neurons have unique mechanisms that can maintain mitochondrial integrity under mitochondrial stress, rather than activating global Parkin-mediated mitophagy, a unique phenotype readily observed in many non-neuronal cell types.

To further confirm enhanced mitophagy in neurons with Mul1 deficiency, we followed reviewer's suggestion (point #7) by measuring dynamic mitophagy using mt-Keima to examine the targeting of fragmented mitochondria to acidic lysosomes under Mul1 depletion with shRNA (**Fig 1h**) and overexpression of Mul1 mutant (**Fig. 1i**).

3. Much of the translocation of Parkin data rely on overexpression of Parkin. The authors should stain the neuronal cultures for endogenous Parkin and mitochondrial markers to show endogenous Parkin translocation is affected by Mul1 oe and lof.

The reviewer raised a critical question in the neuronal mitophagy field. We and others, including the Richard Youle lab, could not find any commercially available anti-Parkin antibody that reliably detects endogenous Parkin in cultured neurons. At suggested by the reviewer (see below), we instead measured dynamic mitophagy using mt-Keima under Mul1 depletion with shRNA (**Fig. 1h**) and overexpression of Mul1 loss-of-function mutant (**Fig. 1i**). These newly added data consistently support that Mul1 loss-of-function enhances dynamic mitophagy in neurons.

4. The authors state the quantifications were performed unblinded, which can be a problem where small changes (e.g. in % of neurons displaying a certain phenotype) are reported. For

example, in Figure 1, % of neurons displaying translocation of Parkin seems subjective since there can be a large variation of translocation of Parkin between neurons. The authors should quantify the intensity of Parkin signal on mitochondria rather than quantifying the percent of neurons displaying Parkin translocation.

We apologize for not clarifying this. Our previous studies in mature cortical neurons demonstrated that (1) Parkin-mediated mitophagy is observed only in a small portion of neurons following acute mitochondrial depolarization and (2) Parkin translocation from the cytosol to depolarized mitochondria occurs much more slowly than in non-neuronal cell types (Cai et al., Current Biology 2012; Lin et al., Neuron 2017). Consistently, the Schwarz group reported that mitophagy is observed in a small portion of axonal mitochondria following acute depolarization with Antimycin A (Ashrafi et al., *JCB* 2014). These observations indicate that post-mitotic cells like neurons have unique mechanisms that can maintain and/or recover mitochondrial integrity at least in the early stages of mitochondrial stress, rather than rapid and global elimination of dysfunctional mitochondria through Parkin-mediated mitophagy that was observed in many non-neuronal cell types. We provided the rationale for these measurements on **page 7**.

To examine whether Mul1 loss-of-function facilitates neuronal mitophagy in the current study, we instead measured the percentage of neurons displaying Parkin translocation under mild depolarization condition (**Fig. 1a-e**). Our hypothesis is that neurons have an intrinsic mechanism for recovering stressed mitochondria through the mitochondria-resident Mul1 pathway before recruiting cytosolic Parkin. If this checkpoint mechanism fails (Mul1 loss-of-function), Parkin-mediated mitophagy is then activated in more neurons. In addition, Mul1-deficiency induced fragmented mitochondria; some of which were subjected to autophagic clearance by recruiting the autophagic markers p62 and LC3 (**Supl Fig. 2**), or sorted into lysosomes (**Fig. 1g**). These data collectively suggest that Mul1 is required to recover stressed mitochondria, thus suppressing Parkin recruitment to mitochondria for degradation through mitophagy under stress conditions.

At suggested by the reviewer, we also measured the intensity of Parkin signal on mitochondria by performing Pearson's correlation coefficient in Mul1 KD neurons compared to scrambled control. Newly added data (**Fig. 1f**) indicate a higher correlation of Parkin signal on mitochondria in shRNAi-Mul1 neurons (R=0.58) relative to control neurons (R=0.46, $p<0.05$) under mild stress conditions. Since we did not observe a robust change in Parkin correlation on mitochondria between neurons expressing scr-shRNA versus Mul1-shRNA, variation of Parkin translocation is rather limited when neuronal mitophagy is activated under our conditions. We also describe the result on **page 7**.

5. The specificity of the Mul1-shRNA in the Parkin translocation assays is not studied. The authors should knockdown Mul1 and rescue the increased Parkin translocation phenotype with RNAi-resistant WT and mutant forms of Mul1.

At suggested by the reviewer, we generated an shRNA-resistant Mul1 mutant (Mul1*) by substituting eight third-code nucleotides in Mul1-shRNA-targeting sequence (A210T, T213A, G216A, G219A, T222A, T225A, A228G, and A231G) without changing amino acid sequence. While Mul1-shRNA suppresses endogenous Mul1 and significantly deplete exogenous Flag-Mul1 ($p<0.001$), it fails to suppress silent mutant Mul1* ($p=0.68$), thus indicating an shRNA-resistant Mul1 (Mul1*). We added this data in **Supl Fig. 5a, 5b**.

Given the critique of the reviewer on our Parkin translocation quantitative data, we instead tested the specificity of shRNA knockdown with Mul1* using more robust assays: mitochondrial fragmentation and loss of membrane potential. Our newly added data demonstrate that mitochondrial fragmentation (**Supl Fig. 5c, 5d)** and mitochondrial depolarization (**Supl Fig. 5e, 5f)** in Mul1-deficinet neurons (Mul1-shRNA) can be effectively rescued by co-expressing Mul1*. The rescue of two Mul1-depleted phenotypes consistently indicate the specificity of Mul1-shRNA, thus excluding an off-target effect. We described the results on **page 9**.

6. Throughout the manuscript, additional non-specific controls are not properly included. For example, in Figure 1, the specificity controls are not shown for translocation of Parkin i.e. mCherry attached to a non-specific protein as well as mutant forms of Parkin that are deficient in translocation. Another example is the experiment in Figure 4a, which lacks controls (non-silencing shRNA, non-specific protein expression, wt mul1). Similarly, mock transfection is not a proper control for overexpression experiments. Please express a non-specific protein as a control.

We followed the reviewer's excellent suggestions by providing following three new lines of experiments:

First, we added more controls in the following experiments. (1) we provided quantitative data showing the percentage of mitochondria co-localized with LAMP1 under control condition (scr-shRNA) and Mul1-depletion conditions (Mul1-shRNA) (**Fig.1g**). (2) in newly added mt-Keima experiments, we include proper controls to measure dynamic mitophagy - targeting of fragmented mitochondria to acidic lysosomes. Neurons expressing Mul1-shRNA displayed significantly higher dynamic mitophagy relative to neurons expressing scr-shRNA (p<0.001) (**Fig. 1h**). Consistently, neurons overexpressing Mul1ΔRing displayed more dynamic mitophagy relative to neurons overexpressing WT Mul1 or Flag control (p<0.001) (**Fig. 1i**). (3) we tested the specificity of shRNA knockdown with shRNA-resistant Mul1*. Mitochondrial fragmentation and mitochondrial depolarization (**Supl Fig. 5)** in Mul1-deficinet neurons (Mul1-shRNA) can be effectively rescued by co-expressing Mul1*, indicating the specificity of Mul1-shRNA, thus excluding an off-target effect.

Second, we provided new data in **Fig. 4a** by adding three more co-expression control groups, including Mul1 with Mfn1/2, scr-shRNA with Mfn1/2; and non-specific protein control GFP-Sec61-β (ER membrane marker) with Mfn1/2. The new analysis indicates no significant change in percentage of neurons with mitochondrial fragmentation between groups of (1) Mfn1 vs Mfn2; (2) Sec61β/Mfn1 vs Sec61β/Mfn2; (3) scr-shRNA/Mfn1 vs scr-shRNA/Mfn2; and (4) Mul1/Mfn1 vs Mul1/Mfn2. Therefore, these data support our conclusion that elevated expression of Mfn2, but not Mfn1, selectively accelerated mitochondrial fragmentation in Mul1-deficient neurons (ΔRING or Mul1-shRNA) even at an early stage (DIV10).

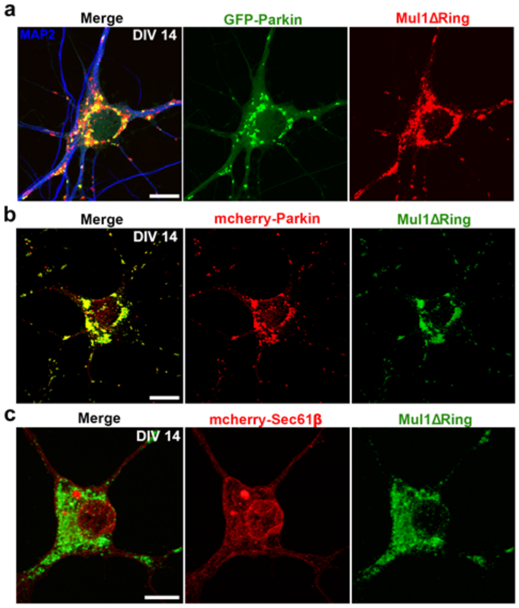

Third, we alternatively constructed GFP-Parkin and mCherry-Sec61β as positive and negative controls for

mitochondria-targeting of mCherry-Parkin. While both GFP-Parkin and mCherry-Parkin were recruited to fragmented mitochondria that were labeled by Mul1ΔRing, overexpressed mCherry-Sec61β was not targeted to fragmented mitochondria in the same Mul1-deficeint neurons, thus excluding non-specific targeting of mCherry-tagger. Since we have added a total of **18** new experimental data in the current revision, we prefer to show these representative images to this reviewer.

7. The authors did not show quantification of mitophagy in Figure 1f. In addition, a more dynamic measure of mitophagy (e.g. mt-Keima) should be used rather than static images of mitochondrial markers localizing to LAMP1/LC3.

We provided quantitative data showing the percentage of mitochondria co-localized with LAMP1 under control (scr-shRNA) and Mul1 depletion conditions (Mul1-shRNA) (**Fig 1g**). As suggested by the reviewer, we also measured dynamic mitophagy by using mt-Keima to confirm the targeting of fragmented mitochondria to acidic lysosomes under Mul1 depletion with shRNA (**Fig 1h**) and overexpression of Mul1 mutant (**Fig. 1i**).

Mt-Keima is a ratiometric pH-sensitive fluorescent protein that is targeted into the mitochondrial matrix. Keima has an excitation spectrum that changes according to pH. A short wavelength (458 nm) is predominant for excitation in a neutral environment, whereas a long wavelength (561 nm) is predominant in an acidic environment. When mitochondria are healthy, mt-Keima displays a low fluorescence ratio (561nm/458nm) (lysosomal red/mitochondrial green); however, when mitochondria are fragmented (damaged) and engulfed by acidic lysosomes (mitophagy), mt-Keima display a high fluorescence ratio (strong red signal). Since mt-Keima is resistant to lysosomal proteases, it allows for measurement of dynamic and cumulative mitophagy process.

We followed the well-established protocol (Bingol et al., *Nature* 2014; Katayama H et al., *Chem Biol*. 2011) to assess dynamics of mitophagy by measuring relative ratio of the area of lysosomal red signal / mitochondrial green signal within the cell body, here, referred to as "**mitophagy index**". These newly added data consistently support our conclusion that Mul1 loss-of-function enhances mitophagy in neurons. We also described this assay and results on **pages 7-8**.

Reference:
Bingol B et al. The mitochondrial deubiquitinase USP30 opposes parkin-mediated mitophagy. Nature 510, 370–5 (2014)
Katayama H et al. A sensitive and quantitative technique for detecting autophagic events based on lysosomal delivery. Chem Biol. 18, 1042-52 (2011)

8. The biphasic change in mitochondrial morphology in Figure 2a could be explained by longer duration of expression of the shRNAs. The authors should rescue the effects of Mul1-shRNA using RNAi-resistant cDNAs (similar to the comment 5). Same comment for figure 3.

We followed the suggestion of the reviewer to co-express the shRNA-resistant Mul1 mutant (Mul1*) in neurons expressing Mul1-siRNA. Our new data show that mitochondrial fragmentation in Mul1-depleted neurons was partially reversed by expressing Mul1* (**Supl Fig. 5c, 5d)**. In addition, as the reviewer also asked, mitochondrial depolarization in Mul1-deficinet neurons (Mul1-shRNA) was effectively rescued by co-expressing Mul1* (**Supl Fig. 5e, 5f)**, indicating the specificity of Mul1-shRNA, thus excluding an off-target effect.

9. I suggest measuring mitochondrial function using the Seahorse technology in cultures infected with mul1 kd and oe viruses.

Our study is focused on a biphasic change in mitochondrial morphology in individual neurons with Mul1 loss-of-function, from mitochondrial hyperfusion at DIV10-11 to mitochondrial fragmentation at DIV14-15 **(Fig. 2, Supl Fig. 4)**. To determine association of this biphasic change with mitochondrial integrity, we further applied two established approaches in the neuronal mitochondrial field: (1) measuring mitochondrial membrane potential using TMRE **(Fig. 3)**, and (2) measuring ATP levels using a FRET-based ATP probe in individual neurons under various conditions **(Supl Fig. 6)**. Our results consistently suggest a loss of mitochondrial integrity in neurons with Mul1 loss-of-function compared to neurons with Mul1 overexpression.

As suggested by the reviewer, we applied Seahorse XF analyzer to assess mitochondrial bioenergetics in neurons infected with scr-shRNA (control) or Mul1-shRNA. Real-time analysis of oxygen consumption rate (OCR) was measured in cortical neurons before and after sequential injection of ATP synthase inhibitor oligomycin, mitochondrial uncoupler FCCP, and mitochondrial complex I inhibitor Rotenone. This analysis consistently indicates a significant decrease in basal mitochondrial OCR in Mul1-deficient neurons compared to control (p<0.001) (**Supl Fig. 6e, 6f**). In addition, suggested by the reviewer 3, we added new data to demonstrated that such an impaired mitochondrial integrity affects neuronal cell connectivity and dendritic arborization pattern (**Supl Fig. 7**). We described these new data on **page 10**.

10. The biphasic effect of changing mul1 levels on mito morphology is not properly quantified. The authors show only the significance btwn div 10-11 and div 14-15 within each measure. Please show significance btw blue bars across conditions (e.g scrambled vs mul1-shrna) to test the significance of phase I. Same comment for phase II for the black bars. In addition, ANOVA rather than t-tests should be used.

We followed the reviewer's suggestion by providing new quantification (**Fig. 2e**) showing relative changes in dendritic mitochondrial size across different conditions both for phase I (DIV10-11) and phase II (DIV14-15). We used t-test for two group significance (scr-shRNA versus Mul1-shRNA) and Ordinary one-way ANOVA with Dunnett multiple comparison test for comparing GFP with other three groups (Ring, H319A, and Mul1). This new analysis consistently supports our notion that Mul1 loss-of-function induces mitochondrial hyperfusion at phase I and mitochondrial fragmentation at phase II. We made this new statement on **page 9**.

11. Please show a loading control for the mfn2 levels shown in Figure 4d-knockdown experiment.

We added Mfn2 loading control on **Fig 4d** (Mul1 KD experiments).

12. The work in Figure 5 and 6 relies on overexpression which may lead to non-specific effects. Please study ER-mito contact site morphology and function in Mul1-shRNA-infected/transfected neurons. Same comment for Figure 8a, b.

Immuno-EM ultrastructural analysis of the ER-Mito contact is a time-consuming and resource competitive work (in shared NINDS EM facility). To collectively address similar concerns from all three reviewers, we expanded our immune-EM study in the past three months by adding four new groups: including neurons expressing (1) full-length Mul1, (2) knocking down Mul1, (3) Mfn1, and (4) Mfn2. Our new data consistently demonstrate that while expressing Mul1 enhances ER-Mito contact (p<0.001), expressing Mul1ΔRing or knocking down Mul1 in neurons

significantly reduces the ER-Mito contact (p=0.01, p<0.05, respectively). In addition, overexpressing Mfn2, but not Mfn1, also inhibits the ER-Mito contact (p<0.001). Altogether, our super-resolution imaging and ultrastructural analysis consistently indicate that (1) Mul1 protects neuronal mitochondria integrity by maintaining ER-Mito contacts; (2) impaired ER-Mito contacts in Mul1 loss-of-function neurons is mediated through elevated Mfn2 expression. Our new immune-EM data were added in revised **Fig. 5d-5g** and described in text on **page 13.**

As for Figs 6 and 8 asked by the reviewer, it is technically challenging to trace Mul1 knockdown neurons expressing GFP-tagged shRNA. We have applied a genetically encoded FRET-based ratiometric probe (4mtD3cpv) to examine mitochondrial Ca^{2+} levels (**Fig. 6**) and a cytoplasm-targeted FRET-based ratiometric Ca^{2+} probe (D3cpv) to measure cytosolic Ca^{2+} levels (**Fig. 8**). The GFP excitation/emission wavelengths (488/509 nm) interfere with the excitation/emission wavelengths (458/525 nm) of these two calcium probes.

Our current study consistently demonstrates that knockdown of Mul1 or expression of Mul1 loss-of-function mutant Mul1ΔRing both induced similar phenotypes in cortical neurons, including (1) enhanced Parkin translocation to mitochondria (**Fig. 1b-1f**); (2) induced a bi-phasic change in mitochondrial morphology (**Fig. 2**); and (3) induced mt-Keima targeting to mature lysosomes (**Fig. 1h, 1i**). Since Mul1ΔRing is specifically targeted to mitochondria (**Supl Fig. 1c, 1d**), over-expressed Mul1ΔRing unlikely creates a non-specific off-target effect.

13. Does pDrp1 levels change in neurons where Mul1 is knocked down?

The reviewer asked a valid question as to how Drp1 is activated in Mul1-mutant neurons. It is known that Drp1 recruitment to mitochondria and fission activity is primarily regulated by post-translational modifications. We speculate that increased cytoplasmic Ca^{2+} load activates calcineurin phosphatase which dephosphorylates Drp1 at serine 637 and thus activates Drp1 (Cereghetti, *et al.,* 2008). We added immunoblot analysis by applying an antibody against ser637-Drp1. Consistent with the literature, a suppressed phosphorylation status of Drp1 at ser637 was observed in Mul1-depleted neurons compared to control neurons expressing scr-shRNA. We added this new data in revised (**Supl Fig. 8h**).

Reference: Cereghetti, G. M. *et al.* Dephosphorylation by calcineurin regulates translocation of Drp1 to mitochondria. *Proc. Natl. Acad. Sci. USA* **105,** 15803–15808 (2008).

Minor points:

1. In Supplementary Figure 1, mitochondrial localization of the dTM1/2 mutant is still evident.

We replaced **Supl Fig. 1c** (bottom panel) with a representative image showing a typical diffused pattern of GFP-tagged Mul1ΔTM1/2.

2. How is the dominant-negative effect of Mul1-dRING explained in Figure 1e?

Mul1 has a RING finger domain with E3 ubiquitin ligase activity. To generate Mul1 loss-of-function mutant, we expressed mitochondria-targeted but RING-deleted mutant (Mul1ΔRing). Overexpressing this mutant facilitates more neurons to activate Parkin-mediated mitophagy under mild stress conditions (**Fig 1e**). We speculate that overexpressed Mul1ΔRing targets mitochondria and competes with relatively low levels of endogenous Mul1 for its interaction and

ubiquitination of mitochondrial protein Mfn2, thus interfering with Mito-ER contact and inducing mitochondrial fragmentation leading to mitophagy. We added this statement on **Page 18.**

3. A delayed mitophagic response in neurons with mitochondrial depolarization could be due to the intrinsic reliance of neurons on mitochondria to generate ATP to fuel cellular processes, including Parkin translocation and mitophagy.

We fully agree with the reviewer and included one statement on **page 4** to read:
"These findings argue for intrinsic neuronal mechanisms that can maintain and/or recover mitochondrial integrity and thus ATP homeostasis, rather than rapid elimination of dysfunctional mitochondria through Parkin-mediated mitophagy, at least in the early stages of mitochondrial stress."

4. Does dTM1/2 affect localization of Parkin to mito in AA-treated neurons?

Our analysis indicates that Mul1ΔTM1/2 displays a typical diffused pattern in neurons due to loss of the C-terminal mitochondria-targeting transmembrane domain (**Supl Fig. 1c)** and does not affect the recruitment of Parkin into mitochondria compared to control neurons under AA-treated conditions. We added this in the text on **Page 7** to read:

"As a negative control, expressing Mul1ΔTM1/2, a Mul1 mutant deficient in mitochondrial targeting, did not affect the localization of Parkin into mitochondria compared to control neurons under mild stress conditions".

5. Figure S3d is hard to interpret without knowing the relative expression levels of GFP-Mul1 and the dRING mutant. The reduced effect of dRING can be explained by a potential reduction in the expression of this construct upon introduction of wt mul1. Thus, the authors should introduce a non-specific protein as a control.

In our original data, we already co-expressed GFP and Mul1ΔRING in comparing to GFP-Mul1 and Mul1ΔRing. To further address reviewer's concern, we provided new data by adding co-expression of GFP-Sec61-β (ER membrane marker) with Mul1ΔRING **(Supl Fig. 4d)**. The new analysis indicates no significant change in percentage of neurons with mitochondrial fragmentation between groups of GFP-Mul1/Mul1ΔRING versus GFP-Sec61β/Mul1ΔRING.

We modified a statement on **page 9** to read: "While overexpressing Mul1 did not induce the biphasic change in mitochondrial morphology (**Fig. 2b-d**), fragmentation in Mul1-deficient neurons was partially reversed (*p*=0.0017) by re-introducing GFP-Mul1 but not by a control protein GFP-Sec61-β (**Supl Fig 4d**), thus highlighting the role of Mul1 in suppressing mitochondrial fragmentation in the second phase".

Reviewer #2 (Remarks to the Author):

This study by Puri et al discovers a novel mechanism underlying mitochondrial maintenance during chronic stress. The mitochondrial E3 ligase Mul1 suppresses Mfn2 to maintain ER-mito integrity, which is crucial to prevent mitophagy. Overall this is an interesting study and the experiments were well designed and performed. However, at least two key experiments are missing. Because mitophagy is a very dynamic process and cannot be simply demonstrated by co-localization of overexpressed mcherry-Parkin and CFP-mito, the authors need to show whether mitophagy kinetics is faster in Mul1 knockdown/Mfn2-overexpressing cells compared to controls. Also, does knocking down Mfn2 in Mul1 knockdown neurons rescue the mitochondrial phenotype?

We appreciate this reviewer's encouraging comments on our study. The reviewer raised many insightful suggestions which helped us to strengthen our study by adding substantial new data (see our point-by-point response below). In particularly, we expanded our study by conducting two key experiments asked by the reviewer.

(1) Assess mitophagy in Mul1 knockdown and Mfn2-overexpressing neurons.
We followed the reviewer's suggestion (also shared by reviewer 1) by using mt-Keima, a ratiometric pH-sensitive fluorescent protein that is targeted into the mitochondrial matrix. Keima has an excitation spectrum that changes according to pH. A short wavelength (458 nm) is predominant for excitation in a neutral environment, whereas a long wavelength (561 nm) is predominant in an acidic environment. When mitochondria are healthy, mt-Keima displays a low fluorescence ratio (561nm/458nm) (lysosomal red / mitochondrial green); however, when mitochondria are fragmented and engulfed by acidic lysosomes (mitophagy), mt-Keima display a strong red signal. Since mt-Keima is resistant to lysosomal proteases, it ideally allows for measurement of dynamic and cumulative mitophagy process.

We followed the well-established protocol (Bingol et al., *Nature* 2014; Katayama H et al., *Chem Biol*. 2011) to assess mitophagy by measuring relative ratio of the area of lysosomal red signal / mitochondrial green signal within the cell body, here, referred to as "**mitophagy index**". Mul1 depletion with shRNA (**Fig. 1h**) or overexpression of Mul1 loss-of-function mutant (**Fig. 1i**) enhances dynamic mitophagy in neurons. In addition, neurons with Mfn2 overexpression displayed enhanced lysosomal targeting of mt-Keima over a time course from DIV10-11 to DIV 14-15 (p<0.001) **(Fig. 4i, j)**. In contrast, expressing Mfn1 has no effect in inducing mitophagy from the same time course (p>0.05). These newly added data consistently support our conclusion that Mul1 deficiency enhances mitophagy in neurons and elevated Mfn2 activity is sufficient to mediate Mul1-deficient phenotypes- mitophagy. We also described this assay and results on **pages 7-8, and 12**.

Reference:
Bingol B et al. The mitochondrial deubiquitinase USP30 opposes parkin-mediated mitophagy. ***Nature*** 510, 370–5 (2014)

Katayama H et al. A sensitive and quantitative technique for detecting autophagic events based on lysosomal delivery. ***Chem Biol.*** 18, 1042-52 (2011)

(2) Knock down Mfn2 in Mul1-mutant neurons to rescue the mitochondrial phenotype.
To address this issue, we chose to examine the rescue of mitochondrial fragmentation with Mfn2 knock-down in Mul1-deficient neurons. Cortical neurons were transfected with Mul1 mutant Mul1ΔRing at DIV7-8, followed by a second transfection at DIV10-11 with Mfn2-shRNA-

1, Mfn2-shRNA-2, or control scr-shRNA. Mul1-targeted mitochondria were imaged at DIV14. Depleting Mfn2 in Mul1-deficient neurons effectively rescued mitochondrial fragmentation, thus establishing the role of Mfn2 in mediating the Mul1-deficient phenotype on mitochondrial fragmentation. We added this new data in **Supl Fig. 8f, 8g** and described the results on **page 11**.

In Fig. 1, the chronic stress was triggered by a low dose of AA for a few hours. However, AA should be combined with oligomycin for such treatment because membrane potential reverses without oligomycin (PMID: 26266977). The authors will need to measure the membrane potential during the treatment as well.

In the current study, we took a unique approach to address the following questions: (1) Do neurons harbor distinct mechanisms for recovery versus rapid degradation of chronically stressed mitochondria? (2) Is Parkin-mediated mitophagy the second resort for neuronal mitochondrial quality control after recovery mechanisms have failed? (3) If this is the case, does mitochondrial ubiquitin ligase 1 (Mul1), rather than cytosolic ubiquitin ligase Parkin, play an early role in maintaining neuronal mitochondrial integrity? Addressing these questions is particularly relevant to major neurodegenerative diseases associated with chronic mitochondrial stress. Thus, we chose a low dose of AA to induce chronic mitochondrial dysfunction. Our assumption is also supported by **Reviewer 1** who comments that unique mitophagic response in neurons could be due to the intrinsic reliance of neurons on mitochondria to generate ATP to fuel cellular processes.

The study cited by the reviewer (Lazarou et al., Nature 2015) reported an acute and global mitophagy induction and mitochondrial elimination in non-neuronal cell types (HeLa cells) by application of high dose of Antimycin A (4 μM) and oligomycin (10 μM) in combination with apoptosis inhibitor at different time points. Unfortunately, such a high dose (in micromole levels) of AA and oligomycin treatment is quite toxic to mature neurons maintained in our culture conditions. We previously tested the effect of different dose of AA on mitochondrial integrity by measuring mitochondrial oxygen consumption rate (OCR) in cortical neurons (Lin et al., ***Neuron*** 2017). Our study demonstrated that treatment of neurons with low dose (100 nM) of AA causes a dramatic reduction of OCR ($p<0.0001$); such a reduction could not be reversed after removing AA from the medium for 1 hour. However, this treatment is able to maintain neuronal survival, allowing us to perform various live imaging and cell biology studies.

As suggested by the reviewer, we measured mitochondrial membrane potential ($\Delta\psi_m$) with TMRE intensity following a 3-hour treatment with 100 nM AA or DMSO control. Our new data indicate a significant reduction in $\Delta\psi_m$ in the AA-treated neurons relative to DMSO control (**Supl Fig. 2**), thus supporting our assumption that a mild AA treatment is sufficient to depolarize mitochondria, thus allowing us to examine neuronal mitochondrial maintenance. We also described this new study on **page 7**.

The images in Fig. 1a-d are not consistent with the quantification in e. From looking at the images alone, Fig1 a, b, d look very similar (different from c)–all show dramatic change of Parkin from being cytosolic before treatment to punctate after treatment. The quantification in e is the % of total neurons, while a-d show only one cell. Larger images that contain multiple neurons are needed.

The reviewer raised an insightful point on the relevance of the images (**Fig. 1a-d**) and quantitative data (**Fig. 1e**). We apologize for not clarifying these. Our previous studies in mature cortical neurons demonstrated that Parkin-mediated mitophagy is only observed in a small

portion of neurons following mitochondrial depolarization (Cai et al., *Current Biology* 2012; Lin et al., *Neuron* 2017). Consistently, the Schwarz group reported that mitophagy is only observed in a small portion of axonal mitochondria following acute depolarization (Ashrafi et al., *JCB* 2014). These indicate that post-mitotic cells like neurons have unique mechanisms maintaining mitochondrial integrity, rather than rapid elimination of dysfunctional mitochondria through mitophagy.

To examine whether Mul1 deficiency enhances neuronal mitophagy, we instead measured the percentage of neurons displaying Parkin translocation under our mild depolarization condition (**Fig. 1e**). We hypothesize that neurons have an intrinsic mechanism for recovering chronically stressed mitochondria through Mul1 pathway before recruiting Parkin to damaged mitochondria. If this checkpoint mechanism fails (Mul1 deficiency), Parkin-mediated mitophagy is then activated in more percentage of neurons. While **Fig. 1a-d** show representative images of Parkin recruitment under each condition, our quantitative data (**Fig. 1e**) demonstrates more neurons with Mul1 deficiency undergoing mitophagy than in control neurons. Thus, our study suggests that Mul1 is required to suppress Parkin recruitment to neuronal mitochondria for degradation. We added the rational of these measurements on **page 7**.

To address the reviewer's concern, we also provided new data by measuring the relative intensity of Parkin signal on mitochondria and performing Pearson's correlation coefficient in Mul1 KD neurons compared to scrambled control. New data (**Fig. 1f**) indicate a higher correlation of Parkin signal on mitochondria in shRNAi-Mul1 neurons (R=0.58) relative to control neurons (R= 0.46, $p<0.05$) under mild mitochondrial stress conditions.

In response to the reviewer's first question, we also measured dynamic mitophagy by using mt-Keima to confirm the targeting of fragmented mitochondria to acidic lysosomes under Mul1 depletion with shRNA (**Fig 1h**) and overexpression of Mul1 mutant (**Fig. 1i**). These newly added data consistently support our conclusion: Mul1 deficiency enhances mitophagy in neurons.

Fig. 1f needs a wild-type control and quantification.

We provided quantitative data showing the percentage of mitochondria co-localized with LAMP1 under control (scr-shRNA) and Mul1-depletion conditions (Mul1-shRNA) (**Fig 1g**). We also measured dynamic mitophagy by using mt-Keima to confirm the targeting of fragmented mitochondria to acidic lysosomes under Mul1 depletion (**Fig 1h**) and overexpression of Mul1 loss-of-function mutant (**Fig. 1i**) in comparison with wild-type controls.

Fig. 4d needs loading control and quantification.

We added Mfn2 loading control in **Fig 4d**, which reflects an increased total Mfn2 level in cells after Mul1 knockdown. We also demonstrated that mitochondria-targeted Mfn2 was increased in Mul1-deficient neurons by measuring the Mfn2/cyto c intensity ratio in individual mitochondria distributed along dendritic processes. Expressing Mul1ΔRing significantly increases the relative Mfn2/cyto c intensity ratio ($p<0.001$) while overexpressing Mul1 reduces the ratio ($p<0.001$) relative to control neurons (**Fig. 4b, c**).

Flag-Mul1ΔRing was overexpressed in neurons with endogenous Mul1, so it is not "loss of function" as stated throughout the paper.

We referred to Flag-Mul1ΔRing as a loss-of-function mutant of Mul1. Our study consistently demonstrates that knockdown of Mul1 or expressing Mul1ΔRing in cortical neurons induced similar phenotypes, including mitochondrial fragmentation and enhanced neuronal mitophagy, as readout by mt-Keima targeting to lysosomes. Since Mul1ΔRing mutant targets mitochondria, we speculate that over-expressed Mul1ΔRing likely competes with endogenous Mul1 for either mitochondrial targeting or Mfn2 interaction. For more precise statement, we replace "neurons with Mul1 loss-of-function" with "**Mul1-deficient neurons**" throughout the text.

Fig. 5: Because Flag-Mul1ΔRing was overexpressed, an important control here is full-length Flag-Mul1 overexpression. Also, the ER-mito contacts should be examined in Mul1 knockdown cells.

The reviewer made an insightful point, which was also shared by Reviewer 3. Immuno-EM ultrastructural analysis of the ER-Mito contact is a time-consuming and resource competitive effort (NINDS EM facility). In the past three months, we collectively addressed the reviewers' concerns by expanding our immune-EM study to four new groups: including neurons expressing (1) full-length WT Mul1, (2) knocking down Mul1, (3) Mfn1, and (4) Mfn2. Our new data demonstrate that while overexpressing WT Mul1 enhances ER-Mito contact (p<0.001), expressing Mul1 Ring-deleted mutant or knocking down Mul1 significantly suppresses the ER-Mito contact (p=0.01, p<0.05, respectively). In addition, overexpressing Mfn2, but not Mfn1, also inhibits the ER-Mito contact (p<0.001). Altogether, our ultrastructural analysis consistently indicates that (1) Mul1 protects neuronal mitochondria integrity by maintaining ER-Mito contacts; (2) impaired ER-Mito contacts in Mul1-deficient neurons is through elevated Mfn2 activity. Our new immune-EM data were added in **Fig. 5d-5g** and described in text on **page 13**.

Page 11: "Altogether, our super-resolution imaging and ultrastructural analysis consistently indicate impaired ER-Mito contacts in Mul1 loss-of-function neurons, likely through elevated Mfn2 activity". This speculation should be tested by knocking down Mfn2.

The reviewer made an excellent point. Knocking down Mfn2 in iTEM approach is technically challenging because tracing transfected neurons under EM needs co-expression of GFP with siRNA constructs, which will interfere with our labeling of GFP-Sec61β as an ER marker. To address the reviewer's concern, we alternatively performed the following two experiments.

First, our new data demonstrate that expressing Mfn2, but not Mfn1, inhibits the ER-Mito contact (p<0.001), indicating that impaired ER-Mito contacts in Mul1-deficient neurons is mediated through elevated Mfn2 activity. Our new immune-EM data were added in **Fig. 5d, 5e** and described in text on **page 13**.

Second, to collectively address reviewers 2 and 3, we also tested whether Mfn2 is required to mediate the Mul1-deficient effect by knocking down Mfn2. Cortical neurons were transfected with Mul1ΔRing at DIV7-8, followed by the second transfection at DIV10-11 with Mfn2-shRNA-1, Mfn2-shRNA-2, or control scr-shRNA. Mitochondria were imaged at DIV14. Depleting Mfn2 in Mul1-deficient neurons effectively rescued mitochondrial fragmentation, thus establishing the causal role of Mfn2 in mediating Mul1-deficient phenotype. We added this new data in **Supl Fig. 8f, 8g** and described the results on **page 11**.

In summary, our current study provides multiple lines of evidence showing that Mfn2, but not Mfn1, mediates Mul1-deficient phenotypes. First, expressing Mfn2, but not Mfn1, in Mul1-deficient neurons accelerated mitochondrial fragmentation at the early stage (DIV10) (**Fig. 4a**). Second, neurons expressing Mfn2 but not Mfn1 displayed a significant increase in parkin

translocation to mitochondria at late stage (DIV14-15) (**Fig. 4g, h**). Third, neurons expressing Mfn2 but not Mfn1 displayed enhanced lysosomal targeting of mt-Keima as mitophagy indicator over a time course from DIV10-11 to DIV 14-15 (p<0.001) (**Fig. 4i, j**). Fourth, mitochondria-targeted Mfn2 was increased in Mul1-deficient neurons (**Fig. 4b, c**). Fifth, Mul1-depleted cortical neurons displayed reduced Mfn2 ubiquitination and increased Mfn2 levels (**Fig. 4d**). In addition, expressing Mfn2 but not Mfn2 (K109A), a catalytically inactive mutant, induced an early transition of mitochondria into fragmentation at DIV10 (**Fig. 4e, f**). Altogether, these results consistently support our notion that elevated Mfn2 activity, but not Mfn1, is sufficient to mediate Mul1-deficient phenotypes, thus impairing ER-Mito contacts and facilitating mitophagy.

Fig. 4b, 7b: The antibodies for ICC need to be validated, and the images need to be quantified.

We thought that validating the specificity of anti-Mfn2 antibody is more practical through knocking down Mfn2 expression. We expressed two GFP-tagged Mfn2-shRNA-1 and Mfn2-shRNA-2 and one control scr-shRNA in cortical neurons, followed by immunostaining with the anti-Mfn2 antibody (rabbit; M6319; Sigma-Aldrich) that we applied in our study. Mfn2-shRNA-transfected neurons displayed a robust reduction of Mfn2 fluorescent signals when compared to un-transfected cells within the same images, thus validating the specificity of anti-Mfn2 antibody. We added the new data in **Fig. S8a-c** and described in the text on **page 11**.

We did quantification of mitochondria-targeted Mfn2 in Mul1-deficient neurons by measuring the Mfn2/cyto c intensity ratio along dendritic processes. Expressing Mul1ΔRing significantly increases the relative Mfn2/cyto c intensity ratio (p<0.001) while overexpressing Mul1 reduces the ratio (p<0.001) relative to control neurons (**Fig. 4b, c; Supl Fig. 8d-e**).

The commercial Drp1 antibody (Cell Signaling Technology, mAb #8570) used for our study has been widely used by multiple groups to detect endogenous levels of Drp1 protein in various cells and tissues. See a total of 62 product citations on line: (https://www.cellsignal.com/products/primary-antibodies/drp1-d6c7-rabbit-mab/8570).

Does Mfn2 overexpression cause more ER-mito contacts and faster mitophagy kinetics?

Our new data consistently demonstrate that overexpressing Mfn2 significantly inhibits the ER-Mito contact (p<0.001). As a control, overexpressing Mfn1 has no significant effect on the ER-Mito contact (**Fig. 5d, 5e**).

At the suggestion of the reviewer, we further measured the mitophagy process by using mt-Keima suggested by Reviewer 1. Cortical neurons were co-transfected at DIV7 with mt-Keima and Myc-tagged Mfn2 or Mfn1, followed by live-cell imaging at DIV10-11 or DIV14-15 (**Fig. 4i, j**). Neurons with Mfn2 overexpression displayed enhanced lysosomal targeting of mt-Keima over a time course from DIV10-11 to DIV 14-15 (p<0.001). In contrast, expressing Mfn1 has no such effect in inducing mitophagy from the same time course (p>0.05). Thus, together with our data showing (1) mitochondria-targeted Mfn2 is increased in Mul1-deficient neurons (**Fig. 4b, c**); (2) Mul1-depleted cortical neurons displayed reduced Mfn2 ubiquitination and increased Mfn2 levels **(Fig. 4d)**; (3) expressing Mfn2 but not Mfn1 (K109A), a catalytically inactive mutant (**Fig. 4e, f**); induced an early transition of mitochondria into fragmentation at DIV10; (4) expressing Mfn2, but not Mfn1, robustly triggered mitochondrial fragmentation and induced Parkin recruitment at the late stage (DIV14-15) (**Fig. 4g, h**), these results consistently suggest that elevated Mfn2 activity is sufficient to mediate Mul1-deficient phenotypes, thus facilitating mitophagy.

Reviewer #3 (Remarks to the Author):

In this manuscript, the authors reveal a Mul1-Mfn2 signaling pathway that maintains neuronal mitochondrial integrity under mild stress conditions. They show that loss of Mul1 increases the level of Mfn2, which acts as an ER-Mito tethering antagonist, impairing mitochondrial bioenergetics and Ca2+ homeostasis. They further show that reduced ER-Mito contact leads to increased cytoplasmic Ca2+, activating calcineurin and inducing Drp1-dependent mitochondrial division and Parkin-mediated mitophagy. Interestingly, expressing ER-Mito tethering protein PTPIP51 suppresses Parkin-mediated mitophagy. The authors propose that the Mul1-Mfn2 pathway plays a checkpoint role in maintaining mitochondrial integrity and/or recovering stressed mitochondria before Parkin-mediated mitophagy is activated. Overall, the study is well performed, and the conclusion, if further strengthened, will add significantly to our understanding of mitochondrial protection under stress.

We are encouraged by these positive comments on our study. The reviewer raised many insightful suggestions which have helped us to strengthen our manuscript by substantially expanding our study.

Major Comments:

1. The causal role of Mfn2 in mediating Mul1 loss of function (lof) effect on mito-ER contact is not established. Effect of mfn2 RNAi in rescuing Mul1 lof effect should be tested.

The reviewer provided an insightful suggestion. Immuno-EM based ultrastructural analysis of the ER-Mito contact is a time-consuming and resource competitive effort (NINDS EM facility). In the past three months, we collectively addressed all three reviewers' comments on this ER-Mito issue. We expanded our immune-EM study by adding four new groups: including neurons expressing (1) full-length Mul1, (2) Mul1-shRNA, (3) Mfn1, and (4) Mfn2. Our new data demonstrate that while overexpressing Mul1 enhances ER-Mito contact (p<0.001), expressing Mul1 Ring-deleting mutant or knocking down Mul1 significantly suppresses the ER-Mito contact (p=0.01, p<0.05, respectively). In addition, overexpressing Mfn2, but not Mfn1, also inhibits the ER-Mito contact (p<0.001). Altogether, our ultrastructural analysis consistently indicates that (1) Mul1 protects neuronal mitochondria integrity by maintaining ER-Mito contacts; (2) impaired ER-Mito contacts in Mul1-deficient neurons is through elevated Mfn2 activity. Our new immune-EM data were added in revised **Fig 5d-5g** and described in text on **page 13.**

In addition, we examined the role of Mfn2 in mediating the Mul1-deficient effect using Mfn2 siRNA. We chose to examine the rescue of mitochondrial fragmentation by knocking down Mfn2 in Mul1-deficient neurons. Cortical neurons were transfected with Mul1ΔRing at DIV7-8, followed by the second transfection at DIV10-11 with Mfn2-shRNA-1, Mfn2-shRNA-2, or control scr-shRNA. Mitochondria were imaged at DIV14. Depleting Mfn2 in Mul1-deficeint neurons effectively rescued mitochondrial fragmentation, thus establishing the causal role of Mfn2 in mediating Mul1-deficient phenotype on mitochondrial fragmentation. We added this new data in **Supl Fig. 8f-8g** and described the results on **page 11**.

2. Although mito-ER contact is affected and mito-Ca2+ homeostasis is altered, whether mito-ER Ca2+ is causally involved in Mul1 lof effect will require further study. Does genetic manipulation of mito-ER Ca2+ transfer by IP3R/VDAC/MCU have any effect?

The reviewer asked a valid question. The Ca^{2} transfer from the ER to mitochondria is through a trimeric complex between IP3R, a Ca^{2+} release channel at the ER membrane, VDAC1, a

channel located at the outer mitochondrial membrane, and Grp75, a protein that bridges interaction between IP3R and VDAC1. Ca^{2+} enters the mitochondrial intermembrane space through the IP3R/Grp75/VDAC1 channel complex and then enters the mitochondrial matrix through the mitochondrial calcium uniporter (MCU). Genetic or molecular manipulation of the IP3R/VDAC/MCU axis affects mitochondrial integrity by altering ER-Mito Ca^{2+} transfer and homeostasis. For example, blocking the IP3R function inhibited IP3R-mediated Ca^{2+} release from the ER, resulting in mitochondrial depolarization (Higo et al., *Neuron* 2010). Interaction of VDAC1 with phosphorylated tau and Aβ disrupts the IP3R/Grp75/VDAC1 channel complex leading to mitochondrial dysfunction in Alzheimer's disease (Manczak and Reddy, *Hum. Mol. Genet.* 2012). In $MCU^{-/-}$ CD1 mouse strain, mitochondrial Ca^{2+} uptake is impaired and mitochondrial ATP production is reduced (Pan et al., *Nat. Cell Biol.* 2013). Using STED super-resolution microscopy and immuno-EM analysis combined with imaging of mitochondrial Ca^{2+} uptake, our current study provided the first line of evidence in mature neurons that Mul1 plays a critical role in sustaining ER-Mito contacts. Consistently, expressing an ER-Mito anchoring protein PTPIP51 in Mul1-deficient neurons enhances IP3R-induced Ca^{2+} transfer from ER to mitochondria, rescues mitochondrial fragmentation, and suppresses Parkin-mediated mitophagy. Thus, maintaining neuronal ER-Mito contacts is critical to suppressing mitochondrial fragmentation and mitophagy. This notion is consistent with a recent report showing that reducing ER-Mito contact accelerates mitophagy in response to mitochondrial depolarization (McLelland et al., *Elife* 2018). Our previous study also demonstrated that mitophagy is significantly delayed in mature neurons in response to chronic or mild mitochondrial stress (Lin et al., *Neuron* 2017). Thus, Mul1 ideally acts as an early checkpoint of neuronal mitochondrial quality control by sustaining ER-Mito contacts.

Together, these studies indicate that genetic and molecular manipulation of IP3R/VDAC/MCU (mito-ER Ca^{2+} transfer) axis affect mitochondrial integrity. Our revision already includes 10 full-page figures and 8 full-page supplemental figures; genetic manipulation of IP3R/VDAC/MCU complexes is beyond the scope of the current study, which is focused on neuronal mitochondrial quality control in response to mild stress. Instead, we expanded discussion on this issue on **Pages 21-22**.

3. The role of Mfn in Mito-ER contact is controversial. Recent studies suggest that Mfn may be differentially involved in regulating different types of mito-ER contacts: the "narrow" vs. "wide" contacts. The authors are recommended to carefully characterize their EM pictures to assess the effect of Mul1 lof on these different types of contacts.

The reviewer made an excellent suggestion. We recharacterized EM-Mito contact and found Mul1 mainly impacted the "narrow" ER-Mito contact (less than 12 nm). To further address this issue, we expanded our immune-EM study by adding two new groups, including neurons overexpressing Mfn1 or Mfn2. Our new data demonstrate that overexpressing Mfn2, but not Mfn1, inhibits the "narrow" ER-Mito contact ($p<0.001$), suggesting that impaired ER-Mito contacts in Mul1 loss-of-function neurons is through elevated Mfn2 activity. Our new immune-EM data were added in revised **Fig. 5d and 5e** and described on **page 13**.

Altered ER-Mito contacts by elevated Mfn2 but not Mfn1 is further supported by our newly added study of the dynamic mitophagy process using mt-Keima suggested by **Reviewer 1**. Cortical neurons were co-transfected at DIV7 with mt-Keima and Myc-tagged Mfn2 or Mfn1, followed by live-cell imaging at DIV10-11 or DIV14-15 (**Fig. 4i, j**). Neurons with Mfn2 overexpression displayed enhanced lysosomal targeting of mt-Keima over a time course from DIV10-11 to DIV 14-15 ($p<0.001$). In contrast, expressing Mfn1 has no such effect in inducing mitophagy from the same time course ($p>0.05$). Thus, together with our data show that (1)

mitochondria-targeted Mfn2 is increased in Mul1-deficient neurons (**Fig. 4b, c**); (2) Mul1-depleted neurons displayed reduced Mfn2 ubiquitination and increased Mfn2 levels **(Fig. 4d)**; (3) expressing Mfn2 but not Mfn2 (K109A), a catalytically inactive mutant (**Fig. 4e, 4f**); induced an early transition of mitochondria into fragmentation at DIV10; (4) expressing Mfn2, but not Mfn1, robustly triggered mitochondrial fragmentation and induced Parkin recruitment at the late stage (DIV14-15) (**Fig. 4g, 4h**), and (5) overexpressing Mfn2, but not Mfn1, inhibits the "narrow" ER-Mito contact ($p<0.001$) (**Fig. 5d, 5e**), these results consistently suggest that elevated Mfn2 activity is sufficient to mediate Mul1-deficient phenotypes.

4. The analysis of effects of Mul1 on neuronal function is limited to mitochondrial morphology, membrane potential, and ATP production. More analysis on neuronal communication or survival may strengthen the physiological significance of this study.

Cortical neurons from *parkin* KO mice showed no significant changes in mitochondrial membrane potential and neuronal morphology as compared with wild-type neurons (Yun et al., *eLife* 2014). However, Mul1 knockdown in *parkin* KO neurons in the early stage (DIV10-11) resulted in a loss of $\Delta\psi_m$ and an increase in the number of neurons with dendritic and axonal fragmentation and retraction, a key indicator of early neurodegeneration.

To follow the reviewer's suggestion, we also provided new quantitative analysis of morphology and complexity of dendritic arborization, a key determinant of neural connectivity and communication. One of the well-established measurements is Sholl analysis of dendritic arborization pattern. We provided Sholl analysis in neurons expressing scr-shRNA vs Mul1-shRNA (**Supl Fig. 7**). Our results indicate a significant reduction in dendritic arborization pattern in Mul1-deficient neurons at DIV14-15 compared to control. This new data supports our conclusion that Mul1 deficiency affects mitochondrial integrity which impairs neuronal cell connectivity and survival. We expanded discussion on **Page 10.**

5. In other settings, increased mito-ER contacts and increased Ca or lipid transfer from ER to mitochondria has been implicated in Pink1/parkin lof. This should be specifically discussed, especially considering that Mul and Pink1/Parkin has been proposed by the authors to act in parallel pathways to preserve mitochondrial function through the same target Mfn.

The role of Pink1/parkin in regulating ER-mitochondrial interaction has been controversial. Depletion of Parkin causes a decrease in ER-Mito signaling resulting in reduced mitochondrial Ca^{2+} uptake and ATP production (Cali et al., 2013). A reduced tethering between ER and mitochondria was also observed in Parkin-deficient *Drosophila* cells and Parkin mutant human fibroblasts (Basso et al., 2018). However, these observations were challenged by Gautier et al (2016) reporting an increase in ER-Mito association in fibroblasts derived from PARK2 KO mice. One explanation for these controversial findings could be compensatory mechanism(s). In our previous study (Yun et al., *eLife* 2014), we found that Mul1 and Pink1/ Parkin act in parallel pathways to preserve mitochondrial integrity through Mfn2. Our current study further indicates that Mul1 acts as the first checkpoint to maintain mitochondrial integrity by regulating Mfn2 and sustaining ER-Mito contacts. If the Mul1-Mfn2 pathway fails, as the second resort for mitochondrial quality control, the cytosolic Parkin is recruited to mitochondria to initiate mitophagy. These findings argue for intrinsic neuronal mechanisms that can maintain mitochondrial integrity and thus ATP homeostasis in the early stages of mitochondrial stress, rather than rapid elimination of dysfunctional mitochondria through Parkin-mediated mitophagy. As suggested by the reviewer, we expanded our discussion on this issue on **pages 18-19.**

Minor comments:

1. Specificity of ShRNA studies should be demonstrated by rescue with shRNA-resistant cDNA.

At suggested by the reviewer, we generated an shRNA-resistant Mul1 mutant (Mul1*) by substituting eight third-code nucleotides in Mul1-shRNA-targeting sequence (A210T, T213A, G216A, G219A, T222A, T225A, A228G, and A231G) without changing amino acid sequence. While Mul1-shRNA suppresses endogenous Mul1 and significantly deplete exogenous Flag-Mul1 (p<0.001), it fails to suppress silent mutant Mul1* (p=0.68), thus indicating an shRNA-resistant Mul1 (Mul1*). We added this data in **Supl Fig. 5a, 5b**.

We tested the specificity of shRNA knockdown with the shRNA-resistant Mul1* using more robust assays: mitochondrial fragmentation and loss of membrane potential. Our newly added data demonstrate that mitochondrial fragmentation (**Supl Fig. 5c, 5d)** and mitochondrial depolarization (**Supl Fig. 5e, 5f)** in Mul1-deficient neurons (Mul1-shRNA) can be effectively rescued by co-expressing Mul1*. The rescue of two Mul1-depelted phenotypes consistently indicate the specificity of Mul1-shRNA approach, thus excluding an off-target effect. We described the results on **page 9**.

2. Mechanism and specificity of Mul1 regulation of Mfn2. Is Mfn1 not regulated the same way as Mfn2?

To address the reviewer's question, we used multiple lines of analysis to dissect which Mfn isoform is regulated by Mul1, thus mediating Mul1's role in neuronal mitochondrial quality control. **First**, expressing Mfn2, but not Mfn1, in Mul1-deficient neurons accelerated mitochondrial fragmentation at the early stage (DIV10) (**Fig. 4a**). **Second**, neurons with over-expression of Mfn2, but not Mfn1, displayed a significant increase in parkin translocation to mitochondria at late stage (DIV14-15) **(Fig. 4g, h). Third**, neurons expressing Mfn2, but not Mfn1, displayed enhanced lysosomal targeting of mt-Keima as mitophagy indicator over a time course from DIV10-11 to DIV 14-15 (p<0.001) **(Fig. 4i, j).** Thus, together with our data showing that **(1)** mitochondria-targeted Mfn2 is increased in Mul1 loss-of-function neurons **(Fig. 4b, c)**; **(2)** Mul1-depleted cortical neurons displayed reduced Mfn2 ubiquitination and increased Mfn2 levels (**Fig. 4d**); **(3)** expressing Mfn2 but not Mfn2 (K109A), a catalytically inactive mutant **(Fig. 4e, f)**; induced an early transition of mitochondria into fragmentation at DIV10, these new results consistently support our notion that elevated Mfn2 activity, but not Mfn1, is sufficient to mediate Mul1-deficient phenotypes, thus facilitating mitophagy.

Although Mfn1 and Mfn2 belong to the family of ubiquitous transmembrane GTPases, Mfn1 and Mfn2 regulate mitochondrial fusion albeit at different stages (Chen et al., *J. Cell Biol*. 2003; Santel et al., *J. Cell Sci.* 2003). Mfn1 has been shown to mediate mitochondrial docking and fusion more efficiently than Mfn2, probably due to its higher GTPase activity (Ishihara et al. *J. Cell Sci.* 2004). These findings indicate that there is a difference in mitochondrial fusogenic properties between Mfn1 and Mfn2. Furthermore, Mfn2, but not Mfn1, has been shown to be present in the ER and also enriched at the ER-Mito interface regulating Ca^{2+} and lipid transfer from ER to mitochondria (de Brito et al. *Nature* 2008; Filadi et al. *Proc. Natl. Acad. Sci. USA* 2015). Interestingly, Mfn2 has been shown to interact with multiple proteins which regulate Mfn2 activity and modulate ER-mitochondrial tethering (Cerqua et al., *EMBO Rep.* 2010, Filadi et al., Cell Rep. 2016). Together, these results indicate a non-redundant or distinct role of Mfn2 and Mfn1. We have expanded discussion on our findings showing elevated Mfn2 activity, but not Mfn1, is sufficient to mediate Mul1-deficient phenotypes, thus facilitating mitophagy on **page 18**.

Reviewers' comments:

Reviewer #1 (Remarks to the Author):

In this revised manuscript, the authors added additional dynamic imaging data that support the overall conclusion that Mul1 functions to reduce mitophagy in neurons and depletion of Mul1 causes a bi-phasic change in mitochondrial morphology explained by increased Mfn2 abundance and activation of Drp1. Additional control data to address the specificity of Mul1 shRNA is also provided in mitochondrial morphology and membrane potential endpoints. The biochemistry data on Mfn2 ubiquitination and Drp1 protein localization look convincing, so do the Seahorse data. Though the newly added data confirm these overall conclusions, the authors did not address some of the crucial controls raised in the first review, as outlined below:

• Multiple figures remain lacking proper controls that set the baseline in the experiment thus making it hard to judge the effects of overexpression/knockdown: Figure 1e, control is not shown for oe constructs, Mul1 and dRING-Mul1. In Figure 1i, 4c, 5b, 6, FLAG only is not an appropriate control, a better control would be to express a FLAG-tagged non-specific protein. In Figure 4a, scr-shRNA or Mul1-shRNA only (or the Mul1 cDNA-only) conditions are not performed so we cannot judge if the effect of Mfn2 oe is significant.

• In Figure 2, the statistical analysis of ANOVA for the effect of culture time within a transfection group (2c-comparison of black-blue bars) and the effect of transfection (2e-comparison within blue bars or black bars) are shown separately. For multiple comparisons, statistical analysis cannot be separated into selected groups (which defeats the purpose of multiple comparison testing). Please combine figures 2c and e, and indicate the multiple-test statistics within the same graph, performed a single time on the whole data set.
• The authors did not indicate the analysis and sample selection was performed blindly, which was a major concern in the first review (point #4).
• A non-tagged shRNA system could be used to address the effects of Mul1 knockdown in the imaging experiments with calcium reporters. Calcium imaging experiments, though elegant, remain based solely on overexpression and without proper controls (e.g. "mock" is not a proper control in 8a).
• Identification of ER-mito contact sites depends on overexpression of GFP-Sec61b, which may overestimate the abundance of these structures.
• In Figure 1g, only 20 transfected neurons were scored over 3 experiments to calculate the percent of mito/LAMP1 colocalization. How were these neurons chosen for imaging in a given transfected-dish?
Minor points:
• How is endogenous Mul1 visualized in Sup Fig 5a where FLAG-Mul1 was transfected?
• "GFP" is missing from the Mul1-dRING mutant in Figure 3g.

Comments on the experiments done to address reviewer#2:

(1) Assess mitophagy in Mul1 knockdown and Mfn2-overexpressing neurons.

The authors convincingly showed Mul1 shRNA increases mitophagy index (Fig 1h).

Though "FLAG-only" is not an appropriate control, the overexpression experiment in Fig 1i also suggests increased mitophagy with dRING MUL1 mutant, confirming the knockdown experiment.

Regarding the effect of Mfn overexpression on mitophagy (Fig 4 i,j): The authors did not include a non-specific control e.g. GFP in Fig 4j. Thus, it is not clear if Mfn1 or Mfn2 by themselves change the basal mitophagy levels in this experiment. In addition, potential expression level differences between Mfn1 and Mfn2 in transfected neurons are not documented. Thus, the proposed increase in mitophagy

with Mfn2 overexpression, but not with Mfn1 overexpression, needs further verification.

(2) Knockdown Mfn2 in Mul1-mutant neurons to rescue the mitochondrial phenotype.

The authors did not perform a detailed analysis of mitochondrial morphology (e.g. measuring mitochondrial size, aspect ratio as in Figure 2), instead analyzed "% of neurons displaying mitochondrial fragmentation". Since this latter quantification can be subjective, the data and quantification remain preliminary.

Reviewer #3 (Remarks to the Author):

The authors have adequately addressed this reviewer's concerns.

Reviewer #1 (Remarks to the Author):

In this revised manuscript, the authors added additional dynamic imaging data that support the overall conclusion that Mul1 functions to reduce mitophagy in neurons and depletion of Mul1 causes a bi-phasic change in mitochondrial morphology explained by increased Mfn2 abundance and activation of Drp1. Additional control data to address the specificity of Mul1 shRNA is also provided in mitochondrial morphology and membrane potential endpoints. The biochemistry data on Mfn2 ubiquitination and Drp1 protein localization look convincing, so do the Seahorse data. Though the newly added data confirm these overall conclusions, the authors did not address some of the crucial controls raised in the first review, as outlined below:

We appreciate the reviewer's encouraging comments that our most added data in the previous revision, including dynamic mitophagy, mitochondrial morphological bi-phasic change, the specificity of Mul1-shRNA, Mfn2 ubiquitination, Drp1 protein localization, and Seahorse data, confirmed our overall conclusions. In the current revision, we provided new data to address the remaining concerns on some controls.

Multiple figures remain lacking proper controls that set the baseline in the experiment thus making it hard to judge the effects of overexpression/knockdown:
Figure 1e, control is not shown for oe constructs, Mul1 and dRING-Mul1

This is an excellent suggestion to improve **Fig. 1e**. We added GFP control under both DMSO and antimycin conditions as the set of baselines and directly compared the baselines of Parkin translocation with neurons expressing GFP-dRING-Mul1, GFP+Mul1-shRNA, or GFP-Mul1. The average percentage of neurons displaying Parkin recruitment is 37.06 ± 2.47% (Mul1-shRNA, p<0.001) and 24.77 ± 2.21% (Mul1ΔRing, p<0.01), respectively, relative to control neurons (GFP: 14.84 ± 1.56%) following mild stress. Conversely, elevated Mul1 expression suppresses Parkin translocation (5.54 ± 0.67%, p<0.05) (**Fig. 1e**). We added these data in revised **Fig. 1e** and presented the baseline images in **Supl Fig. 2c**. We also described the data on **page 7**.

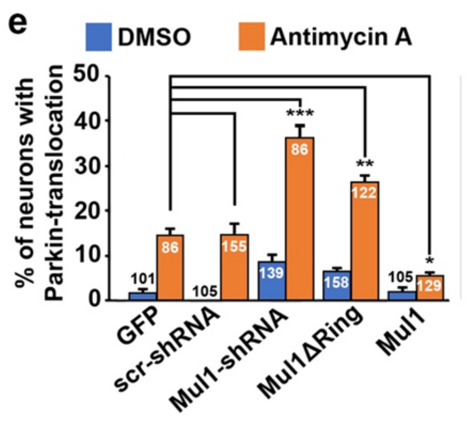

In Figure 1i, 4c, 5b, 6, FLAG only is not an appropriate control, a better control would be to express a FLAG-tagged non-specific protein.

For these studies, we aimed to directly compare effects of Flag-Mul1 and its loss-of-function mutant Flag-ΔRing in mitophagy dynamics (mt-Keima) (Figure 1i), Mfn2 levels (Figure 4c), ER-Mito contact (Figure 5b), and mitochondrial Ca^{2+} uptake (Figure 6). Our multi-phenotypic data consistently demonstrate that neurons expressing Flag-ΔRing displayed a robust increase in mitophagy dynamics and Mfn2 staining, and a reduction in ER-Mito contact and Ca^{2+} uptake, relatively to neurons expressing either Flag alone as a baseline or wild-type Mul1.

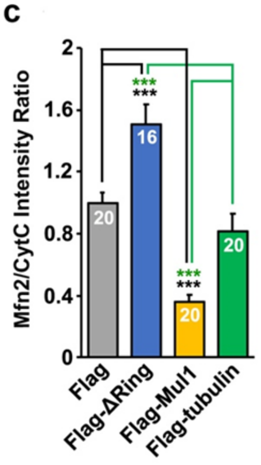

In addition, we further examined ER-Mito contact in neurons expressing various constructs including Flag alone as a baseline, Flag-Mul1, Flag-Mul1ΔRing, Myc alone, Myc-Mfn2, Myc-Mfn1, scr-shRNA and Mul1-shRNA (Figures 5e, 5g). Our super-resolution imaging and ultrastructural analysis consistently indicate that (1) Mul1 protects neuronal mitochondria integrity by maintaining ER-Mito contacts; (2) impaired ER-Mito contacts in Mul1-deficient neurons is mediated through elevated Mfn2 but not Mfn1 overexpression.

Expressing Flag-tagged non-specific proteins as an additional control seems redundant. However, we followed the reviewer's suggestion by adding FLAG-tubulin control in Mfn2 staining data (revised **Fig. 4c**). While expressing Flag-tubulin has no significant change compared with Flag alone, expressing Flag-Mul1 significantly enhances the Mfn2/CytC intensity ratio (p<0.001), and conversely expressing Flag-Mul1ΔRing reduces the ratio (p<0.001) relative to control neurons expressing either Flag alone or Flag-tubulin as the baseline. We described the data in the Figure legend on **page 34**.

In Figure 4a, scr-shRNA or Mul1-shRNA only (or the Mul1 cDNA-only) conditions are not performed so we cannot judge if the effect of Mfn2 oe is significant.

At the suggestion by the reviewer, we included new control data showing the percentage of neurons with mitochondrial fragmentation when expressing only one construct including GFP, scr-shRNA, Mul1-shRNA, or GFP-Mul1 (see blue bars in the revised **Fig. 4a**). These newly added controls support our conclusion that Mfn2, but not Mfn1, selectively accelerated mitochondrial fragmentation in Mul1-deficient neurons even at an early stage (DIV10). We described the data in the Figure legend on **page 34**.

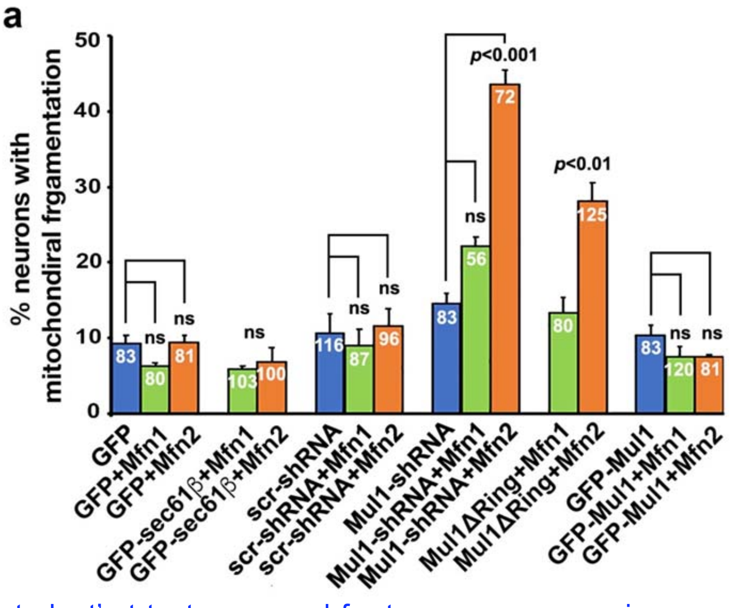

(Note that unpaired student's t test was used for two group comparison and Ordinary one-way ANOVA with Dunnet's Multiple Comparison Test was used for three group comparison).

In Figure 2, the statistical analysis of ANOVA for the effect of culture time within a transfection group (2c-comparison of black-blue bars) and the effect of transfection (2e-comparison within

blue bars or black bars) are shown separately. For multiple comparisons, statistical analysis cannot be separated into selected groups (which defeats the purpose of multiple comparison testing). Please combine figures 2c and e and indicate the multiple-test statistics within the same graph, performed a single time on the whole data set.

At the suggestion by the reviewer, we combined Figures 2c and 2e for the multiple-test statistics within the same bar graphs (revised **Fig. 2c and 2d**). Two-way ANOVA with Sidak's multiple comparison test was used for comparing two-time phase groups (blue bar DIV10-11 vs black bar DIV14-15) within each transfection condition (red ***: $p<0.001$; red **: $p<0.01$). Two-way ANOVA with Holm-Sidak's multiple comparison test was used for comparing GFP control at DIV10-11 with various transfection conditions across phase I group (DIV10-11, blue bars) (blue ***: $p<0.001$; blue *: $p<0.05$), or comparing GFP control at DIV14-15 with other transfection conditions across phase II group (DIV14-15) (black bars and black ***: $p<0.001$; black **: $p<0.01$). Also see revised Figure legend on **page 33**.

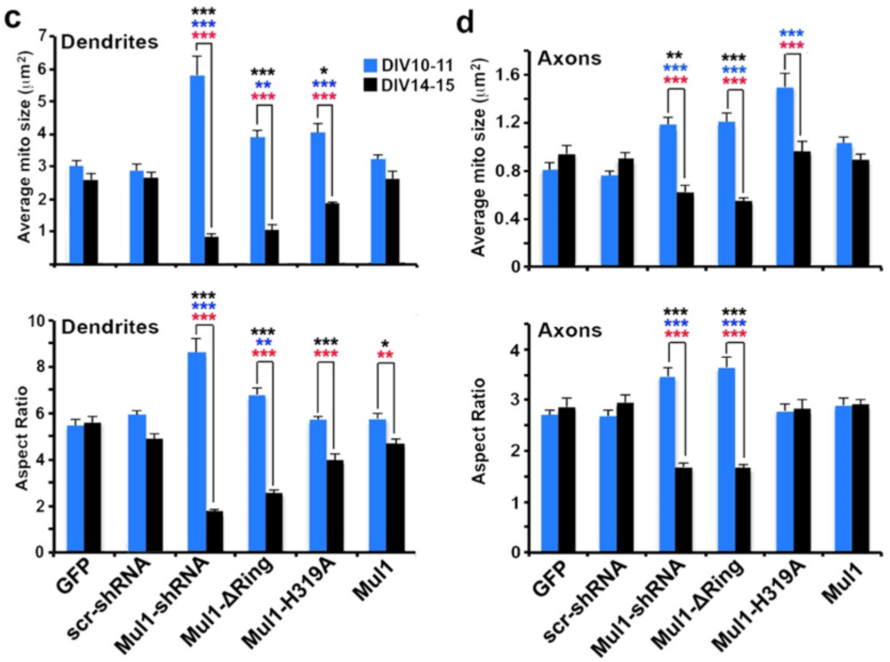

The authors did not indicate the analysis and sample selection was performed blindly, which was a major concern in the first review (point #4).

We added statement in Fig. 1 legend (**page 32**): To limit potential variations caused by unblinded selection, all transfected neurons in low-density culture (0.25 million per 10 cm^2) were selected for analysis of percentage of neurons displaying Parkin translocation. Total neuron number is from 86-158 for each transfection condition. Enhanced Parkin translocation was consistently observed in neurons with Mul1-depletion (Mul1-shRNA) and Mul1-deficient (Mul1ΔRing) (**Fig. 1e**).

Instead of directing counting neurons with Parkin translocation, we also performed Pearson's correlation coefficient by measuring the relative intensity of Parkin signal on mitochondria in Mul1 knockdown neurons compared to scrambled control. Mul1 deficiency facilitates more neurons to recruit cytosolic Parkin onto stressed mitochondria (**Fig. 1e**), resulting in a higher correlation of Parkin signal on mitochondria (**Fig. 1f**) under stress conditions. We clarified this in the figure legend on **page 32**.

A non-tagged shRNA system could be used to address the effects of Mul1 knockdown in the imaging experiments with calcium reporters. Calcium imaging experiments, though elegant, remain based solely on overexpression and without proper controls (e.g. "mock" is not a proper control in 8a).

In our first revision, we argued that it is technically challenging to trace Mul1 knockdown neurons expressing GFP-tagged shRNA because we applied a genetically encoded FRET-based ratiometric probe (4mtD3cpv) to examine mitochondrial Ca^{2+} levels. The GFP excitation/emission wavelengths (488/509 nm) interfere with the excitation/emission wavelengths (458/525 nm) of these two calcium probes.

In the current revision, we followed the reviewer's excellent suggestion by constructing a non-tagged Mul1-shRNA for this specific calcium imaging experiment. New added data (**Supplemental Fig 9**) consistently support our conclusion that neurons with Mul1 depletion display reduced mitochondrial Ca^{2+} uptake from the ER, reflecting an impaired ER-Mito interplay.

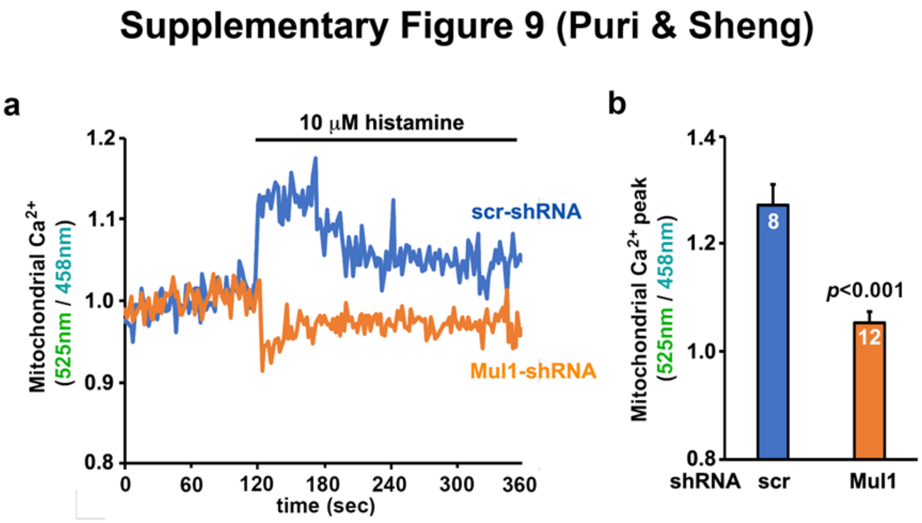

Supplementary Fig. 9 Mul1 depletion reduces mitochondrial Ca^{2+} uptake from the ER. Representative traces (**a**) and quantitative analysis of the average maximal Ca^{2+} peak (**b**) showing impaired mitochondrial Ca^{2+} uptake from the ER in Mul1-deficient neurons. Cortical neurons were co-transfected at DIV7-8 with pcDNA-4mtD3cpv and non-tagged scr or Mul1-shRNA, followed by live imaging at DIV10-11. The emission spectra of the ratiometric probe (525nm/458nm) were obtained by exciting 405nm following stimulation with 10 μM histamine. Note that Mul1-deficient neurons display a significant reduction in mitochondrial Ca^{2+} peak following Ca^{2+} release from the ER. Data were analyzed from 2-3 live neurons per field per trial and total number of trials are indicated in the bars and expressed as mean ± SEM. Unpaired Student's *t* test was used for comparing two groups.

Identification of ER-mito contact sites depends on overexpression of GFP-Sec61b, which may overestimate the abundance of these structures.

It is technically challenging to label ER in neurons followed by quantitative ultrastructural analysis of ER-Mito contacts. To limit trial-to-trial variation caused by GFP-Sec61b overexpression, we combined super-resolution images using dual-color 3D-STED nanoscopy and immuno-transmission electron microscopy (ITEM). We performed comprehensive analysis

aiming at directly comparing the relative ER-Mito contacts between Mul1 wild-type vs deficient neurons and between Mfn1 vs Mfn2 expressing neurons (Fig 5e, g). In addition, impaired ER-Mito contact was further confirmed by reduced mitochondrial Ca^{2+} uptake (Fig 6), functionally reflecting an impaired ER-Mito interplay.

In Figure 1g, only 20 transfected neurons were scored over 3 experiments to calculate the percent of mito/LAMP1 colocalization. How were these neurons chosen for imaging in a given transfected-dish?

To visualize individual neurons with their long processes, we maintained low-density neuronal cultures (0.25 million per 10 cm^2). Moreover, neurons were co-transfected using calcium phosphate method, which has low transfection efficiency but less toxic to neurons compared to high-efficient transfection reagents. Thus, total number of double-transfected neurons in each culture dish is relatively low. We chose 6-8 transfected neurons per dish per trial for each condition. We clarified these in the figure legend on **page 32**.

Minor points:

How is endogenous Mul1 visualized in Sup Fig 5a where FLAG-Mul1 was transfected?

We applied a long Flag-tag (~7Kd longer) construct and did immunoblot with an anti-Mul1 antibody on the same membrane. Given that the expression level of Flag-Mul1 is robustly higher than the endogenous one, showing both on one blot would result in over saturation of Flag-Mul1 signal density. To avoid signal saturation, we separated Flag-Mul1 blot from endogenous Mul1 blot. We clarified this in the figure legend on **page 40**.

"GFP" is missing from the Mul1-dRING mutant in Figure 3g.

We added GFP-tagged accordingly.

Reviewer #2:

(1) Assess mitophagy in Mul1 knockdown and Mfn2-overexpressing neurons.
The authors convincingly showed Mul1 shRNA increases mitophagy index (Fig 1h). Though "FLAG-only" is not an appropriate control, the overexpression experiment in Fig 1i also suggests increased mitophagy with dRING MUL1 mutant, confirming the knockdown experiment.

Regarding the effect of Mfn overexpression on mitophagy (Fig 4 i,j): The authors did not include a non-specific control e.g. GFP in Fig 4j. Thus, it is not clear if Mfn1 or Mfn2 by themselves change the basal mitophagy levels in this experiment. In addition, potential expression level differences between Mfn1 and Mfn2 in transfected neurons are not documented. Thus, the proposed increase in mitophagy with Mfn2 overexpression, but not with Mfn1 overexpression, needs further verification.

GFP, that was suggested by the reviewer, could not be used in the live imaging of mt-Keima assay, in which both red and green channels were used to monitor mitophagy dynamics. Instead, we used Myc vector alone for transfection control. We assessed "Mitophagy Index" in **Fig. 4j** by measuring the ratio of the area of 561nm/458nm (lysosomal signal red vs. mitochondrial signal green) that was further normalized to control neurons expressing Myc alone (Mitophagy Index was set to 1). We clarified normalized "Mitophagy Index" to neurons expressing Myc-tag control in the figure legend on **page 35**.

To limit variation of Mfn1 vs Mfn2 expression levels, we randomly selected 32-35 transfected neurons in low-density cultures from three experiments in each condition. Our observations are consistent in three repeats: neurons overexpressing Myc-Mfn2, but not Myc-Mfn1, displayed enhanced lysosomal targeting of mt-Keima over a time course from DIV10-11 to DIV 14-15. This can be reflected by relatively small standard error of mean (SEM) shown in **Fig. 4j**.

To further convince the reviewer, we provide here two pairs of Mfn1 and Mfn2 expression in transfected neurons at DIV10-11, which show the similar level of expression.

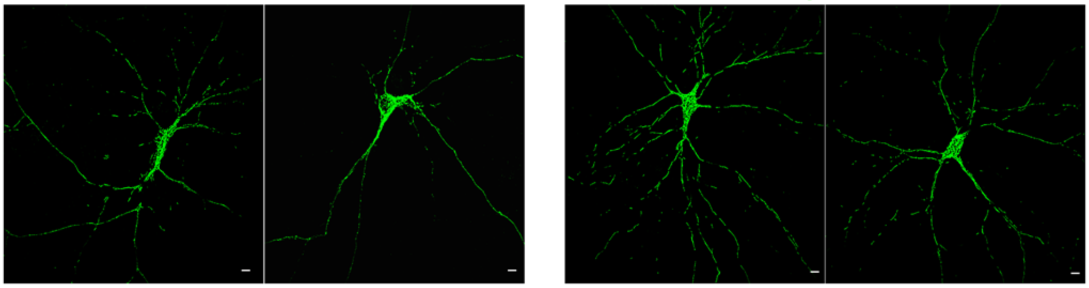

(2) Knockdown Mfn2 in Mul1-mutant neurons to rescue the mitochondrial phenotype.
The authors did not perform a detailed analysis of mitochondrial morphology (e.g. measuring mitochondrial size, aspect ratio as in Figure 2), instead analyzed "% of neurons displaying mitochondrial fragmentation". Since this latter quantification can be subjective, the data and quantification remain preliminary.

At the suggestion by the reviewer, we performed a detailed analysis of mitochondrial morphology by measuring mitochondrial size and aspect ratio in Mul1-mutant neurons with Mfn2

knockdown. Newly added data consistently demonstrate that depleting Mfn2 using two sets of Mfn2-shRNA #1 an #2 in Mul1-deficient neurons rescues mitochondrial fragmentation (**Supl Fig. 8g**) and mitochondrial size and aspect ratio (**Supl Fig. 8h**). We described the data in the test on **page 11** and in the figure legend on **page 42**.

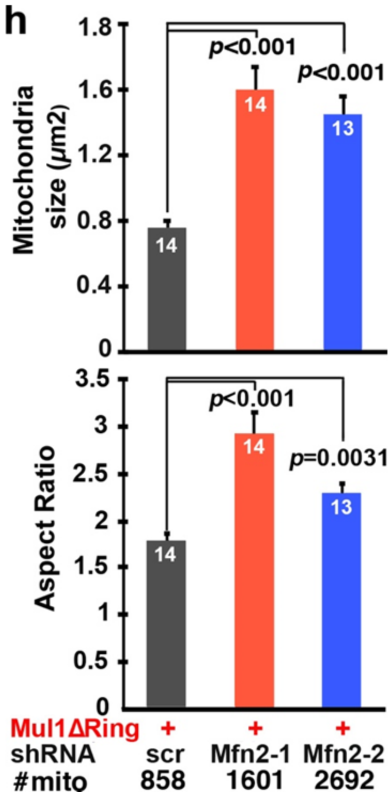

Reviewer #3 (Remarks to the Author):

The authors have adequately addressed this reviewer's concerns.

Reviewer #1 (Remarks to the Author):

The authors addressed the majority of the comments raised by the reviewers. Inclusion of additional controls as suggested strengthens the overall conclusions of the manuscript. I have no further comments.
